# Rational Design of Covalent Organic Frameworks for Oxygen Electrocatalysis: Recent Advances and Mechanistic Insights

**DOI:** 10.1007/s40820-026-02281-x

**Published:** 2026-07-30

**Authors:** Xinyan Zhang, Ting Chen, Zhengping Chen, Gao Chen, Yanping Zhu, Xiaogang Zhang

**Affiliations:** 1 Jiangsu Key Laboratory of Electrochemical Energy Storage Technologies, College of Material Science and Technology, Nanjing University of Aeronautics and Astronautics, Nanjing, 210016 People’s Republic of China; 2 Jiangsu Key Laboratory of New Energy Devices and Interface Science, School of Chemistry and Materials Science, Nanjing University of Information Science and Technology), Nanjing University of Information Science and Technology, Nanjing, 210044 People’s Republic of China

**Keywords:** Covalent organic frameworks, Electrocatalysts, Oxygen reduction reaction, Oxygen evolution reaction

## Abstract

Due to the excessive consumption of fossil fuels and the increasingly severe global environment, the world urgently needs to develop new clean and renewable energy sources. However, these energy sources have intermittency and instability, so it is necessary to vigorously develop efficient and large-scale energy conversion and storage technologies such as fuel cells, water electrolyzers, and metal–air batteries based on the core reactions of oxygen evolution reaction and oxygen reduction reaction. Because of their excellent molecular designability and structural tunability, covalent organic frameworks (COFs) show great potential for applications in this field. In this review, we first classify COF-based electrocatalysts based on the nature of active sites. Subsequently, strategies including structural design and functional synthesis for improving performance of COF-based electrocatalysts in the field of oxygen catalysis are systematically reviewed. Importantly, mechanism discussion of the performance improvement is highlighted. Finally, we put forward prospects for the future research directions, challenges, and development opportunities in this field. This review aims to provide guidance for the development of high-performance COF-based electrocatalysts through critical analysis of existing research, revealing the correlation between material design and mechanism for performance improvement.

## Highlights

- Comprehensive classification of covalent organic frameworks (COFs)-based electrocatalysts based on the nature of active sites.
- Systematic review of structural design and functional synthesis strategies for enhancing oxygen electrocatalysis.
- In-depth mechanistic insights into performance improvement and future perspectives for COF-based oxygen electrocatalysts.

## Graphical abstract

**Figure:**
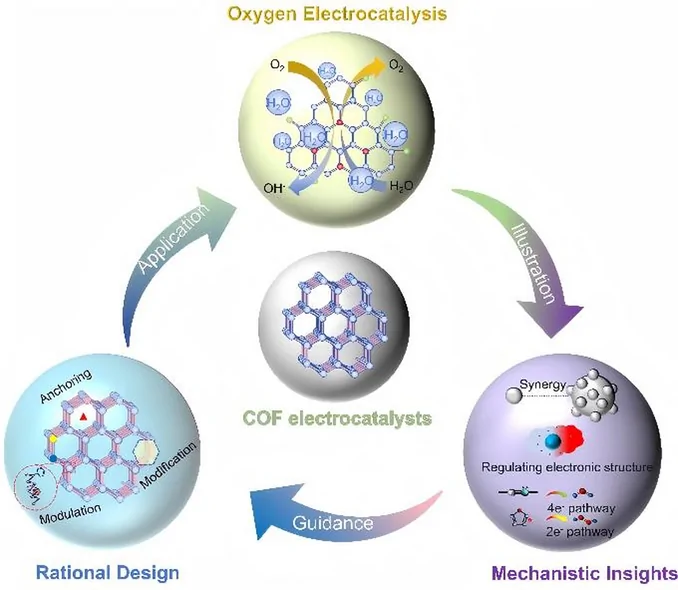

## Introduction

Energy shortage and environmental pollution problems existing in the world are becoming increasingly serious and have become the focus of global attention. Renewable energy sources such as wind energy and solar energy have intermittency and instability, and require efficient and large-scale technologies such as fuel cells, water electrolyzers, and metal–air batteries as bridges for energy conversion and storage [1]. The efficiency and commercialization prospects of these technologies largely depend on the efficacy of their core electrocatalytic processes, especially the oxygen evolution reaction (OER) and oxygen reduction reaction (ORR). Unfortunately, the most efficient catalysts driving these reactions still heavily rely on precious metals such as platinum (Pt), iridium (Ir), and ruthenium (Ru). They are extremely scarce, have high costs, and have insufficient durability, limiting the large-scale development of clean energy technologies. Therefore, research in recent years has mainly focused on developing precious metal-free catalyst materials and further improving the catalytic performance of materials [2, 3].

Covalent organic frameworks (COFs) are a type of crystalline porous polymer formed by organic structural units through strong covalent bonds. Their greatest advantage lies in their excellent molecular designability and structural tunability. Researchers can, as needed, carefully select building units to pre-design and synthesize framework structures with specific topologies, pore channels, and functions that meet the demanding oxygen electrocatalytic environment. In the field of clean energy conversion, electrocatalytic application of COFs has achieved significant progress and some material design principles have been initially established, demonstrating their great potential as the next-generation electrocatalytic platform [4]. Based on these studies, the application scope of COFs is continuously expanding, not only in oxygen electrocatalysis but also in other important reactions such as CO_2_ reduction [5]. It is worth to be noted that the frontier of COF-based electrocatalysis research has entered the rational design stage, with the core being the guidance of experiments through theoretical predictions. Lin et al. [6] first utilized first-principles calculations to study the relationship between the electronic structure of COFs and their catalytic activity, and accurately predicted that a specific COFs could be used for the synthesis of hydrogen peroxide (H_2_O_2_) via the 2e^−^ ORR pathway. As mentioned in the review by Xu et al. [7], this prediction was later verified by experimental results. The review elaborated on how to achieve efficient H_2_O_2_ electro-synthesis by precisely controlling the COF structure.

Although COF-based electrocatalysts have demonstrated advantages and great potential in the field of electrocatalysis, there are still many challenges in directly applying them to efficient OER/ORR. First, most COFs have poor electrical conductivity, which limits the rapid transmission of electrons and affects catalytic activity. Additionally, how to expose and efficiently utilize active sites at high density without destroying the crystal structure of COFs through non-pyrolytic strategies requires further research. Moreover, the long-term stability of materials in harsh electrochemical environments and the complex reaction mechanisms during the catalytic process also need to be further clarified. Finally, using theory to guide experiments represents the cutting edge of this field, which also requires researchers to invest a lot of time and effort to make it more feasible. Furthermore, the structural reconfiguration of the COF-based electrocatalyst that occurs during operation due to the effect of potential, as well as the need to employ advanced in situ characterization techniques to understand this dynamic change, has not yet been fully explored in this field. However, its significance is constantly increasing.

Previous literature reviews have generally explored the application of COF-based electrocatalyst in electrocatalysis [8–14]. This review, however, has its own unique focus: we have systematically classified COF-based electrocatalysts according to their active site types, rigorously evaluated the design strategies for OER/ORR, and no longer merely summarize the performance, but instead deeply analyzed the mechanisms of synergy, electron regulation, and reaction path control. This comprehensive approach enables us to reveal new insights that are not systematically covered in the existing literature, thereby clarifying the purpose and uniqueness of this review.

## Classification of Related COF-Based Electrocatalysts

### Porphyrin-Based COF

Porphyrins are a class of macrocyclic compounds formed by four pyrrole subunits linked through methylene bridges. The N atoms on the porphyrin core can bind to metal ions, forming a M–N_4_ coordination structure that can stably integrate a variety of metal ions, creating an ideal platform for electronic regulation [15]. This structure is widely used in the field of electrocatalysis, especially in reactions involving oxygen intermediates such as oxygen evolution and reduction reactions. Therefore, porphyrins exhibit tremendous potential applications. Researchers have combined well-defined porphyrin active centers with stable COFs to form porphyrin-based COF electrocatalyst, which have been proved to be a highly promising strategy. They covalently bond the porphyrin units as part of the structure and precisely and orderly fix them within the COF.

**Fig. 1:**
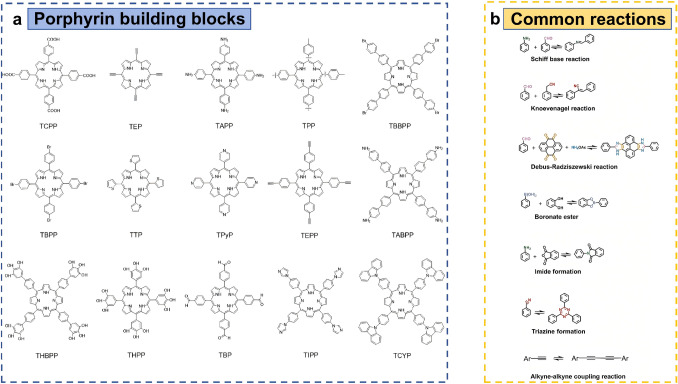
**a** Molecular structure of porphyrin building blocks used for ORR, OER [15]. Copyright 2021, Royal Society of Chemistry. **b** Reported common reactions applied for synthesizing Por-based COFs [16]. Copyright 2025, Wiley

Based on the unique advantages mentioned above, the porphyrin-based COF strategy has rapidly become a focal point in the field of electrocatalysis, showing great potential in this area. Figure 1 illustrates the building blocks and reaction methods that have been reported for the synthesis of porphyrin-based COFs [16]. The remarkable feature of porphyrin-based COFs lies in the fact that the metal centers are pre-introduced before the framework formation: the porphyrin or phthalocyanine macrocycles are functionally connected to the desired metal through four pyrrole nitrogen atoms before the COF polymerization. Therefore, the metal sites are covalently embedded into the COF main chain from the very beginning, forming a clear, planar M–N_4_ configuration, which is uniformly distributed throughout the framework and has a predictable structure. This pre-designed strategy ensures that the coordination environment of each metal center is fixed before synthesis, thereby maximizing the atomic utilization and providing excellent control over the electronic and geometric properties of the active sites. Herein, we will discuss these strategies in detail and their catalytic performance.

The most notable feature of porphyrin-based COFs is their well-defined and customizable active sites. Early research focused on the role of single-metal centers, with cobalt being particularly favored by researchers. Its porphyrin complexes have been proved to be the most efficient oxygen electrocatalysts among all transition metal porphyrin complexes [17]. Studies have shown that three-dimensional topological design can effectively enhance their utilization rate. Compared to traditional two-dimensional layered structures, three-dimensionally interpenetrated porous networks greatly promote the infiltration of electrolytes and the diffusion of oxygen, while maximizing the exposure of Co–N_4_ sites. Electrochemical tests have demonstrated that the ORR/OER performance of this material exceeds that of most previously reported ORR/OER bifunctional electrocatalysts. This work clearly indicates that even without changing the chemical composition of the active center, the catalytic potential of single-metal sites can be fully unleashed through optimization of the macroscopic topological structure [18]. At the same time, another study proves that the local coordination environment in cobalt-based catalysts is key to determining ORR selectivity and activity [19]. In addition, researchers are also looking for other building blocks that are superior to porphyrins. One study cleverly used cobalt corrole as a building block, which has more electron-rich characteristics compared to traditional cobalt porphyrins and combines its unique sawtooth stacking structure to create the optimal microenvironment around a single cobalt site. This special stacking mode not only creates more open channels in three-dimensional space, greatly promoting electrolyte transport [20], but more importantly, it creates an optimal local electrostatic environment and spatial configuration around the active site through specific orientations between layers. This means that future research can choose structural units that are more advantageous than porphyrins, and the design of active centers in porphyrin-based COFs will also shift from metal selection to the regulation of local microenvironments. In addition to the single-metal site activity optimization and microenvironment regulation described above, another very promising strategy is to develop suitable bimetallic active centers to utilize their synergistic effects to achieve efficient bifunctional oxygen catalysis. The core of this strategy is to integrate two metals with different catalytic tendencies into the same framework, allowing them to interact electronically.

The poor conductivity of porphyrin-based COFs is a defect that limits their oxygen catalytic performance. Therefore, globally regulating the electronic structure of the entire porphyrin-based COF skeleton is another core approach to enhance their conductivity and catalytic kinetics. Among various strategies for electronic structure regulation, constructing donor–acceptor structures has been proved to be an effective way to improve charge separation and transport efficiency. Researchers have conducted studies on metal-free D-A COFs [21] to avoid the problem of metal dissolution under harsh OER conditions. This study constructs a completely organic D-A COF using triphenylamine as an electron donor and triazine as an electron acceptor. Electrochemical tests show that this fully organic material exhibits excellent OER activity and astonishing stability. Theoretical calculations reveal that its catalytic activity stems from the high electron density accumulated on the acceptor unit due to strong electron-withdrawing effects, which become active hotspots for adsorbing and transforming OER intermediates. In addition, among the strategies for regulating the electronic structure of COFs, changing their chemical composition is not the only aspect, the physical topological structure of the framework itself is a frequently overlooked but crucial performance tuning angle. A study on the topological isomers of porphyrin COFs provides direct evidence for this [22]. The work compares COFs with the same composition but different topological networks and finds that topology with better scalability and more optimal conjugated pathways can significantly enhance electron mobility.

Excellent powder catalytic materials ultimately need to be transformed into stable and efficient working electrodes through advanced electrode manufacturing technology, and their performance must also be finally verified in actual devices. The traditional slurry coating method requires the use of insulating polymer binders, which introduce significant interface charge transfer resistance and inevitably block some active sites. The successful development of the gas-phase synthesis technique for porphyrin COFs has provided a revolutionary solution to this long-standing technical bottleneck. This technique introduces a new class of porous catalysts, which achieve in situ, uniform, and firm growth of COFs by transporting the precursors of COF via the gas phase and directly undergoing condensation reactions on the surface of conductive substrates under mild heating conditions. The resulting binderless integrated electrodes eliminate the additional resistance and active site masking issues caused by binders. The close physical contact between the COF film and the substrate ensures excellent electronic conductivity and mechanical stability, making them less prone to detachment under long-term operation and high current densities, while obtaining higher crystallinity and purity of COF-based electrocatalysts [23].

In conclusion, porphyrin-based COFs differ from the metal-based COFs discussed in the next section in three fundamental aspects, although both employ the M–N_x_ cooperative center.

Metal incorporation pathway: in porphyrin-based COFs, the metal center is pre-embedded through pre-modification macrocycles before the COF assembly. In metal-based COFs, metal ions are post-modification on the metal-deficient COFs synthesized later.

Coordination environment: the porphyrin-based COF framework provides a strictly defined, co-planar M–N_4_ configuration with fixed bond distances. Metal-based COFs exhibit a chemically adjustable but possibly less uniform coordination geometry.

Catalytic mechanism: porphyrin-based COFs utilize the delocalized *π*-conjugated macrocycle to facilitate the transfer of electrons from the metal center to the metal center, typically promoting the 4e^−^ ORR/OER pathways, with high selectivity. Metal-based COFs rely on the post-synthesis metalization flexibility to create bimetallic or multimetallic cooperative systems, where the electronic effects between adjacent metal centers, such as *d* orbital tuning and charge redistribution, play a dominant role in enhancing the bifunctional activity.

Based on the unique advantages of the pre-functionalized porphyrin system, the following section will explore how post-synthesis metalization on non-porphyrin COF platforms expands the methods for constructing multifunctional, cooperative M–N_x_ electrocatalysts with tunable metal composition and coordination geometry.

### Metal-based COF

The metal-based COFs discussed in this chapter specifically refer to materials that form catalytically active centers by anchoring metal ions such as Co, Fe, Ni, etc., onto the COF framework composed of non-porphyrin organic ligands. This chapter clearly distinguishes and complements the existing chapter on porphyrin-based COFs, where the metal active centers are pre-encapsulated within porphyrin macrocycles, while the focus here is on creating metal active sites on the framework through post-modification or in situ coordination of COFs. This chapter will systematically review the design strategies and electrocatalytic applications of metal-based COFs.

The most direct strategy for constructing metal-based COFs is post-synthesis metallization. This strategy first synthesizes pure COFs containing strong coordinating functional groups such as pyridine, bipyridine, and pyrimidine, then, transition metal ions such as Co^2+^ and Ni^2+^ are precisely fixed at these preset sites through solution impregnation, thereby creating uniformly distributed active centers in the long-range ordered framework.

**Fig. 2:**
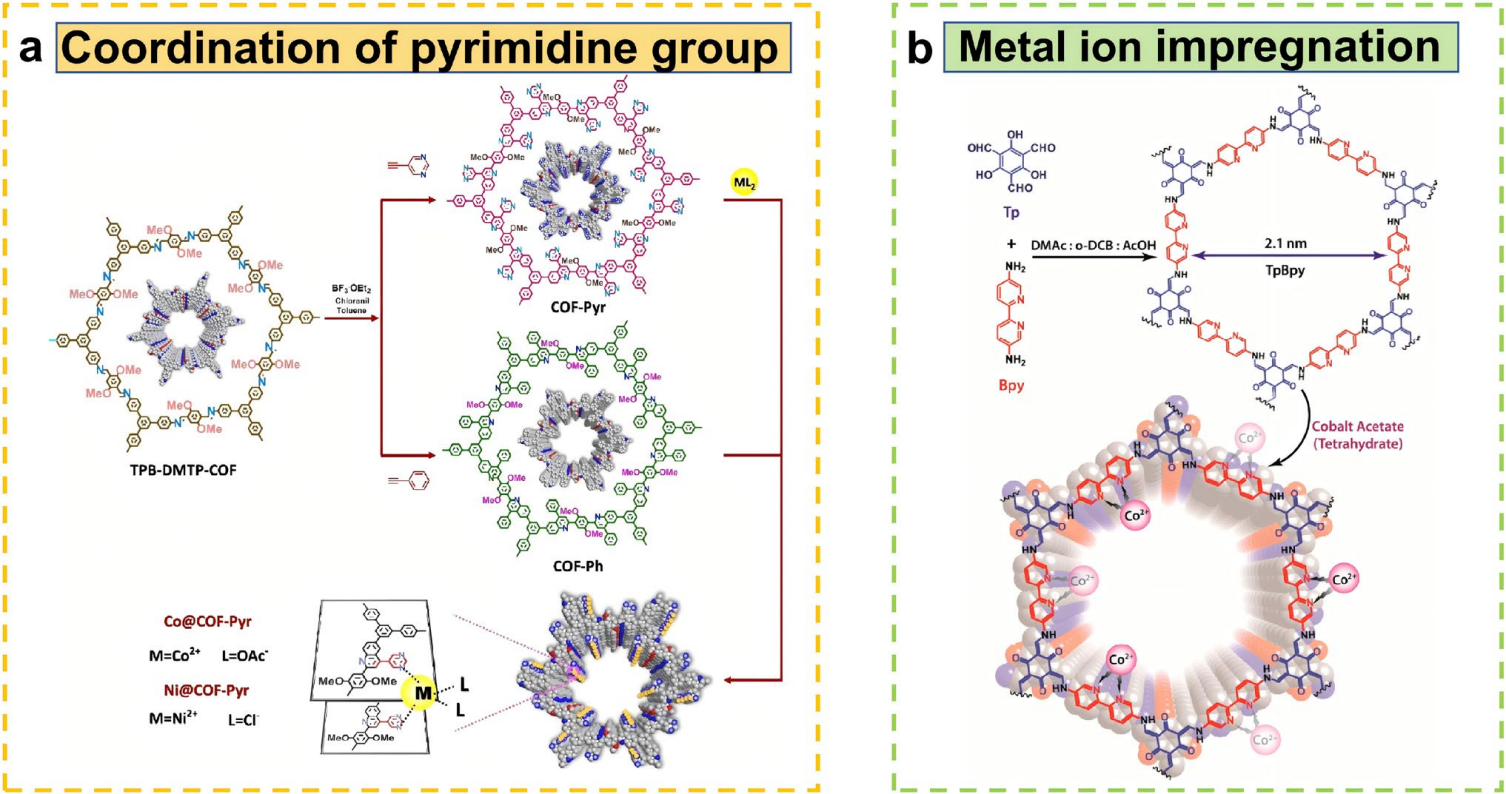
**a** Synthetic route to COF-Pyr, COF Ph, and metal COF composites [25]. Copyright 2021, Wiley. **b** Schematic representation of the synthesis of TpBpy via proton tautomerized Schiff base condensation and Co-TpBpy via Co(II) impregnation [26]. Copyright 2016, American Chemical Society

In recent years, researchers have been exploring ideal coordination units, and bipyridine units have demonstrated their outstanding coordinating ability. Studies have shown that bipyridine units can form well-defined Co–N_4_ sites when coordinated with Co^2+^, and it has been found that this well-defined coordination environment makes its electronic structure closer to the high-valent state required for efficient OER catalysis, thereby exhibiting excellent activity and stability [24]. The success of bipyridine has inspired the exploration of other nitrogen-containing heterocycles, among which the pyrimidine group is a prominent example. Studies have shown that pyrimidine is also an efficient coordination unit. Researchers have fully utilized the coordinating properties of the pyrimidine group and successfully constructed highly active Co sites. Spectroscopic characterization has confirmed the successful introduction of metals and their uniform dispersion within the framework, effectively avoiding the aggregation of active sites (Fig. 2a) [25]. These successful cases of introducing metal sites based on specific functional groups collectively confirm the wide applicability of the post-synthesis metallization strategy. A study further strengthened this conclusion (Fig. 2b) [26]. The cobalt-modified COF prepared by this method maintained long-term stability under harsh electrochemical conditions, proving that the M–N_x_ sites constructed through this strategy possess both high activity and stability.

The materials obtained by the above methods often have insufficient conductivity. A common approach is to conduct high-temperature pyrolysis on COF. However, the pyrolysis process irreversibly damages the crystal structure and definite active sites of COF, thereby losing the core value of COF in electrocatalytic research. A study reported a highly universal pyrolysis-free strategy [27], which can introduce cobalt, nickel, and other metals while perfectly maintaining the high crystallinity and structural integrity of the COF framework. The resulting material not only possesses excellent intrinsic OER activity but, more importantly, its well-defined structure enables researchers to analyze the catalytic mechanism at the atomic level through advanced characterization techniques.

**Fig. 3:**
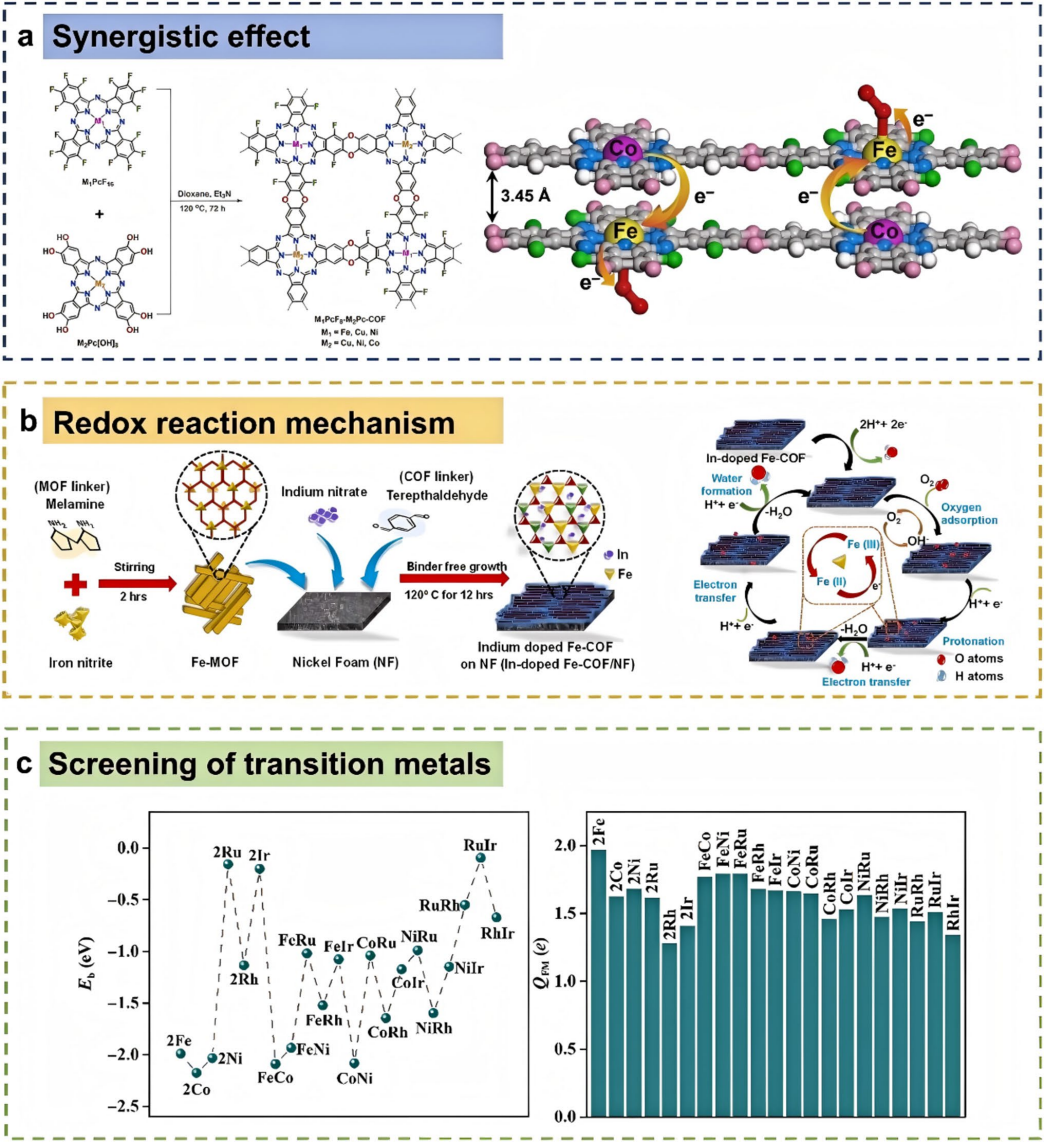
**a** Synthesis of FePcF8-CoPc-COF and schematic diagram of its synergistic ORR mechanism [28]. Copyright 2025, American Chemical Society. **b** Schematic of the synthesis of In-doped Fe-coordinated covalent organic framework (In-doped Fe-COF) on the surface of Ni foam (NF) and schematic diagram of the redox reaction mechanism of In-doped Fe-COF [30]. Copyright 2025, Elsevier. **c** Formation energy of transition metal atoms in 2 T’M-COF, T’M and TM-COF, and the charge transfer from transition metal atoms to the COF substrate calculated by the Bader charge analysis method [32]. Copyright 2022, Tsinghua Press

At the same time, on the basis of successfully constructing single-metal active centers, researchers are no longer satisfied with the catalytic performance of single-metal active sites and have turned to exploring the breaking of performance limits through multi-component and multi-level synergistic effects. Recent studies have introduced a second metal on the basis of a single-metal center to construct a bimetallic cooperative center. They have achieved the long-range ordered and alternating arrangement of Co and Fe atoms in the COF framework, forming a unique checkerboard structure (Fig. 3a). This high degree of order creates the maximum density of cooperative interfaces, significantly improving the ORR performance compared to other materials with randomly distributed metals [28]. This proves that the spatial arrangement of active sites is itself a crucial performance-influencing factor. In addition to the planar arrangement, the interlayer spatial effect also plays an important role. Tan et al. [29] demonstrated that the Ir–N_4_ sites in adjacent layers can regulate the spin state of the Fe–N_4_ sites, thereby achieving excellent oxygen reduction reaction activity. This cooperative effect is not limited to traditional transition metal, the introduction of main group metals is also effective. Researchers innovatively introduced the main group metal indium [30]. Studies have shown that it is not an active center but acts as a powerful electron donor, regulating the electron density of the Fe–N_4_ sites through the conjugated system of the framework, thereby synergistically optimizing the reaction pathways of OER and ORR. This discovery indicates that components not directly involved in the reaction can assist in enhancing performance by fine-tuning the electronic environment. Based on this concept of non-active site regulation, Shi et al. [31] recently demonstrated that adjacent magnesium sites can regulate the spin state of iron monatomic species, thereby significantly enhancing the activity of the oxygen reduction reaction. This reveals a new dimension of spin regulation in bimetallic systems. Subsequently, researchers began to investigate which two metals exhibit synergistic effects to achieve optimal catalytic performance and the underlying mechanism (Fig. 3b). Through density functional theory calculations, researchers systematically compared the behaviors of different bimetallic pairs in OER (Fig. 3c) [32]. This work predicted the potential of Co–Fe bimetallics and revealed that the cooperative mechanism stems from the optimization of the d-band center due to interfacial charge rearrangement.

The optimization of catalytic performance not only concerns the chemical properties of the active sites themselves but also greatly depends on the efficiency of charge transport. A study systematically explained this [33]. This study demonstrates that the chemical structure of the connecting bond directly determines band structure and carrier mobility of the material, which is a key factor affecting ORR kinetics. This confirms that the connecting bonds linking the active sites are as important as the sites themselves. Moreover, the exploration of charge transport can be further deepened to focus on the interaction between electrons and orbitals. A study revealed the deep mechanism of performance improvement [34]. The study pointed out that an asymmetric metal coordination environment can significantly enhance the hybridization between the central metal *d* orbitals and the large *π* bonds of the COF framework, this strong coupling effect establishes an efficient electrical channel between the active sites and the conductive framework. In addition to optimizing the inherent electronic transport channels, an internal electric field can be constructed to actively guide electron flow. Donor–acceptor engineering has become a research hotspot. A study integrated electron donors and electron acceptors represented by cobalt phthalocyanine into the framework, creating a strong intramolecular electric field that drives electrons to flow directionally and rapidly toward the catalytic active centers, achieving a leap in ORR performance [35].

The exploration of COF structures has extended from the innovation of chemical composition to the innovative design of its physical topological network. Recently, a study broke the limitations of traditional COF topologies. By using pseudo-C5 symmetric building units, it successfully constructed an unprecedented Sari topological network. This novel topological structure generates a more complex pore interconnection system and an extremely high specific surface area, greatly promoting the mass transfer of reactants and increasing the accessibility of active sites from a physical structure perspective, directly leading to a significant improvement in ORR performance [36]. Another notable structural design strategy is the adoption of connecting units with extended *π*-conjugated systems. A study demonstrated the dual advantages of benzophenanthroline as an excellent connecting unit. Its quinoline nitrogen atom provides a reliable metal coordination site, while its extended conjugated skeleton acts as an electronic transport channel laid within the COF, simultaneously achieving the construction of active sites and the enhancement of conductivity of the framework, and exhibiting outstanding performance in ORR.

No matter how ingenious the molecular-level design is, converting laboratory powder materials into practical electrodes remains a key challenge on the path to practical application. Traditional electrode preparation processes require mixing COF powder with insulating polymer binders, which seriously sacrifices the utilization rate of active sites and overall conductivity. In this field, a breakthrough is the preparation of self-supporting COF films through interfacial polymerization technology. This technique directly grows large-area, self-supporting, binder-free COF films at the gas–liquid interface, which can be directly used as working electrodes. This unique COF film morphology perfectly addresses the inherent defects of traditional electrode preparation processes, ensuring the full exposure of all active sites and efficient electronic transport pathways. In addition to the innovation in electrode morphology, the greening and efficiency improvement of the synthesis method itself are also important directions for promoting the practical application of COFs. Researchers have developed a supercritical CO_2_-assisted synthesis method [37]. By leveraging the unique physical properties of supercritical CO_2_, this technique can rapidly synthesize high-crystallinity COFs within a few hours, significantly improving efficiency compared to the traditional solvent thermal method that takes several days, and the process is more environmentally friendly, providing new possibilities for the large-scale industrial preparation of COFs.

Faced with the vast library of COF candidate electrocatalysts, the traditional trial-and-error experimental screening method is no longer sufficient. A study systematically expounded the powerful potential of theoretical calculations in this field [38]. This work pointed out that through computational methods such as density functional theory and machine learning, the electronic structure, adsorption performance, and catalytic activity of COFs can be predicted in large quantities in virtual space, thereby providing precise targets for experimental synthesis and significantly improving research efficiency. Additionally, in the future development trend of COF-based electrocatalysts, researchers are beginning to attempt to break the traditional boundaries between COFs and MOFs. Researchers have proposed the concept of covalent metal–organic frameworks [39]. These new hybrid materials aim to combine the metal nodes or clusters of MOFs with the strong covalent bonds of COFs, expecting to simultaneously possess the richness of metal sites in COFs and the excellent stability of COFs, representing one of the future frontiers in framework material design. From a broader perspective, metal-based COFs are demonstrating their multi-functionality in various energy conversion systems. They have broad application prospects as proton conductors or catalyst carriers in fuel cells [40]. Additionally, researchers are studying the integration strategies of these materials in complex energy conversion devices such as overall water splitting from a system-level perspective, which is also a future research direction [41]. Furthermore, extending its application boundary to the field of energy storage demonstrates the great potential of such materials as a universal platform [42].

In order to clearly distinguish the two types of COF-based electrocatalysts discussed in Sects. 2.1 and 2.2, a direct comparison was made in terms of synthesis routes, coordination structures, electronic regulation, dual-function strategies, and conductivity. The results are summarized in Table 1.

**Table 1:** Comparison of porphyrin-based COFs and metal-based COFs

Comparison Aspect	Porphyrin-Based COFs (Sect. 2.1)	Metal-Based COFs (Sect. 2.2)
Synthetic route	Pre-embedded metal centers via functionalized monomers before polymerization [15–22]	Post-synthetic metalation of metal-free COF containing N-donor ligands [23–32]
Coordination structure	Well-defined co-planar M–N_4_ sites, uniform distribution [15, 17, 19]	Tunable M–N2, M–N3, or distorted M–N4 geometries [24, 25, 27]
Electronic regulation	*π*-conjugated macrocycle mainly governs single-site catalysis [20, 22, 73]	Charge redistribution and d-band tuning via adjacent heterometals [28, 30, 31]
Bifunctional strategy	Pairing two different metalloporphyrin units in one framework [18, 63, 82]	Stepwise introduction of two metals for synergistic effects [28, 30, 31, 71, 74]
Conductivity	Moderate to good due to *π*-conjugation, enhanced by fluorination [22, 129]	Poor in pristine state, improved by doping, carbonization, or conductive supports [27, 76, 92]

### Metal-Free COF

This section contrasts sharply with the previously mentioned strategies of constructing well-defined active centers through post-synthesis metalation and other methods. Metal-free COFs have opened up a new path to achieve efficient electrocatalysis by leveraging the intrinsic properties of pure organic backbones [43]. It abandons the reliance on scarce metal resources and instead uses the pure organic framework itself as the source of catalysis, meaning that in the reaction process, it is the organic framework itself or the non-metal heteroatoms incorporated within it that truly play the catalytic role. The metal-free COFs focused on in this chapter derive their catalytic activity from the precise molecular engineering of the electronic structure and local chemical environment of the organic framework. The core of its design lies in: treating the building units and linking bonds of COFs as the source of catalytic function, and through strategies such as linking bond engineering, heteroatom engineering, supramolecular engineering, and morphology and crystallinity control, precisely manipulating the reaction pathways of oxygen electrocatalysis [44].

**Fig. 4:**
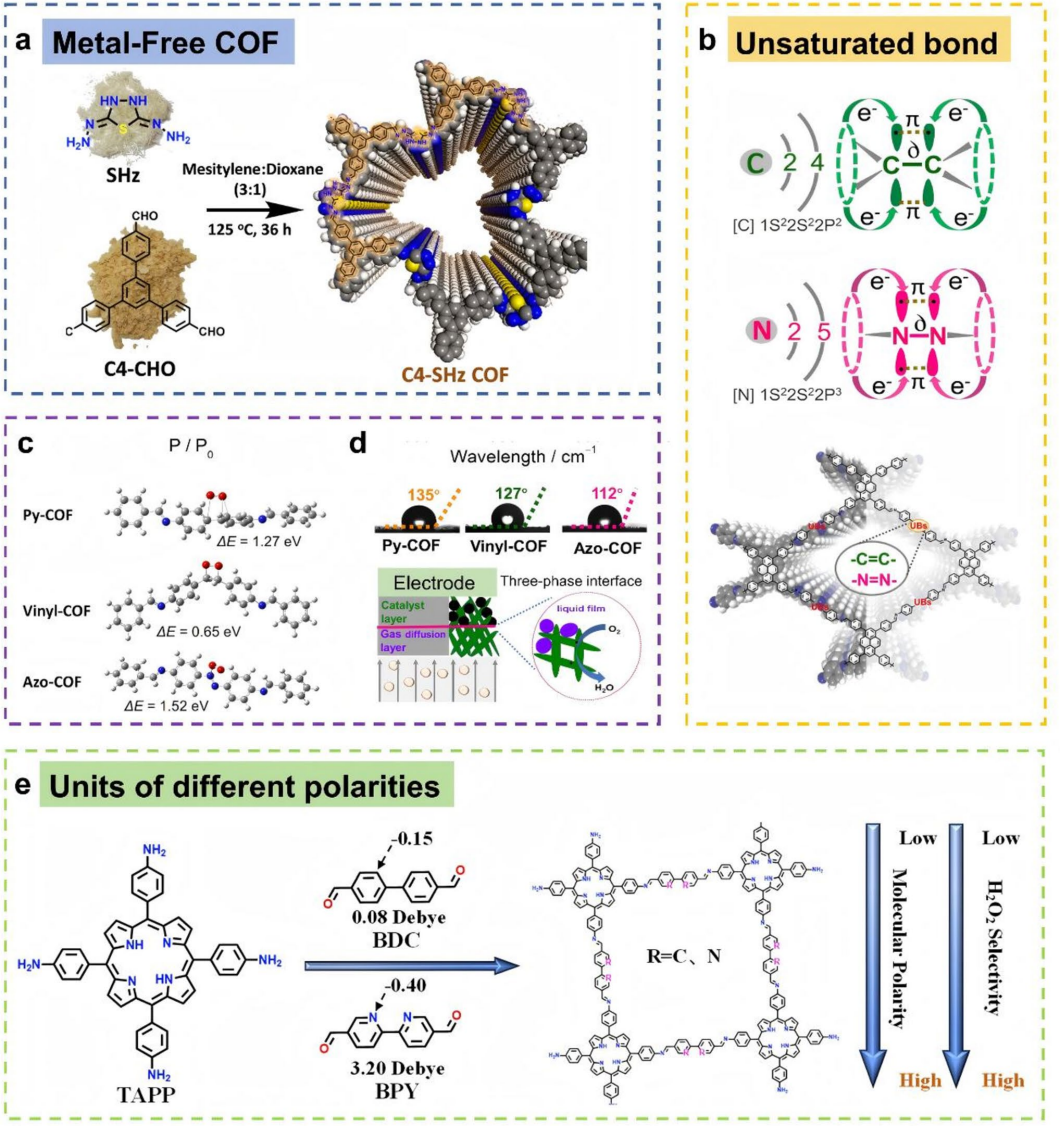
**a** Schematic presentation of the proposed structure COF [43]. Copyright 2020, Elsevier**. b** Electronic structure of ethylene and azo bonds. **c** Unsaturated bond decorated organic framework [44]. Copyright 2022, Wiley. **d** Contact angles, and schematic diagram of the three-phase interface of the ORR reaction process [45]. Copyright 2025 Royal Society of Chemistry. **e** Illustration of COF electrocatalysts customized by building blocks with varying polarity to modulate the 2e^−^ ORR activity [46]. Copyright 2022, Wiley

Heteroatom engineering is the most direct and effective design strategy, which can break the electronic symmetry of the carbon skeleton by introducing atoms with different electronegativities, thereby creating active sites. A representative work in this field innovatively selected a thiadiazole ring containing both sulfur and nitrogen atoms as the key building unit (Fig. 4a). Through detailed density functional theory calculations and in situ spectroscopic characterization, it was confirmed that the synergistic effect between *S* and *N* atoms could induce a strong polarized electric field in the framework, and this polarization effect could transform adjacent carbon atoms into electron-deficient centers, greatly optimizing the adsorption energy of OER reaction intermediates. Notably, this material exhibited a 350 mV overpotential in 1.0 M KOH electrolyte, outperforming most metal-free carbon-based materials, and after continuous operation for 24 h, the activity decay was less than 5%, demonstrating excellent stability [43]. Building on heteroatom engineering, researchers further explored more refined electronic regulation strategies and proposed a breakthrough design concept [44]. This work systematically studied the regulation mechanism of the electronic structure of active centers by different unsaturated bonds (Fig. 4b). Through ingenious molecular design, researchers constructed a series of COF materials containing specific unsaturated bonds and used techniques such as synchrotron radiation X-ray absorption spectroscopy and electron paramagnetic resonance to confirm that the *π* electrons of unsaturated bonds could effectively regulate the spin density and charge distribution of adjacent carbon atoms through conjugation effects. This regulation enabled the optimized COF materials to exhibit a start potential of 0.82 V (versus the reversible hydrogen electrode) in ORR and a four-electron transfer path close to the theoretical value, while also avoiding the structural instability problems that might be caused by heteroatoms.

Meanwhile, based on theoretical guidance, linking bond engineering has become a core means to regulate the electronic structure of COFs, serving as the foundation for controlling the electronic band structure and stability of COFs. Theory-driven design represents the cutting-edge research model in this field. Researchers have deeply investigated the influence mechanism of linking bond polarization on catalytic performance (Fig. 4e) [45]. This work introduced groups with strong electron-withdrawing or donating abilities into the connection keys, thereby constructing permanent built-in dipole moments and charge gradients within the COF. This polarization effect can effectively regulate the adsorption strength of active carbon atoms toward oxygen reduction intermediates, bringing it closer to the peak of the volcano chart. Detailed mechanistic studies revealed that the optimized active sites could significantly reduce the overpotential of the ORR reaction while maintaining an ideal four-electron transfer pathway (Fig. 4c, d). To further expand the design dimension of the connection keys, researchers shifted their perspective from two-dimensional to three-dimensional space. They ingeniously selected electron-rich thiophene units as the core building blocks, leveraging exceptional electron-donating capacity and extended *π*-conjugated system of thiophene, the adsorption energy of oxygen molecules on the material is effectively optimized [46]. More importantly, its unique three-dimensional pore structure greatly facilitated the mass transfer of reactants and products, enabling the material to exhibit excellent ORR activity and stability not only in conventional electrochemical tests but also in real anion exchange membrane fuel cell environments. In line with the three-dimensional structure design, a study demonstrated an innovative method for constructing fully conjugated COFs through a unique interlayer polymerization strategy. Compared with traditional methods, this interlayer polymerization technique can better control the stacking mode and electronic coupling degree of COFs, effectively addressing the key bottleneck of poor conductivity in COF materials. The prepared COF exhibited excellent electronic transport performance and catalytic activity in the ORR reaction, which was attributed to the highly ordered fully conjugated framework providing an ideal channel for charge transfer [47].

As fundamental research deepened, researchers gradually recognized that catalytic activity is not only related to chemical composition but also closely associated with topological structure of the material. A new perspective was proposed. This work successfully constructed a COF material composed entirely of carbon–hydrogen units [48]. By precisely controlling the spatial configuration and connection mode of the building units, inherent electronic asymmetry was generated within the framework. Surprisingly, this pure carbon skeleton without any heteroatoms exhibited an onset potential of 0.78 V and excellent stability in ORR tests. Through a combination of theoretical calculations and experimental verification, researchers revealed that the local electronic density of states rearrangement caused by a specific topological structure was the origin of catalytic activity. This discovery greatly expanded the design perspective of metal-free catalysts. Based on topological engineering, researchers also began to explore supramolecular interaction. A study innovatively introduced the concept of supramolecular chemistry into COF design [49]. The ionic COF nanosheets constructed in this study achieved dynamic regulation of electronic structure of the material through the strong interaction between the inherent cations and the aromatic *π* system in the framework. Detailed mechanistic studies showed that this cation-*π* interaction could continuously extract electrons from the *π* system, resulting in *p*-type doping of the entire framework. This unique electronic structure enabled the material to achieve a current density of 10 mA cm^−2^ at an overpotential of only 320 mV in the OER reaction, performance comparable to many noble metal catalysts.

As researchers gain a deeper understanding of the intrinsic structure of materials, they began to turn their attention to another key factor, the reaction microenvironment. Therefore, researchers pioneered the concept of alkoxyl side-chain engineering [50]. This study systematically investigated the influence of alkoxyl side chains of different lengths and configurations on the catalytic performance of COFs. Experimental results showed that moderate alkoxyl modification could significantly increase the maximum current density of this material is primarily attributed to improved mass transfer processes and enhanced accessibility of active sites. Meanwhile, another study explored the microenvironment from a different perspective. This work achieved in situ monitoring of the dynamic behavior of COF in different solvent environments by designing a precise experimental setup [51]. It was found that parameters such as solvent polarity, electron-donating ability, and viscosity significantly alter catalytic performance by influencing the state of active sites and mass transfer processes.

To expand the functionality of materials, researchers have begun to study COF derivatives, exploring a unique path for material development [52]. By precisely controlling the pyrolysis transformation of COF as a precursor, they successfully prepared F and N co-doped carbon materials. These materials exhibit excellent performance in both ORR and OER reactions, with a bifunctional activity index (ΔE) of only 0.72 V, demonstrating potential for application in practical energy devices.

The most notable development trend in the field of metal-free COF research is the shift from traditional trial-and-error methods to theory-driven rational design. In recent years, researchers have pioneered a theory-driven research approach, conducting systematic density functional theory calculations to pre-evaluate the electronic structure characteristics of various candidate building units and their band structure features after forming COF materials before material synthesis. Based on computational predictions, researchers precisely identified an optimal combination that could form a fully conjugated, highly planar two-dimensional structure. Theoretical simulations indicated that this structure not only has excellent electronic conductivity but also that its entire two-dimensional surface could be an efficient OER active region [53]. Subsequent experimental synthesis perfectly confirmed this theoretical prediction, and the resulting metal-free COF material demonstrated significantly superior activity and stability in the OER reaction compared to the control samples.

### Radical COF

Integrating stable radical species into the COF provides a novel approach to optimizing the electrocatalytic performance of OER and ORR through their unique electronic structure and unpaired electrons. These radical sites can act as efficient electron carriers, significantly accelerating reaction kinetics and optimizing the adsorption energy of intermediates [54]. Redox-active sites achieve charge storage and regulation of catalytic intermediates through reversible electron transfer, while radical sites accelerate redox kinetics with the high reactivity of unpaired electrons. Early research mainly focused on the construction of redox-active sites, introducing conjugated redox units into COFs through skeleton engineering to achieve bifunctional catalysis of ORR and OER, with half-wave potentials and current densities superior to traditional metal-free COFs, confirming the decisive role of active sites in electrocatalytic performance.

The construction of highly ordered free radical COFs can generally be divided into two strategies: (i) directly from free radical monomers, for example, the COF based on polytrimethylsilane (PTM) by Wu et al. [54], where stable free radical building units directly polymerize to form the framework. (ii) generation or fixation of free radicals after synthesis, such as the work by Xu et al. [55] (fixing poly-free radicals on the pore walls) and the work by Chen et al. [56], that is, embedding TEMPO free radicals from bottom to top in the three-dimensional COFs. In addition, emerging strategies have also been developed, such as thermal-induced free radical formation, where thermal treatment triggers the generation of persistent free radicals while maintaining the crystallinity of the framework [57]. Similarly, the thermal cyclization of anti-fold motifs rich in alkyne provides a mild method to introduce persistent free radicals without affecting the porosity [58].

Early pioneering work focused on the fundamental concept of constructing two-dimensional *π*-conjugated radical frameworks. For instance, Wu et al. [54] successfully designed and synthesized a COF based on stable radical building units such as PTM derivatives. This study confirmed the presence of a large number of stable unpaired electrons in the material through electron paramagnetic resonance spectroscopy, marking a breakthrough from concept to reality for “covalent organic radical frameworks.” More importantly, this study preliminarily verified the application potential of such materials in electrocatalysis, demonstrating considerable catalytic activity and unique electron transfer characteristics when used as an ORR catalyst. During the same period, Xu et al. [55] developed an alternative general strategy by using post-modification methods to fix the accessible poly-free radicals onto the pore walls of traditional COFs. These poly-free radical functionalized frameworks achieved rapid and reversible redox reactions through the fixation of the channel walls, thus demonstrating excellent capacitive energy storage performance. Although this work focused on capacitive energy storage rather than electrocatalysis, it established a general method for introducing high-density free radical sites into COFs while maintaining their ordered pore structure.

Although these early studies mainly relied on post-synthesis radical immobilization techniques, other methods based on a bottom-up approach have also emerged. Chen et al. [56]. reported the first batch of three-dimensional COFs based on radicals, named JUC-565 and JUC-566, where TEMPO radicals were uniformly immobilized on the channel walls through a bottom-up synthesis strategy. These three-dimensional radical COFs achieved an excellent conversion rate of up to 132 h^−1^ during the aerobic oxidation of alcohols to aldehydes or ketones, surpassing other TEMPO-modified catalytic materials tested under similar conditions, and also demonstrated excellent reusability. This work expanded the structural dimension of radical COFs and demonstrated their potential in heterogeneous catalysis. The above examples of COFs treated with TEMPO not only demonstrate the feasibility of synthesis but also open up a broader design space. In this context, Lee et al. [59] conducted a comprehensive review of the strategies for using MOFs and COFs containing TEMPO in catalytic applications. This review systematically summarizes the strategies for immobilizing TEMPO radicals onto the porous framework—from post-synthesis modification to bottom-up integration, and emphasizes how the synergistic effect of TEMPO chemistry and the tunable porosity and crystallinity of COFs has achieved significant catalytic progress, including improving recyclability and selectivity [REF_X5]. The authors also discussed the unique stability of TEMPO radicals, which is attributed to their structural characteristics, and how molecular modifiability allows for reasonable modifications to the COF scaffold. This review covers MOFs and COFs, and its systematic classification of synthesis strategies and catalytic performance provides valuable references for designing next-generation radical COF catalysts, especially in oxygen electrocatalysis.

Building on this, subsequent research aimed to deeply explore the precise regulation mechanism of radical sites on the ORR pathway. Xu et al. [60] reported a COF with well-defined carbon-centered radicals (R-COF) as a prime example. Compared with pioneering work, this study successfully synthesized a COF with well-defined carbon-centered radicals and directly applied it to the efficient ORR process. Through a high and stable single-peak signal in electron paramagnetic resonance spectroscopy, they confirmed the presence of a large number of carbon-centered radicals in the material. Electrochemical tests showed that R-COF exhibited excellent ORR activity in alkaline media, with an onset potential as high as 0.92 V and an electron transfer number close to 4.0, outperforming many non-radical control samples. More importantly, theoretical calculations revealed the microscopic mechanism of its high performance: radical sites significantly reduced the energy barrier of the rate-determining step from O_2_ to OOH*. In addition to the carbon-centered free radical system, the electronic origin of the free radical COF activity was also explored through other free radical—framework combinations. For example, in the double free radical COF generated from the metastable conversion of pyridine N-oxide, the electron-attracting bistrimamide group and the *π*-conjugated unit work together to stabilize the double free radical species and promote its catalytic activity in the dehydrogenation reaction [61]. More broadly, combining the donor–acceptor motif with the free radical site can produce a synergistic polarization effect, further optimizing the electronic structure of the active center [62]. These observations collectively indicate that the free radical site is not merely a passive spectator. Instead, it actively reshapes the electronic environment of the COF through spin density redistribution and cooperative electronic interactions with the surrounding framework. On this basis, the great potential of this strategy was further extended to the more challenging field of bifunctional electrocatalysis. Liu et al. [63] ingeniously integrated diamine derivatives into a cobalt porphyrin framework to construct a COF with multiple redox-active sites. This material can efficiently catalyze both ORR and OER processes. In OER tests, it only requires a 420 mV overpotential to reach 10 mA cm^−2^, while its ORR half-wave potential reaches 0.80 V. This work indicates that combining radical chemistry with multi-site cooperative catalysis is a powerful strategy for developing high-performance, bifunctional oxygen electrocatalysts.

In addition to the identification and application of the active sites, the understanding of how the active groups are stabilized in the COF structure is of crucial importance for rational catalyst design. The high stability of the persistent active groups in COF is usually achieved through the following three complementary strategies: (i) achieving electronic stability through extended *π* bonding, which delocalizes the unpaired electrons and reduces the spin density at any single atom. (ii) Spatial protection, that is, using large-volume substituents or rigid framework structures to prevent bimolecular coupling reactions. And (iii) hydrogen bonds or coordination interactions between the active groups and adjacent chemical functional groups, which further inhibit the quenching of the active groups. For example, Gu et al. demonstrated that lower total energy and nonzero spin density are two key factors ensuring the persistence and polarization of spin distribution of the active groups in COF, which can be achieved by thermal treatment without sacrificing crystallinity [57].

Apart from their specific applications in ORR and OER, the broader potential of COFs with redox activity in the field of synthetic electrochemistry is gradually being recognized. Kim et al. [64] recently provided a forward-looking review article that connects the fields of organic synthesis, electrochemistry, and polymer science. They pointed out that organic materials with redox activity, including COFs, due to their structural tunability, flexibility, and compatibility with various electrolytes, are highly suitable for organic electrochemistry, which is an emerging field in fine chemical synthesis, thus having unique advantages. In particular, the authors emphasized that the applications of organic electrode materials are not limited to traditional energy storage, but have expanded to photocatalytic reduction, electrocatalysis, and sustainable synthesis of complex molecules. This review pointed out new directions that need to be explored to unleash the potential of COFs with redox activity as heterogeneous electrocatalysts, indicating that their role in energy conversion and organic synthesis in the future will be far more diverse than currently understood.

Overall, recent experimental and computational studies on free radical COFs have revealed several key structure–property relationships: (i) Increasing the *π* conjugation length usually enhances the stability of the free radical, but may reduce the electrochemical activity due to the excessive stability of the free radical center [65]. (ii) Optimizing the interlayer stacking, for example, weakening the stacking in thin-layer two-dimensional COFs is a powerful means to balance the accessibility of the free radical and the structural stability, and this has been demonstrated through thickness-dependent regulation of the α-C free radical reactivity and stability [66]. (iii) Introducing electron-withdrawing substituents near the free radical center can fine-tune the spin density distribution, thereby regulating the binding affinity to key reaction intermediates [67].

Based on the detailed discussions in Sects. 2.1–2.4, Table 2 summarizes the representative active sites, advantages and limitations of COFs based on porphyrin, metal, metal-free and radical. Furthermore, in order to quantitatively summarize the electrocatalytic performance of these four types of COFs, the key indicators (including the half-wave potential of ORR, the overpotential of OER, and the electrolyte conditions) are summarized in Table 3.

**Table 2:** Advantages and limitations of four types of COF-based electrocatalysts

COF Type	Representative active site	Advantage	Limitation	References
Porphyrin-based COF(2.1)	M–N_4_(where M = Co, Fe, etc.) or metal-free porphyrins	The active site is clearly defined, the electronic structure is adjustable, and it is easy to achieve bimetallic synergy	Poor conductivity, insufficient stability in some aspects, and demanding synthesis conditions	[15–22]
Metal-based COF)(2.2)	Post-modified or in situ coordinated metal ions (such as Co, Fe, Ni, In, etc.)	The metal loading method is flexible and can incorporate main group metals for regulation. The theory can guide the design	Metals are prone to leaching. Thermal decomposition disrupts the structure, and the conductivity needs to be improved	[23–32]
Metal-free COF(2.3)	Aromatic atoms (such as N, S, F) or the organic framework itself	Low cost, environmentally friendly, diverse structure, no metal pollution	The activity is lower than that of the optimal metal-based material, the oxygen evolution reaction (OER) is weaker, and the conductivity is poor	[43–53]
Radical COF(2.4)	Stable free radicals and redox-active units	Unpaired electron acceleration dynamics can lower the energy barrier and enhance the dual-function potential	The long-term stability of free radicals remains to be verified, and the control of density is difficult	[54–62]

**Table 3:** Performance indicators of four types of COF-based electrocatalysts

COF type	Material	Reaction	ORR *E*_1/2_ (V Vs. RHE)	Overpotential η@10 mA cm^−2^ (mV)	Electrolyte	References
Porphyrin-based COF	3D porphyrin COF (SUZ-101-Co)	ORR/OER	0.78	240	0.1 M KOH	[18]
	CoTAPP-PATA-COF	ORR/OER	0.80	420	0.1 M KOH	[63]
	Co-Por-BTD	ORR	0.75,0.83	-	0.1 M KOH	[82]
	Fluorinated Co-porphyrin COF/GO	OER	–	261	1.0 M KOH	[129]
	Checkerboard CoFe-COF	ORR	0.87	–	0.1 M KOH	[28]
	CoO_x_@NC-800	ORR	0.89	–	0.1 M KOH	[68]
Metal-based COF	Fe, Co–N–C (pyrolysis-derived)	ORR/OER	0.85	330	0.1 M KOH	[92]
	Ir@COF-C_4_N	OER	–	280	1.0 M KOH	[77]
	In-doped Fe-COF	ORR	0.86	–	0.1 M KOH	[30]
	Thiadiazole-based COF	OER	–	320	1.0 M KOH	[43]
	Ionic COF nanosheet (cation-*π*)	OER	–	275	1.0 M KOH	[49]
Metal-free COF	Piperazine-integrated COF (PD-COF-OH)	ORR	0.76	–	0.1 M KOH	[81]
	Oxazole-linked COF	ORR	0.75	–	0.1 M KOH	[108]
	COF-β (thienyl isomer)	ORR	0.76	–	0.1 M KOH	[115]
	Carbon-centered R-COF	ORR	0.82	–	0.1 M KOH	[60]
Radical COF	TEMPO-based 3D COF (JUC-565/566)	–	–	–	–	[56]
	PTM-based 2D CORF	ORR	0.80 (vs Fc^+^/Fc)	–	0.1 M KOH	[54]
	TEMPO-functionalized COF (post-modification)	–	–	–	–	[55]

## Strategies for Improving Performance of COF-Based Electrocatalysts

After systematically elaborating on the structures and properties of various COFs materials such as porphyrin-based, metal-based, and metal-free COFs, it is recognized that they themselves are ideal platforms for designable electrocatalysis. However, their intrinsic properties, especially conductivity, the intrinsic activity and stability of active sites, often fail to meet the demanding requirements of efficient ORR and OER. Therefore, to fully exploit the potential of COFs in energy conversion, it is crucial to develop effective strategies for performance enhancement. As pointed out above, reducing the material dimension to two-dimensional is one of the key approaches to unlocking their catalytic potential, which can expose abundant active sites and significantly improve mass transport efficiency [69]. Meanwhile, beyond the single dimension regulation, a broader molecular engineering scope, that is, customizing the oxygen electrocatalytic performance of materials through precise atomic and molecular-level design, offers more possibilities for the development of COF electrocatalysts [8]. Guided by this concept, single-atom metal catalysis, as an outstanding example of molecular engineering, has achieved the maximization of metal active sites in COFs through various anchoring strategies [70]. At the same time, to maintain the clear structural advantages of COFs while pursuing high performance, the development of non-pyrolytic COF-based electrocatalyst has also become an important research direction [71]. Based on this, this chapter will systematically review the cutting-edge strategies for enhancing the oxygen electrocatalytic performance of COF-based electrocatalyst in recent years. According to the treatment methods and modification timings of COF structures, these strategies generally fall into two categories: structural design and functional synthesis.

### Structural Design

**Fig. 5:**
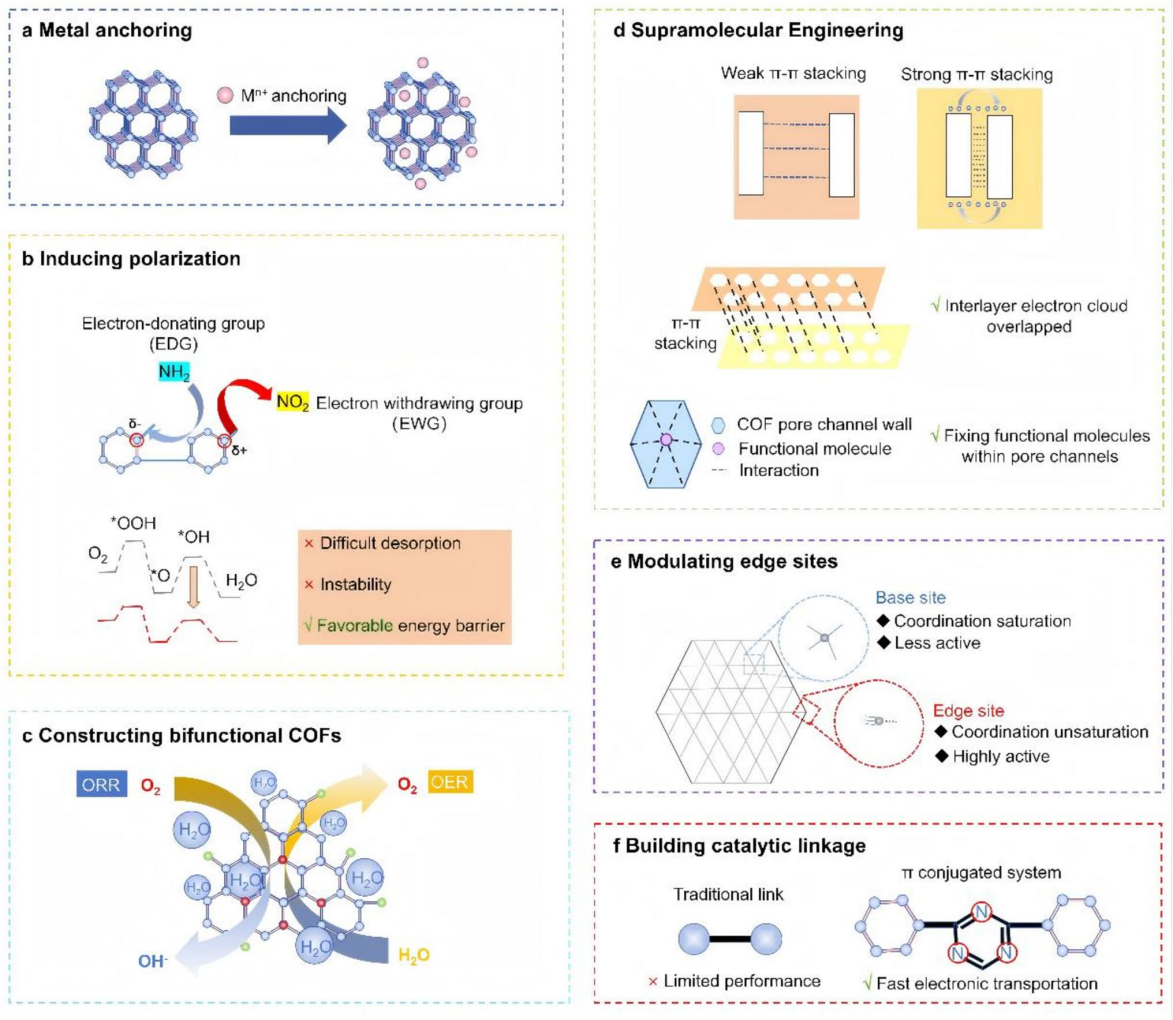
Schematic illustration of key strategies to enhance the oxygen electrocatalytic performance of COFs. **a** Metal anchoring. **b** Inducing polarization. **c** Constructing bifunctional COFs. **d** Supramolecular engineering. **e** Modulating edge sites. **f** Building catalytic linkage

In the second part, we have already discussed the basic categories and synthesis methods of COF. Now, we will turn to the structural design strategies aimed at enhancing the oxygen electrocatalytic performance. These methods aim to unleash the catalytic potential of COF without altering its basic chemical composition through molecular/supramolecular engineering and nanoscale structure regulation. Typically, this approach aims to explore the catalytic potential from within the COF without altering its basic chemical composition through sophisticated molecular or supramolecular engineering. It includes precise design of active centers at the atomic or molecular scale (such as metal anchoring and inducing polarization), as well as regulation of its physical structure at the nanoscale or macroscale such as constructing bifunctional COFs, supramolecular engineering, modulating edge sites and building catalytic linkage (Fig. 5).

#### Metal Anchoring

The metal anchoring strategy is one of the most direct and effective methods for constructing clear and highly efficient active sites. The core of this strategy lies in precisely fixing metal species on specific coordination sites of the COF, thereby creating catalytic centers with high intrinsic activity and excellent stability. Depending on the type, quantity, and spatial configuration of the anchored metals, this strategy presents a rich range of research dimensions. This strategy aims to combine the high activity and selectivity of molecular metal complexes with the high stability of heterogeneous catalysts, thereby creating ideal single-atom catalysts.

**Fig. 6:**
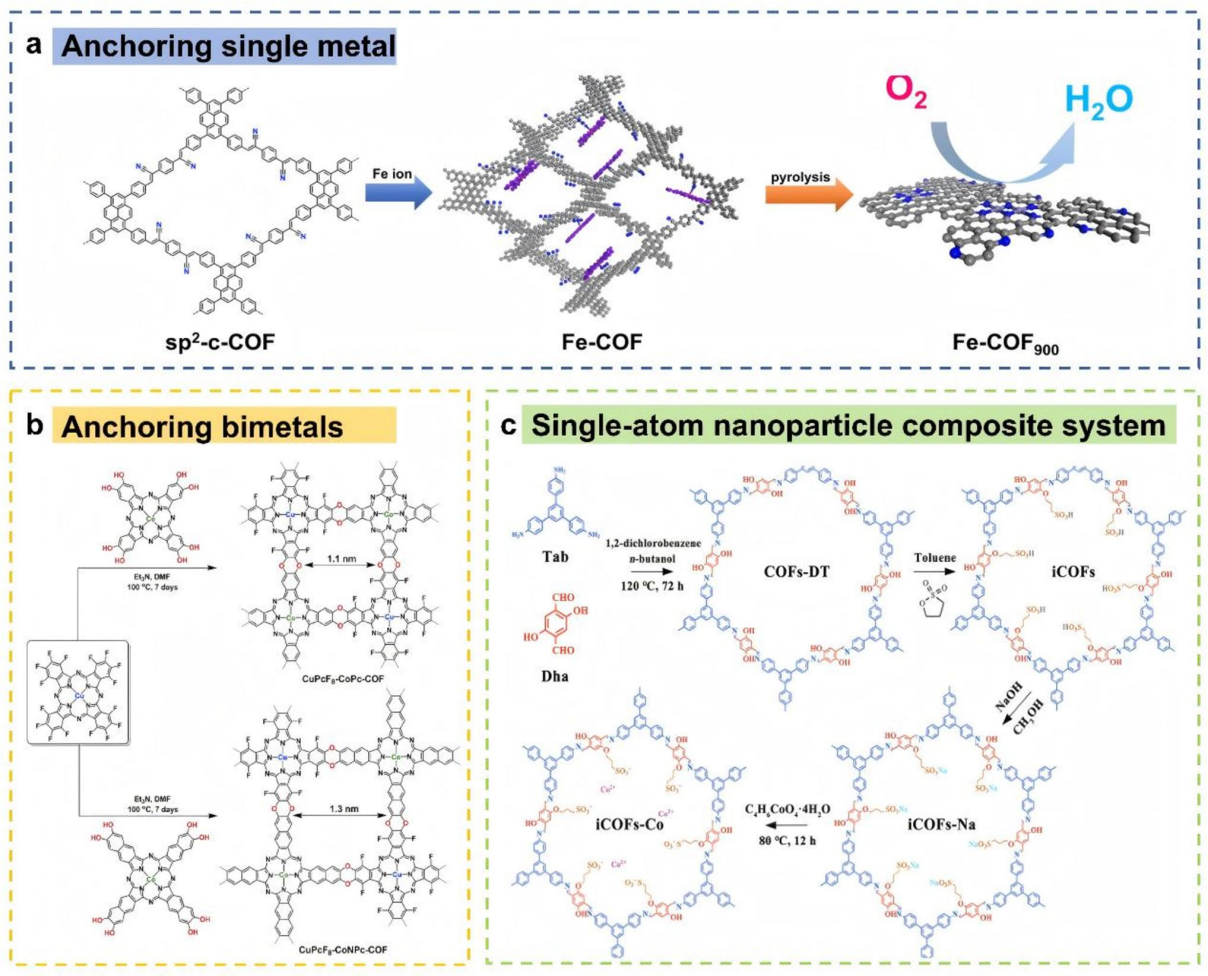
**a** Synthesis of Fe-COF900 from sp^2^-c-COF [72]. Copyright 2022, Elsevier. **b** Schematic representation for the synthesis of CuPcF8-CoillustrationPc-COF and CuPcF8-CoNPcCOF under typical solvothermal conditions [74]. Copyright 2021, American Chemical Society. **c** Schematic for the synthesis of iCOFs-Co from Tab and Dha [75]. Copyright 2022, Wiley

The synthetic pathways for achieving metal anchoring can be divided into two main types: one is to directly use metalized monomers (such as the metal porphyrins as described in Sect. 2.1) for polymerization, where metal atoms are directly constructed as structural units into the framework. The second is the post-synthesis anchoring method, where in the synthesized COFs with empty coordination sites, metal ions are coordinated to the preset sites through solution infiltration. Single-metal anchoring is the cornerstone for constructing basic active sites. By designing monomers with clear coordination cavities, metal ions can be precisely fixed. In addition, the regulation of the metal site structure can even be advanced to the regulation of the distance between active sites, by constructing ultra-close double-atom pairs to break the traditional limitations of developing single-atom catalysts. A breakthrough work was accomplished by Yang et al. [72] who used fully conjugated COF as a precursor and derived carbon materials carrying ultra-close Fe–N_4_ sites (Fig. 6a). The innovation of this study lies in the researchers designing and synthesizing a special COF precursor with a fully conjugated structure, whose rigid framework structure can effectively prevent the migration and aggregation of metal atoms during the thermal decomposition process. Then, by precisely controlling the metal loading amount and thermal decomposition conditions, iron atoms were successfully stably present in the carbon matrix in a paired form, with an atomic distance of approximately 2.52 Å, most importantly, through the combination of synchrotron radiation technology and theoretical calculations, the existence and electronic structure of the closely-packed Fe active sites were clearly confirmed in the COF-derived materials. This unique site configuration creates an optimized electronic environment through strong d-orbital electron coupling, allowing it to adsorb OOH* intermediates at exactly the apex position of the volcano diagram. Experimental results show that the intrinsic activity of this closely-packed atomic site is 3.2 t that of the traditional Fe–N_4_ single-atom site, and the overpotential is reduced by approximately 26 mV. In addition to being able to control the distance between atoms, the recent research by Zhang et al. [73] also indicates that introducing local micro-strain can also optimize the asymmetric iron monomer sites for the oxygen reduction reaction, thereby further expanding the structural tunability of the metal-anchored covalent organic frameworks.

Based on a single-metal site, introducing a second metal to construct a dual-center cooperative catalysis is an effective way to further improve performance. As reported by Yue et al. [74] in the stable bimetallic poly-porphyrin COF, the framework contains two different metal centers. The characteristic of this study lies in the adoption of an innovative copolymerization strategy, where porphyrin monomers with different metal centers are connected through covalent bonds to form an ordered two-dimensional framework structure (Fig. 6b). This design enables the uniform distribution and close adjacency of the two metal centers at the atomic scale, creating favorable conditions for their electronic interaction. Through systematic characterization and theoretical calculations, researchers found a significant electron transfer phenomenon between the two metal centers, and this electron reconfiguration effect effectively optimizes the adsorption energy of the active sites for oxygen intermediates. Compared with single-metal COFs, this dual-metal structure optimizes the reaction pathway through the synergy of electronic and geometric effects, thereby demonstrating superior overall catalytic performance. The overpotential of the ORR reaction is reduced by approximately 40 mV, and the selectivity of the reaction is improved to nearly perfect 4-electron pathways. This study fully demonstrates the advantages of the dual-metal design.

Beyond the atomic-level dispersion, constructing a single-atom-nanoparticle composite system can integrate the advantages of different sites and achieve functional complementarity. For instance, Guo et al. [75] reported an ionic COF-derived carbon material that simultaneously contains cobalt single atoms (Co–N_4_) and cobalt nanoparticles. This study ingeniously utilized the unique charged framework structure of ionic COF to achieve the highly dispersed and fixed cobalt precursor through electrostatic interactions (Fig. 6c). During the subsequent thermal decomposition, this pre-organized structure guided the formation of different forms of cobalt species: some cobalt atoms coordinated with nitrogen atoms in the framework to form Co–N_4_ single-atom sites, which were proved to be the main active centers for the ORR reaction. While another part of cobalt was reduced and aggregated to form ultra-small cobalt nanoparticles, which exhibited higher intrinsic activity for the OER reaction. The synergy of the two parts endowed the material with outstanding dual-function oxygen catalytic performance, manifested in the ORR/OER potential difference (ΔE) of the material in 0.1 M KOH electrolyte of only 0.78 V, far lower than the benchmark catalyst based on precious metals. Meanwhile, Zhou’s team [76] also successfully anchored platinum single atoms and platinum nanoparticles together on a COF-derived upright carbon material. This study designed a COF film with a vertical orientation, which formed a unique vertical carbon nanosheet array structure after thermal decomposition, providing an ideal carrier for the anchoring of different forms of platinum species. The single-atom sites optimized the adsorption of reaction intermediates, while the nanoparticles promoted charge transport. This synergistic effect enables the material to achieve a specific activity 8.5 t that of commercial Pt/C, while also demonstrating excellent stability. These works indicate that the metal anchoring strategy can flexibly create multi-component catalytic systems. The advantages of the metal anchoring strategy not only lie in constructing the intrinsic active COF, but also in its ability to serve as an ideal precursor for in situ thermal decomposition to produce structurally exquisite derivative catalysts. A typical study [68] demonstrated this process. The researchers first precisely anchored Co^2+^ on the carbonyl site of the TRIPTA-COF framework to form the TRIPTA-Co precursor. Subsequently, at 800 °C, the precursor was successfully transformed into a unique composite structure, with hollow Co_3_O_4_ and CoO nanoparticles embedded in the layered nitrogen-doped carbon nanofiber. This derivative structure brought multiple advantages: the layered carbon fibers inherited the structural characteristics of COF, providing high conductivity and mass transfer channels. The hollow nanoparticles greatly exposed the active sites. Electrochemical tests showed that CoOx@NC-800 as a dual-function catalyst had an ORR half-wave potential of up to 0.89 V and no degradation after 30,000 cycles, and the charging and discharging potential difference of the zinc–air battery based on its assembly (0.70 V) was also superior to the benchmark catalyst based on precious metals. Theoretical calculations revealed that the heterogeneous interface is the root of high performance, which can optimize the adsorption of OOH* intermediates and lower the reaction energy barrier.

The application of metal anchoring through theory-guided experimental design demonstrates an important transition from empirical exploration to rational design. For instance, the Zhang team [77] conducted theoretical calculations to screen and guide the synthesis of COF-C_4_N nanosheets that were anchored with a single Ir site. This material became an efficient electrocatalyst for OER, perfectly combining the metal anchoring strategy with theoretical predictions. Firstly, through density functional theory calculations, Ir was selected as the optimal OER active center from over ten candidate metals, with a predicted theoretical overpotential of only 220 mV; subsequently, based on theoretical guidance, a COF-C_4_N carrier with a specific pore structure was designed and synthesized, utilizing its abundant pyridine nitrogen sites to achieve atomic-level dispersion of the Ir atoms. Finally, the obtained catalyst achieved an overpotential of 268 mV at a current density of 10 mA cm^−2^, highly consistent with the theoretical prediction, demonstrating performance surpassing that of most reported OER catalysts.

In conclusion, the metal anchoring strategy, leveraging the clear and designable coordination environment of the COF platform, successfully achieved the precise construction of various active structures from single metals to dual metals, and from isolated single atoms to single atom/nanoparticle complexes. The metal anchoring strategy demonstrates rich research dimensions and strong design flexibility. Future research on the metal anchoring strategy will focus more on exploring more novel metal site configurations on the basis of COF and deeply revealing the cooperative mechanisms among multiple centers.

#### Inducing Polarization

The catalytic performance of COFs fundamentally depends on their electronic structure. Induced polarization is one of the core strategies for regulating the electronic structure of COFs and enhancing their electrocatalytic performance. The core idea of this strategy lies in the ingenious molecular design to disrupt the uniform distribution of the electron cloud in the conjugated framework of COFs, thereby creating a strong and directional built-in electric field, or polarization field. This electronic structure reconstruction can effectively optimize the adsorption strength of the active sites for reaction intermediates, significantly lowering the energy barrier of the ORR or OER, and improving catalytic activity and selectivity [78]. Depending on the implementation methods, the induced polarization strategy mainly includes the following design strategies.

**Fig. 7:**
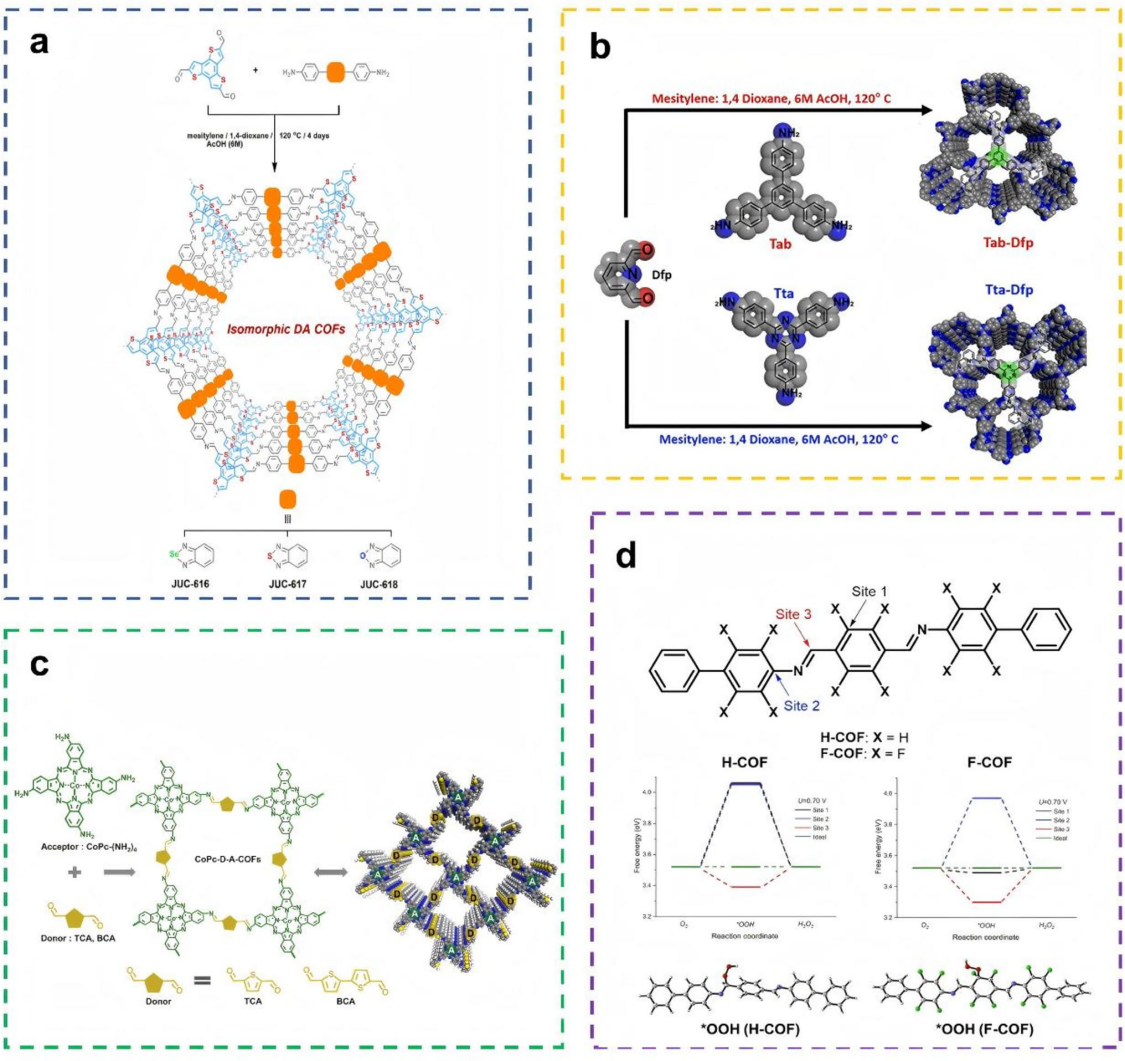
**a** Synthesis and chemical structures of isomorphic BTTCOFs (JUC-616, JUC-617, and JUC-618) [79]. Copyright 2023, Wiley. **b** Synthetic scheme of Tab-Dfp and Tta-Dfp [87]. Copyright 2024, Wiley. **c** Schematic synthetic process and simulated structure of two CoPc-D-A-COFs [35]. Copyright 2023, American Chemical Society. **d** Active sites for 2-electron redox reactions in the hydrogenation and fluorination COF models [80]. Copyright 2023, Wiley

The basis of induced polarization stems from the most fundamental chemical composition of the COFs, namely the regulation of atoms and chemical bonds. Heteroatom doping is the most direct way to generate local polarization by taking advantage of the intrinsic electronegativity differences of atoms. The principle is to utilize the inherent electronegativity difference between heteroatoms and carbon atoms to generate permanent and localized dipole moments on C-X (*X* = N, O, S, etc.) bonds. A comprehensive review of metal-free COFs with precise heteroatom incorporation has fully demonstrated this point (Fig. 7a) [79]. For instance, when a heteroatom with a higher electronegativity, such as a nitrogen atom in pyridine, replaces a carbon atom in the framework, it will pull the electron cloud through the inductive effect, causing the adjacent carbon atom to present an electron-deficient state, thereby becoming a potential active site for ORR. By precisely controlling the type, position, and quantity of heteroatoms, the intensity and range of the local polarization field can be finely tuned, thereby establishing a clear model of how changing the type of heteroatom conditions the polarization pattern and ultimately controls the electron density of the active sites to enhance ORR performance. This provides the most fundamental and powerful principle for the rational design of catalysts.

In addition to the above two strategies of atomic and bond-level regulation, introducing functional groups with strong electronic effects, especially those with the highest electronegativity such as fluorine atoms, can achieve more intense and directional polarization. Researchers have synthesized fluorinated COFs with strong polarization effects by using highly fluorinated building units containing trifluoromethyl groups (Fig. 7d). Fluorine atoms and related functional groups strongly pull the electron cloud of the entire conjugated framework through the powerful induction effect of *σ* bonds and *π* systems, thereby generating a uniform and powerful built-in electric field within the framework that points from the framework to the fluorinated sites. This deep electronic structure regulation makes specific carbon atoms in the framework electron-deficient active centers, greatly optimizing their adsorption and activation ability for oxygen molecules, and significantly enhancing ORR performance [80].

Complementary to the electron-withdrawing strategy, introducing electron-rich units to achieve electron-donating polarization is also an effective electronic regulation method. A study demonstrated how electron-rich units can polarize the framework and enhance performance by constructing a three-dimensional fully conjugated COF enriched with thiophene. Thiophene, a typical electron-rich five-membered heterocycle, acts as a strong electron donor when integrated into the conjugated framework, contributing electron density to the entire *π* system and creating an electron density gradient between the electron-rich thiophene regions and the rest of the framework. This electron-donating polarization creates a microenvironment favorable for charge transfer, optimizing the local electronic states of potential active sites and achieving efficient metal-free ORR catalysis [46]. Further, integrating the electron-rich carrier piperazine into the COF framework upgrades this strategy. Piperazine not only acts as a strong electron donor to produce a significant static polarization effect, thereby increasing the electron density of the framework, but also dynamically stores and releases electrons during the catalytic process, providing immediate electron supply to the active sites. Additionally, it can induce keto-enol tautomerism, allowing reversible conversion between hydroxyl and carbonyl groups, thereby significantly enhancing the polarity of the COFs [81]. The synergy of static and dynamic polarization effects endows it with outstanding ORR catalytic performance.

Beyond the single-polarity regulation, integrating donor and acceptor units through covalent bonds in a spatially ordered manner to construct donor–acceptor heterojunctions is an advanced strategy for achieving macroscopic-scale charge separation and polarization. In one design, electron-rich cobalt porphyrin serves as the electron donor, while the strongly electron-withdrawing BTZ unit acts as the electron acceptor. Both spectroscopic characterization and theoretical calculations confirm significant electron transfer from the porphyrin unit to the BTZ unit, creating a highly polarized environment throughout the framework. This polarization effect optimizes the adsorption energy of the OOH* intermediate at the Co active center, bringing it closer to the theoretical peak of the volcano plot, thereby endowing the material with exceptional ORR activity and four-electron selectivity. This work clearly demonstrates the great potential of D-A type COFs in electronic structure regulation [82]. The D-A connection strategy has been successfully applied in other energy systems, for instance, Zhao et al. [83] constructed D-A connected COF electrolytes to regulate the electron density in solid-state batteries, which confirmed the wide applicability of this electron regulation method. Meanwhile, another study synthesized two CoPc-D-A-COFs, which visually demonstrated the D-A structure in Fig. 7c [35]. The advantages of D-A engineering are also reflected in the precise control of selectivity. For instance, by fine-tuning the pyridine nitrogen units in vinyl-linked COFs, the electron-withdrawing ability of the linkers was systematically altered, precisely regulating the strength of D-A interactions and the degree of framework polarization, ultimately achieving directional control over the selectivity for the 2e^−^ ORR pathway to generate H_2_O_2_ [84].

The polarization strategy is still expanding. On the one hand, research is moving from static design to understanding dynamic polarization processes. By systematically introducing nitrogen-containing units with different electronic properties into the COF, from neutral pyridine to ionized pyridine and then to ionized imidazole units, a continuous regulation of a series of key physicochemical properties of the material has been achieved, including dipole moment, reduction ability, hydrophilicity and hydrophobicity, and most importantly, the binding energy of reaction intermediates. It is worth noting that the ionized imidazole COF (im-PY-BPY-COF) stands out particularly, with its activity significantly surpassing that of neutral COFs and ionized pyridine COFs. It exhibits an outstanding half-wave potential of 0.80 V in 0.1 M KOH, ranking among the top values reported for metal-free COFs at that time [85]. Apart from the specific strategies targeting the COF, recent advancements in spin-related catalysis have also provided new inspirations for research: In systems with bipolar iron–cobalt magnetic atoms, spin balance is crucial for efficient acidic water oxidation, indicating that spin-state engineering may become a future development direction for catalysts based on covalent frameworks [86]. On the other hand, future research will place greater emphasis on systematicity, that is, by systematically designing a series of COF-based electrocatalyst with different core polarities, a model from molecular structure to polarization intensity and then to catalytic performance will be established, thereby achieving the customizability of catalytic functions. Researchers have successfully constructed a COF system with continuously varying polarities by selecting C3-symmetric building units with different polarities, precisely regulating the charge density of the active sites (Fig. 7b). Notably, contrary to intuition, the COF with a lower-polarity benzene core demonstrated the best performance in H_2_O_2_ synthesis, with a selectivity of up to 93.1%, a Faraday efficiency of 90.5%, and an electron transfer number close to the theoretical value of 2 [87]. This discovery challenges the traditional notion that stronger polarity leads to better catalytic performance, revealing a complex nonlinear relationship between polarization intensity and catalytic selectivity.

#### Constructing Bifunctional COFs

After systematically discussing the enhancement of single-function catalytic performance through metal anchoring strategies in the previous part, the research naturally extended to how to integrate these active sites into a single material to achieve dual-function catalysis for ORR and OER. This integration is crucial for the development of advanced energy devices such as rechargeable zinc–air batteries. In recent years, through reasonable molecular design and structural regulation, researchers have developed various efficient bifunction COF-based electrocatalyst. The design strategies mainly focus on the rational design of active sites, structural optimization, and composite system construction.

**Fig. 8:**
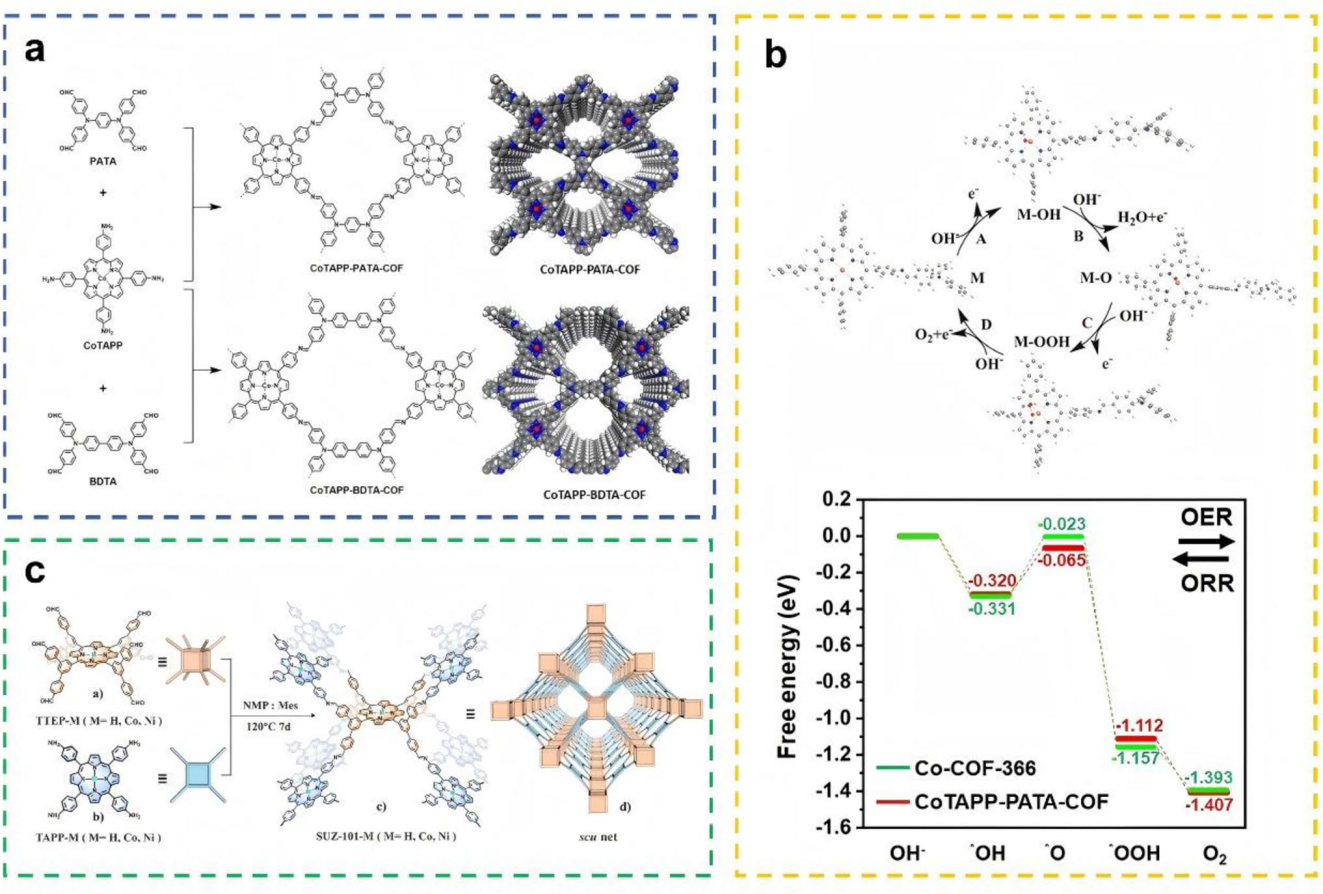
**a** Synthesis of catalytic COFs (CoTAPP-PATA-COF and CoTAPP-BDTA-COF) from CoTAPP, PATA and BDTA, respectively. **b** Free energy diagram for the bifunctional CoTAPP-PATA-COF (ORR/OER) [63]. Copyright 2022, Wiley. **c** Molecular structures of the two structural monomers TTEP-M and TAPP-M, as well as the extended framework and Scu topological structure of the three-dimensional COFs material SUZ-101-M synthesized by the condensation of these two structures [18]. Copyright 2025, Royal Society of Chemistry

The construction of dual-function catalysts begins with the precise design of active sites. A pioneering work successfully integrated a strong electron donor, a diarylamine derivative, with the redox-active center into a cobalt porphyrin-based framework, constructing an intrinsic dual-function COF catalyst (CoTAPP-PATA-COF and CoTAPP-BDTA-COF) with a clear crystal structure (Fig. 8a) [63]. This study achieved precise control over the local microenvironment of the active sites through carefully designed molecular structures. The diarylamine unit not only significantly promoted electron transfer within the conjugated backbone but also greatly enhanced the electrochemical active surface area of the material due to its inherent redox activity. Systematic electrochemical tests demonstrated that CoTAPP-PATA-COF exhibited excellent dual-function catalytic performance in 0.1 M KOH electrolyte, with an ORR half-wave potential reaching 0.80 V and an OER overpotential of 420 mV. Through in-depth mechanism studies, the authors found that the introduction of the diarylamine unit optimized the electronic structure of the cobalt active center and reduced the adsorption energy barrier for oxygen intermediates (Fig. 8b). This study proved that through precise molecular engineering, efficient dual-function catalysis can be achieved without sacrificing the inherent structural advantages of COF. Another study achieved precise control of the electronic structure of active sites by constructing a fully conjugated COF with a D-*π*-A structure [88]. As described in Sect. 3.1.2, the construction of donor–acceptor (D-A) structures can generate a strong polarization effect. Here, we further extend this concept to a D-*π*-A structure combined with three-dimensional graphene, which not only enables framework polarization but also promotes the dual-function oxygen reduction reaction/oxygen evolution reaction. After being combined with three-dimensional graphene, the ORR half-wave potential of this material reached 0.880 V, with a total overpotential of only 0.617 V, demonstrating the powerful power of molecular-level electronic structure design.

To overcome the problem of low utilization of active sites in two-dimensional materials, a three-dimensional COF-based electrocatalyst constructed from two carefully designed metal porphyrin monomers exhibited outstanding performance in SUZ-101-Co (Fig. 8c) [18]. This material demonstrated excellent comprehensive performance: an OER overpotential of only 240 mV at 10 mA cm^−2^ current density and an ORR half-wave potential of 0.78 V. In-depth structural characterization indicated that the three-dimensional topological structure created a continuous porous channel system, effectively avoiding the interlayer stacking problem of two-dimensional materials, and increasing the density of active sites by nearly three times compared to two-dimensional analogues. Theoretical simulations further confirmed that this three-dimensional open structure not only improved the accessibility of active sites but also optimized the mass transfer process of reactants. Notably, this material maintained good structural stability during long-term cycling tests, demonstrating that the three-dimensional COF can effectively alleviate structural stress in the catalytic process.

While the development of metal-based catalysts has been ongoing, significant breakthroughs have also been achieved in the field of non-metal catalyst systems. Pyrrole-based bifunctional COF (NUST-38) achieves excellent bifunctional catalytic performance through precise local microenvironment regulation [89]. This study systematically explores the influence of local microenvironment factors such as heteroatom doping, methyl introduction, and linker length on electrocatalytic activity. The optimized NUST-38 exhibits excellent catalytic performance in 1 M KOH: at a current density of 10 mA cm^−2^, the overpotential for HER is only 182 mV, and the overpotential for OER is 303 mV. More importantly, this material maintains its catalytic performance after 10,000 CV cycles, demonstrating its excellent structural stability. Through in-depth characterization and theoretical calculations, researchers found that the abundant nitrogen species in the pyrrole unit create efficient catalytic active centers, while the precisely controlled pore structure optimizes the mass transfer process of reactants.

To address the issue of insufficient conductivity of COF, by in situ growing COF layers on the surface of highly conductive carbon nanotubes and coordinating with Co(II), researchers successfully constructed the Co-CNT@COF-Pyr composite catalyst [90]. The core advantage of this design lies in achieving perfect synergy among the components, where carbon nanotubes provide a fast electron transmission channel, the COF layer ensures the high dispersion and controllability of the active sites, and the coordination of Co(II) with the pyridine unit creates a highly active catalytic center. Electrochemical tests show that this composite material exhibits excellent bifunctional characteristics in alkaline electrolyte: for OER, at a current density of 10 mA cm^−2^, the overpotential is 438 mV. For ORR, in the range of 0.1–0.6 V vs. RHE test potential, the hydrogen peroxide yield remains at a low level of 9.0%-10.1%, and the reaction path is close to the 4-electron process (electron transfer number *n* = 3.82–3.80). This precise interface engineering design not only solves the problem of insufficient conductivity of COF-based electrocatalysts but also significantly enhances catalytic performance through the synergy of component interactions. More groundbreaking is the research on COF@MOF core–shell structure-derived materials. Using TP-BPY-COF@ZIF-67 as the precursor, it was successfully synthesized after thermal decomposition, containing uniform Co nanoparticles and atomically dispersed Co-N_4_ sites in the core–shell carbon framework [91]. This unique structure enables the material to exhibit excellent activity in both ORR and HER, highlighting the potential of precise precursor design to achieve functional integration. The research on derivative materials based on COF precursors has opened up new perspectives for optimizing catalyst performance. The Fe, Co–N–C catalyst prepared from Fe, Co bimetallic COF through precise control of the pyrolysis process retains atomic-level dispersed Fe–N_4_ and Co–N_4_ sites while achieving high conductivity and stability of the carbon material [92], Similar to the bimetallic synergy discussed in Sect. 3.1.1 (e.g., CuPcF8-CoPc-COF), the bimetallic Fe, Co–N–C catalyst obtained from COF also benefits from the electron coupling between adjacent metal centers. The detailed mechanism can be found in this section. This material exhibits excellent bifunctional performance under alkaline conditions, with an ORR half-wave potential of 0.85 V and an OER overpotential of 330 mV. The characterization results show that the derived materials inherit the ordered pore structure of the precursor and form a highly graphitized carbon matrix, ensuring good conductivity and stability of the material. It is also worth noting that the fluorine–nitrogen co-doped porous carbon catalyst prepared by direct pyrolysis of fluorine and nitrogen-containing COF precursors demonstrates the powerful effect of hybrid atomic engineering [52]. The introduction of fluorine not only effectively promotes the formation of edge defects, but also the strong synergistic effect with nitrogen atoms provides the catalyst with high intrinsic activity. The zinc–air battery based on this catalyst shows a peak power density of up to 206.3 mW cm^−2^, surpassing the performance of the commercial Pt/C + RuO_2_ catalyst. Similarly, the Fe, Co–N–C catalyst prepared from Fe, Co bimetallic COF retains the dispersed active sites while achieving high conductivity of the carbon material [92].

The rational design guided by theoretical calculations provides a new model for the development of bifunctional COF catalysts. Starting from the experimentally synthesized (phen_2_N_2_)FeCl molecule, researchers used constant potential first-principles calculations to theoretically predict a stable two-dimensional organic metal framework—(phen_2_N_2_)FeCl monolayer [93]. The uniqueness of this structure lies in its pyridine-type FeN_4_ coordination environment, which contrasts sharply with most two-dimensional organic metal frameworks with pyrrole coordination. Detailed electronic structure analysis indicates that this special coordination environment enables the material to achieve a high single-atom Fe loading in heterogeneous systems. Constant potential energy analysis and microkinetic models prove that this monolayer has great potential to promote bifunctional ORR/OER in both acidic and alkaline conditions, with theoretical activity higher than traditional Fe–N–C materials, (phen_2_N_2_)FeCl molecules, and noble metal Pt/IrO_2_ benchmark catalysts. More importantly, in the series of (phen_2_N_2_)MCl single-layer materials studied in the research, where M is Mn, Co, or Ni, the (phen_2_N_2_)MnCl single-layer was also confirmed to have excellent bifunctional activity.

In-depth studies of the catalytic mechanism provide a solid theoretical basis for catalyst design. A comprehensive study of fluorine–nitrogen co-doped porous carbon revealed the microscopic mechanism of hybrid atomic catalysis [94]. Researchers examined all possible N and F co-doping methods and conducted detailed theoretical analysis of seventeen optimized carbon cluster structures. The calculation results show that the co-doping of F with graphite nitrogen and pyridine nitrogen can trigger active paramagnetic centers, and the co-doping of covalent F with graphite nitrogen is particularly able to provide the lowest free energy barriers for ORR and OER. This theoretical prediction has been fully supported by the experimental results. The prepared NFPC catalyst demonstrated outstanding catalytic activity in zinc–air batteries, outperforming many other catalysts. Looking to the future, emerging concepts in adjacent fields may provide inspiration for the design of the next generation of COF. For instance, Liu et al. [95] recently achieved bipolar doping in a Janus catalyst with a bimetallic structure to realize a light-enhanced rechargeable zinc–air battery. This indicates that integrating light-responsive components into bimodal catalysts based on COF can further enhance performance.

The development of bifunctional COF catalysts shows a clear trend. It progresses from the design of basic active sites to the optimization of three-dimensional structures, from single metals to dual-metal synergy, from metal-based to non-metal-based systems, and further advances through the study of composite and derivative materials for application. Ultimately, it achieves rational design under the guidance of theoretical calculations. This development trend is similar to that of the development process of metal-based COF and non-metal-based COF described in the second chapter, but with different focuses. This subsection combines the contents of both chapters and discusses its contribution in constructing bifunctional COF-based electrocatalyst.

#### Supramolecular Engineering

Supramolecular engineering is a burgeoning strategy for constructing functional materials by precisely regulating non-covalent interactions between molecules, including hydrogen bonds, *π*-*π* stacking, and van der Waals forces. The core value of this strategy lies in its ability to go beyond traditional covalent bond design, and optimize charge transport in the material, active site exposure, and quality transfer efficiency from a higher dimensional perspective by manipulating molecular packing patterns, interface behaviors, and pore microenvironments.

**Fig. 9:**
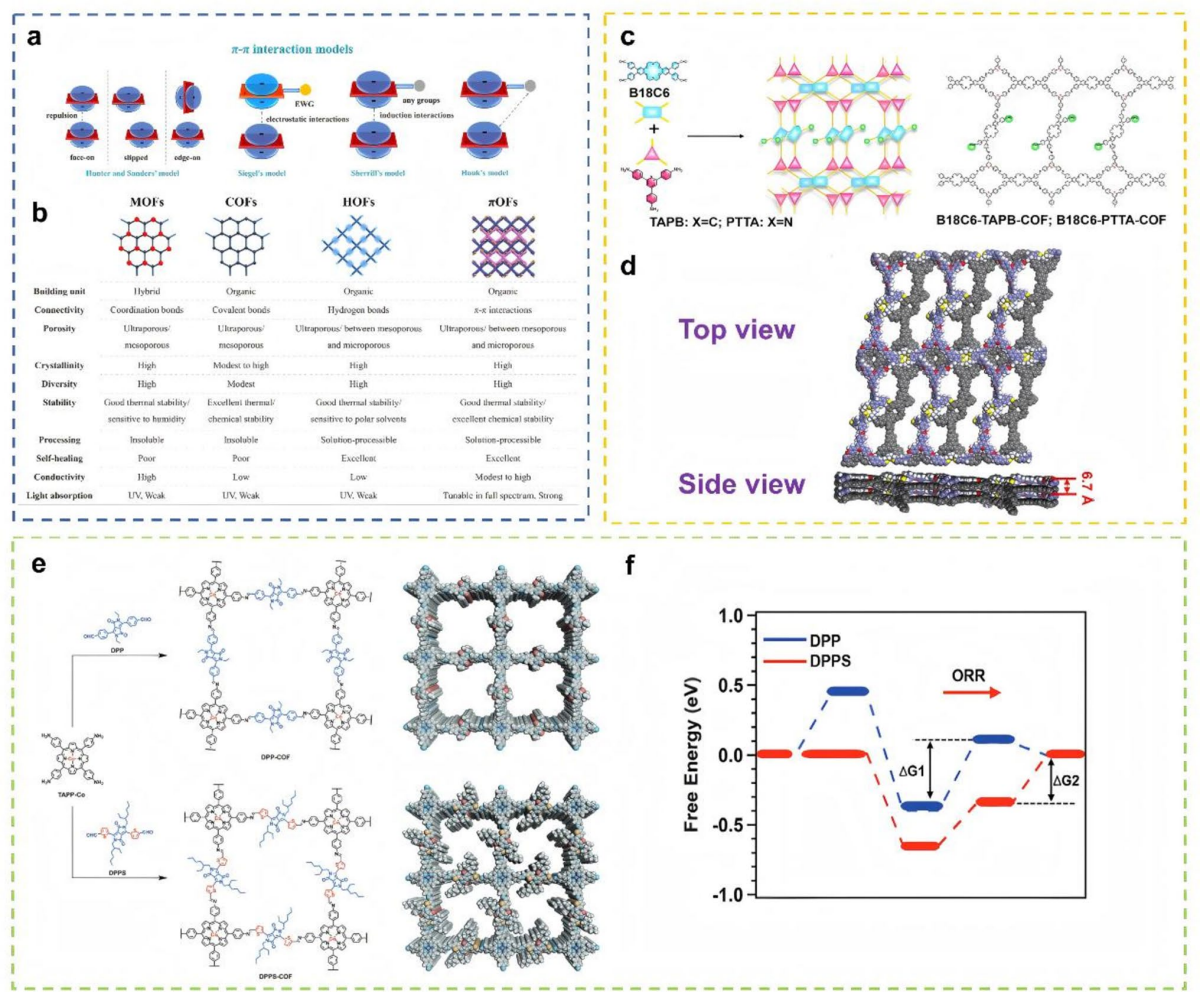
**a** Schemes for describing p–p interaction models. **b** Comparison of *π*OFs with MOFs, COFs, and HOFs [96]. Copyright 2024, Elsevier. **c** Synthetic route of B18C6‐TAPB‐COF and B18C6‐PTTA‐COF. **d** Top view and side view of the c‐AB stacking structure of B18C6‐PTTA‐COF based on PXRD and modeling [97]. Copyright 2024, Wiley. **e** Synthesis routes and top-down structural diagrams of DPP-COF and DPPS-COF. **f** Free energy diagram for the effect of side-chain length on ORR (DPPS-COF) [98]. Copyright 2024, Wiley

In-depth research on pure supramolecular frameworks provides important theoretical support for supramolecular engineering in COFs. Non-covalent organic frameworks (*π*OFs) constructed entirely through *π*-*π* stacking exhibit the essential characteristics of supramolecular engineering. These materials self-assemble into crystalline porous networks through *π*-*π* interactions between molecules (Fig. 9a), demonstrating excellent solution processability, dynamic self-repair capabilities, and outstanding carrier mobility (Fig. 9b) [96]. These properties are attributed to the flexibility, reversibility, and conductive nature of *π*-*π* interactions, providing important theoretical guidance and inspiration for implementing various supramolecular engineering strategies in COFs and offering new ideas for addressing the bottleneck issues of poor processing and insufficient mechanical properties of traditional COF-based electrocatalyst.

In the specific implementation strategies of supramolecular engineering, stacking control demonstrates strong regulatory capabilities. By carefully designing COF structures with pendant aldehyde groups and inducing their formation of interlaced stacking patterns (Fig. 9c), researchers successfully achieved high selectivity control of the 2e^−^ oxygen reduction pathway. In this work, the unreacted pendant aldehyde groups extend beyond the COF plane, making them more accessible in the electrolyte than the framework sites. More importantly, these aldehyde groups adopt an interlaced stacking pattern, providing a larger mass transfer space compared to a stacked pattern (Fig. 9d). By adjusting the tridentate linkers in the COF, catalytic performance was effectively regulated, and the optimal COF exhibited 91% H_2_O_2_ selectivity and 12.2 A g^−1^ quality activity [97].

Theoretical calculations further revealed that the B18C6-PTTA-COF containing pyridine has higher activity due to its ability to promote the binding of the intermediate OOH* on the pendant aldehyde carbon. These studies prove that supramolecular engineering not only optimizes the mass transfer path but also regulates reaction selectivity by precisely controlling the spatial orientation of active sites. Based on this, the researchers found that simple side-chain functionalization can simultaneously achieve interlayer space expansion and electronic structure regulation. This study, through the functionalization of long chains with electron-donating properties, not only expanded the interlayer distance but also reduced the band gap (Fig. 9e). Although the specific surface area is less than 1/10, the obtained DPPS-COF shows a higher electrochemical surface area, providing more exposed active sites [98]. DPPS-COF exhibits excellent electrocatalytic ORR activity, with a half-wave potential of 0.85 V, which is 30 and 60 mV more positive than Pt/C and DPP-COF, respectively, setting a record among the reported COFs. Theoretical calculations further reveal that the longer electron-donating chains not only promote the formation of O_2_ intermediates OOH*, but also facilitate the desorption of intermediates, thereby achieving higher activity (Fig. 9f).

From the control of two-dimensional stacking to the regulation of material dimensions, supramolecular assembly demonstrates a broader design space. The construction of one-dimensional COFs breaks through the limitations of traditional two-dimensional materials and solves the problem of the catalytic center being located at the base layer and difficult to be contacted by the electrolyte. COFs prepared using different four-link blocks show good crystallinity, high specific surface area, and excellent chemical stability. The more exposed catalytic sites enable 1D COF to exhibit a large electrochemical active surface area, which is 4.8 t that of the 2D COF. Thus, it can catalyze the ORR with 85.8% H_2_O_2_ selectivity and a TOF value of 0.051 s^−1^, which is higher than the ORR performance of 2D COF. The H_2_O_2_ selectivity and TOF value of 2D COF are 72.9% and 0.032 s^−1^, respectively [99].

Extending the supramolecular assembly strategy from a single material to multiple components has given rise to an innovative breakthrough in heterogeneous interface engineering. The one-dimensional van der Waals heterojunction is achieved through the face-to-face assembly of the carbon nanotube core and the thickness-tunable thienothiophene–pyrene COF shell layer via van der Waals forces. Density functional theory calculations and operando spectroscopy analysis indicate that the carbon–sulfur regions of the thienothiophene group in the COF serve as active catalytic sites for oxygen reduction and release reactions. The coaxial structure realizes n-doping from the CNT core to the COF shell layer, which can be controlled by changing the thickness of the COF shell layer. The charge transfer from the CNT reduces the band gap and work function of the COF, reducing the charge transfer barrier between the active catalytic sites and the adsorbed oxygen intermediates, thereby significantly enhancing the catalytic activity.

More in-depth research has revealed that in the hybrid system of vinyl-connected COF and ruthenium oxide [100], the formation of a controllable functional adsorption layer at the catalyst–water interface is a powerful strategy to accelerate proton transfer and deprotonization. In situ spectroscopy measurements combined with theoretical calculations indicate that the hydrogen bonds assembled between the COF and the adsorbed oxygen intermediates effectively direct the interface water molecules, stabilizing the transition state of the OER intermediate formation. This determines the reduction of the proton transfer and deprotonization energy barriers, thereby generating excellent acidic OER performance. When integrated into a PEMWE device, this system achieved a record current density of 1.0 A cm^−2^ at a battery voltage of only 1.54 V, and has long-term stability exceeding 180 h at an industrial-level 200 mA cm^−2^.

These supramolecular heterostructures based on COF demonstrate great potential for system integration. Recent reviews have systematically summarized the progress in this field, indicating that by supermolecular integration of COF with conductive components such as metal oxides, metal nanoparticles, and carbon-based materials, the resulting synergistic effects can improve charge transfer, enhance catalytic stability, and promote reaction kinetics [101].

#### Modulating Edge Sites

As mentioned earlier, through strategies such as metal anchoring, induced polarization, and the construction of dual-functional structures, the intrinsic activity and synergy of the active sites within the COF main framework can be effectively optimized. However, the catalytic performance not only depends on the quality of the sites but is also closely related to their quantity and accessibility. Based on this, the research perspective has expanded beyond surface modification to encompass the field of material boundary control, namely the precise regulation of edge sites. Edge sites, due to their inherent unsaturated coordination state and highly adjustable electronic structure, often exhibit a reaction activity far higher than that of the basal plane sites. Modulating edge sites aims to precisely manipulate the chemical composition, spatial position, and electronic structure of the edge region through rational molecular-level design, thereby further maximizing their quantity and achieving the specificity of their functions, in order to further enhance the catalytic performance of COF.

**Fig. 10:**
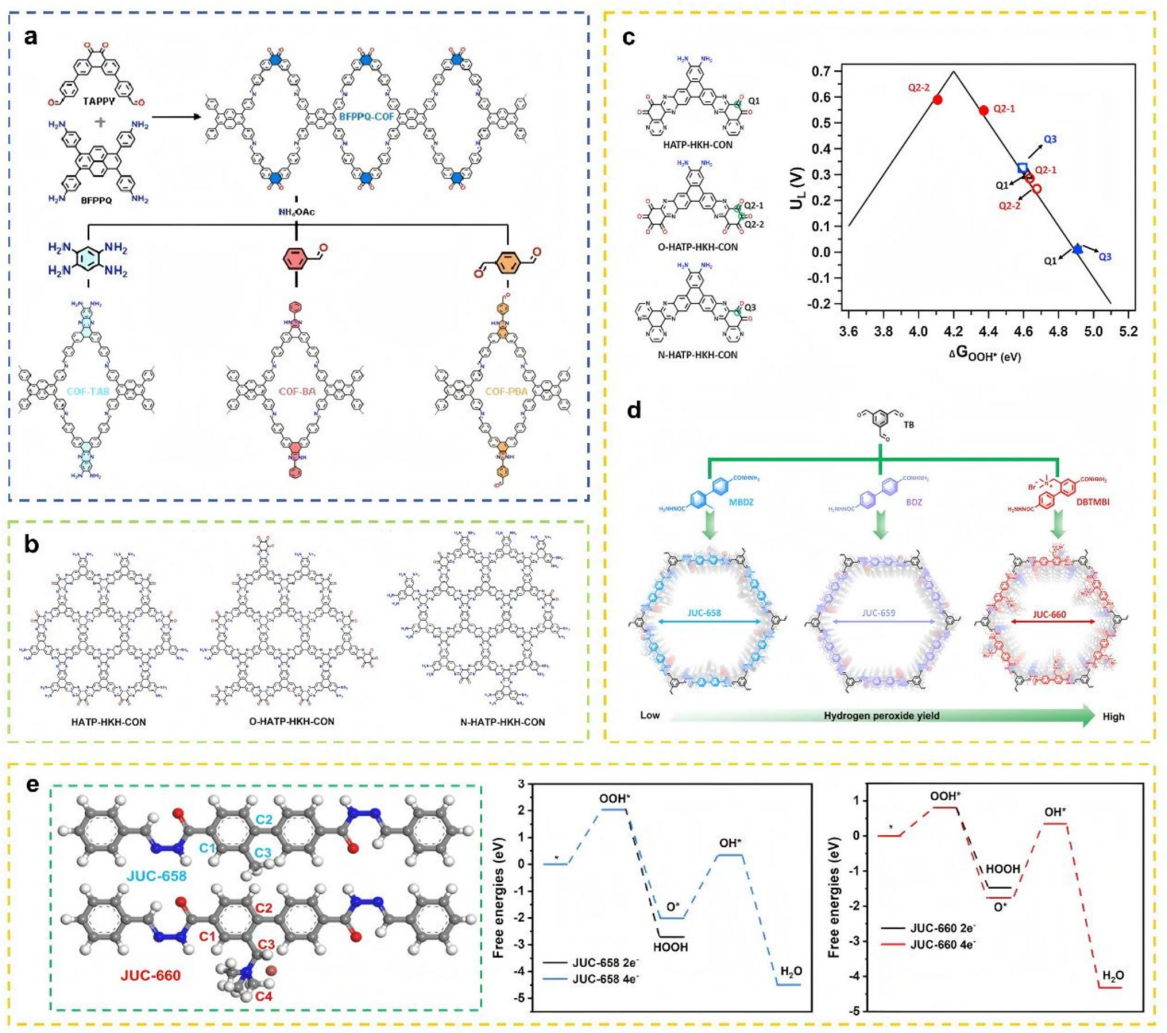
**a** Mechanism of reaction of different active sites by phenanthrenequinone and chemical structure Of BFPPO.COF, COF.TAB, COF-BA, and COF-PDA [102]. Copyright 2024, Wiley. **b** Three catalytic CON structures for generating hydrogen peroxide, constructed through edge site engineering: HATP-HKH-CON, O-HATP-HKH-CON and N-HATP-HKH-CON. **c** Theoretical analysis of conjugated systems with different end-group positions, including the different functional group configurations under investigation, as well as the 2e^−^ oxygen reduction limit potential volcano diagram calculated as a function of ΔGOOH* [105]. Copyright 2021, Elsevier. **d** Synthesis and structures of JUC-658, JUC-659, and JUC-660. **e** Free energy diagram comparing 2e^−^ vs. 4e^−^ pathways for edge-modified JUC-660 [104]. Copyright 2024, Wiley

The regulation of the chemical composition of edge sites is the basis for optimizing their performance. Researchers have thus constructed an ideal one-dimensional COF research platform. The ingenious aspect of this work lies in introducing different functional groups such as carbonyl, diaminopyrazine, and phenylimidazole systematically at the edge while maintaining the consistency of the framework and pore structure (Fig. 10a). Through this controlled variable design, the researchers directly attributed the performance differences to the different electronic states of carbon at the edge sites [102]. The results showed that the phenylimidazole edge functional group could most effectively optimize the adsorption of the OOH* intermediate, thus demonstrating the optimal ORR activity. This reveals the feasibility of optimizing the oxygen reduction reaction activity through molecular-level edge site regulation.

After clarifying the importance of the spatial layout of the edge sites, it becomes a key factor affecting the catalytic efficiency. By precisely anchoring the same single-atom Pt sites on the inner wall and outer surface of the one-dimensional COF pore, it was convincingly demonstrated that the spatial position of the sites has a decisive effect on the ORR kinetics. The study found that the Pt sites located inside the pore exhibited a much higher intrinsic kinetic activity than those on the outer surface [103]. Exploring the reason, it was found that the mechanism lies in the higher efficiency of potential-induced anion transport within the nanochannels, and the local reconstruction of Pt–Cl bond breakage also occurs more effectively within the channels, which promotes the dynamic reconstruction of the active sites and is more conducive to the desorption of key reaction intermediates. This discovery clarifies the core design principle of prioritizing the anchoring of active edge sites within the pore.

Fine regulation of the electronic structure of edge sites at the atomic level is an advanced strategy for achieving high selectivity catalysis. The JUC-660 COF reported by the researchers proves this point [104]. This work deeply modified the electronic cloud density of the active center by charging the edge benzyl units (Fig. 10d), significantly weakening the adsorption of the OOH* intermediate and successfully precisely redirecting the ORR reaction path from 4e^−^ transfer to 2e^−^ transfer (Fig. 10e), achieving a continuous H_2_O_2_ yield of over 1200 mmol g^−1^ h^−1^.

In addition to modifying the edge sites on the preset framework, directly constructing catalytically active edge defects is also a powerful strategy. A study has demonstrated the feasibility of this edge defect engineering. This study successfully created density and position-controllable active sites by deliberately constructing edge defects such as suspended carbonyl units in the covalent organic network (Fig. 10b), achieving over 99% selectivity in the 2e^−^ ORR [105]. Theoretical calculations indicate that the carbon atoms adjacent to the carbonyl group are the true active centers, and their unique electronic structure enhances the binding ability to OOH*. This demonstrates the great potential of creating novel highly active sites by designing imperfect edges (Fig. 10c). Feng et al. [106] further emphasized the significance of defect engineering in COFs. They developed defect-engineered COF nanofibers with superelastic properties for the extraction of uranium, indicating that controllable defects can simultaneously enhance mechanical flexibility and the exposure of active sites.

#### Building Catalytic Linkage

The catalytic performance is not only determined by the nature of the active center but is also profoundly influenced by its chemical environment. Building catalytic linkage precisely regulates the electronic structure, spatial configuration, and mass transfer behavior of the material at the molecular level through rational design of the chemical connection bonds that constitute the framework, thereby achieving precise optimization of the catalytic performance. Unlike the inducting polarization strategy (Sect. 3.1.2), the building catalytic linkage mainly alters the electronic cloud of the framework through heteroatoms or D-A units, while directly constructing catalytic linkages directly designs the connecting bonds. This approach can be regarded as a complementary tool at the molecular level to achieve similar electronic effects, as demonstrated below. This strategy forms an effective complement to the aforementioned methods and together constitutes a complete system for the design of COF catalysts. It is worth noting that similar atomic-level optimizations have been proved to be effective in related porous materials, for instance, He et al. [107] demonstrated that optimizing bond lengths in metal–organic frameworks can significantly accelerate the rate of oxygen electrocatalytic reactions, which further highlights the importance of structural design.

**Fig. 11:**
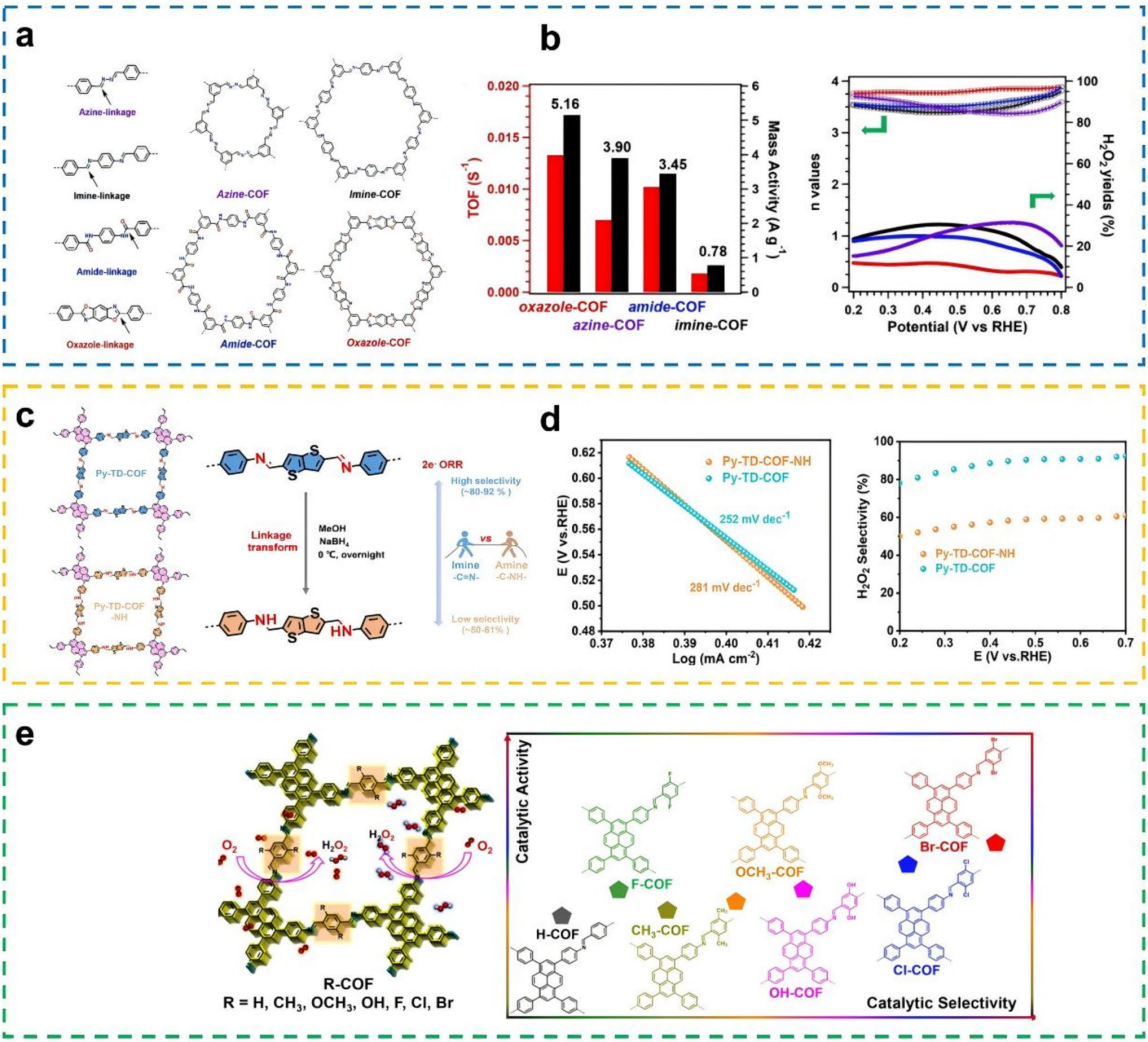
**a C**hemical structure of four catalytic linkages (azine, imine, amide, and imidazole) used for catalyzing redox reactions and their corresponding COFs compounds. **b** Free energy diagram for different linkage bonds (oxazole vs imine) [108]. Copyright 2023, Wiley. **c** Chemical structures of Py-TD-COF, Py-TD-COF-NH, and the schematic illustration of their electrocatalytic 2e^−^ ORR. **d** Tafel plot and the H_2_O_2_ selectivity of Py-TD-COF-NH and Py-TD-COF, Tafel slopes obtained from linear sweep voltammetry (LSV) at 5 mV s^−1^ in O_2_-saturated 0.1 M KOH [110]. Copyright 2024, Elsevier. **e** Constructing COFs for 2e^−^ ORR via linker engineering [114]. Copyright 2024, Elsevier

The research on building catalytic linkage begins with the screening of basic connection bonds. To verify the crucial role of connection bonds in catalytic reactions, researchers have provided pioneering exploration. One work innovatively used benzene rings as the sole building unit to construct COFs with four different connection bonds: imine, amide, pyrazine, and oxazole (Fig. 11a) [108]. Under the condition of excluding the interference of other active sites, it clearly revealed the decisive role of the connection bonds themselves in catalytic performance. Among them, the COF with oxazole connection exhibited excellent ORR activity, with a half-wave potential of 0.75 V, and the conversion frequency was 1.9, 1.3, and 7.4 t that of COFs with other connection bonds (Fig. 11b). In-depth mechanism studies demonstrated that the carbon atoms in the oxazole bond could effectively promote the formation of OOH* intermediates and accelerate the protonation process of O* ions. This discovery provided a profound understanding at the molecular level of the catalytic function of the connection bonds. Recently, Chen et al. [109] further expanded this concept. They demonstrated the application of electrochemical carbon dioxide reduction by implementing single-point connection engineering in conjugated porphyrin-based covalent organic framework materials. The results showed that making minor modifications at the connection points could significantly alter the catalytic selectivity and activity. Based on this, researchers began to focus on the precise regulation of reaction pathways by the connection bonds. By comparing Py-TD-COF with pyridine-based COFs with amide and amine connections (Fig. 11c) [110], it was found that although using the same building unit, the Py-TD-COF with imine connection achieved 80%-92% H_2_O_2_ selectivity, while the Py-TD-COF-NH with amine connection had a selectivity of only 50%-61% (Fig. 11d). Further mechanism studies revealed that the hydrogen bond formed between the amine group and the adsorbed oxygen molecule in the amine connection elongated the O–O bond, which not only accelerated the subsequent hydrogenation process but also significantly reduced the energy barrier for the reduction of OH to O, thereby promoting the 4e^−^ reaction pathway.

As the research progressed, the development of new connection bonds has not only solved the stability problems of traditional materials but also provided a new platform for the practical application of the induced polarization strategy. Researchers innovatively used non-polar C = C connection bonds to construct a COF framework centered on pyridine [111]. This design fundamentally avoided the decomposition problem caused by polar connection bonds in catalytic processes, significantly enhancing the intrinsic stability of the material. More importantly, researchers successfully implemented induced polarization on this non-polar framework by introducing positive charges on the nitrogen atom of pyridine, achieving precise control of the electronic distribution around the active site, thereby greatly promoting the transfer of electrons to the catalytic center. Ultimately, this strategy of combining stable non-polar connections with precise charge polarization enabled the new material to maintain excellent stability while achieving a half-wave potential of 0.74 V and a mass activity of 4.34 A g^−1^, which was 50 mV and 1.63 t higher than that of neutral COFs.

In terms of topological innovation, the trapezoidal topological structure demonstrates significant advantages due to its unique structural stability and electron delocalization characteristics. The researchers pioneered the use of natural phenolic molecule tannic acid as a linking unit to construct CoPc-EA-COF trapezoidal COF [112]. This design based on natural products not only simplifies the material preparation process but also significantly enhances the electronic conductivity and CO_2_ binding ability of the material. This material exhibits a high half-wave potential of 0.80 V in ORR and achieves a CO Faradaic efficiency of 97.32% and a conversion frequency of 2092 h^−1^ in CO_2_RR, demonstrating excellent bifunctional catalytic performance and providing new ideas for the development of environmentally friendly catalytic materials. Another study further developed metal phthalocyanine trapezoidal COF [113]. The researchers constructed iron phthalocyanine-based trapezoidal COF through a benzimidazole–benzophenanthroline linking unit. Thanks to its stable trapezoidal topological network, it achieved an outstanding half-wave potential of 0.933 V in ORR and demonstrated practical application value in zinc–air batteries.

Furthermore, the fine molecular-level control has pushed the strategies for building catalytic linkage toward more precise design. The introduction of substituent strategies shows great potential. The researchers systematically studied the influence of physical and chemical properties such as crystallinity, porosity, and dipole moment of COF on the 2e^−^ ORR performance by introducing different substituents on the linking groups. The bromine-substituted Br-COF achieved 86.2% H_2_O_2_ selectivity and 32.0 A g^−1^ mass activity after optimization, which were 112% and 174% higher than that of unmodified COF, respectively (Fig. 11e) [114]. In-depth mechanism studies revealed that the reduction ability of the material is the most critical factor affecting catalytic activity, and the easy formation of OOH* intermediates is the key to achieving high activity. Meanwhile, to optimize the reaction microenvironment, in the study of side chains, Researchers optimized the hydrophilic nanochannels of this material by adjusting the length of the polyether side chains, achieving a H_2_O_2_ yield of 5820 mmol g^−1^ h^−1^, fully demonstrating the fine influence of side-chain length regulation on catalytic performance. At the geometric control level, the researchers adopted the thienyl position isomerization strategy to construct two isomers, COF-α with 2-position substitution and COF-β with 3-position substitution [115], successfully regulating the electronic state of the material through geometric modification, achieving an ORR half-wave potential of 0.76 V, superior to most non-metal or metal-based electrocatalysts. Through combined analysis of theoretical predictions and in situ Raman spectroscopy, the study accurately identified the five-membered ring carbon adjacent to the sulfur atom as the active center, demonstrating the precise control ability of geometric control on the formation of active sites.

Like other strategies in Chapter 3, theory-driven rational design represents the future development direction of building catalytic linkage. Through theoretical calculations guided by theory, the researchers successfully designed and synthesized a new two-dimensional COF-C_4_N with a phenazine linking group, which exhibited a low overpotential of 349 mV in OER [53]. A review systematically elaborated the basic theory of connection chemistry, providing a complete theoretical framework for various building catalytic linkage strategies [116]. This review profoundly expounded the fundamental position of connection chemistry in COF catalytic materials, providing a complete theoretical framework for understanding various building catalytic linkage strategies. The connection bond not only determines the structural orderliness and physical and chemical properties of the material, but also profoundly affects the catalytic performance by regulating key parameters such as specific surface area, crystallinity, chemical stability, and photoelectronic behavior through regulation.

The six structural design strategies discussed in Sects. 3.1.1–3.1.6 are compared in Table 4 based on their core mechanisms, main advantages, and limitations. Moreover, to illustrate the effectiveness of each structural design strategy, we summarize the representative performance improvements (such as overpotential reduction, half-wave potential, hydrogen peroxide selectivity, etc.) in Table 5 according to the original literature.

**Table 4:** Summary of six structural design strategies for COF-based electrocatalysts (mechanisms, advantages, limitations)

Strategy	Core mechanism	Key advantage	Representative performance improvement	References
Metal anchoring	Fix the coordinating metal atoms/nanoparticles at the specific vacancies of the COF structure	The active site is clearly defined, allowing for the design of single-atom/dual-metal/composite structures	The direct impact on the electronic structure and stability of ORR is a decrease of 26 mV in the overpotential, and an increase of 3.2 t in the activity, the overpotential of bimetallic ORR decreases by 40 mV, the ΔE of zinc–air battery is 0.78 V	[71–77]
Inducing polarization	Fixing coordination-attached metal atoms/nanoparticles in COF at specific vacancy heteroatoms, D-A structures, electron donors/receivers, fluorination, etc	The active site is clearly defined, allowing for the design of single-atom/dual-metal/composite structures to regulate the electronic structure and optimize the adsorption of intermediates	The selectivity of H_2_O_2_ is 93.1%, and the Faraday efficiency is 90.5%, the half-wave potential of the metal-free COF is 0.80 V	[43–46]
Constructing bifunctional COFs	Integrate ORR/OER active centers into the same framework, three-dimensional structure, composite materials, derivatives	Single catalyst achieves reversible oxygen catalysis	ΔE = 0.78 V. the half-wave potential of ORR is 0.85 V, and the overpotential of OER is 330 mV	[88–95]
Supramolecular engineering	*π*-*π* stacking, hydrogen bonds, van der Waals forces regulate the stacking pattern, pore environment, and interface behavior	Regulating the accumulation pattern and pore channel environment	The selectivity of H₂O₂ is 91%, and the mass activity is 12.2 A g⁻^1^, the electrochemical active area of 1D COF is 4.8 t that of 2D	[96–101]
Modulating edge sites	Modify the edge functional groups, create defects (such as dangling carbonyl bonds), control the spatial position of the active sites	High intrinsic activity, and capable of precisely controlling the spatial position	The selectivity of 2e^−^ ORR is greater than 99%, the yield of H_2_O_2_ is greater than 1200 mmol g^−1^ h^−1^	[102–106]
Building catalytic linkage	Optimize the connection bond types (such as C = C, oxazole, amide, pyrazine, etc.), introduce substituents or heteroatoms	Directly affects the electronic structure and stability	The TOF of the COF connected with oxazole is 1.9—7.4 t that of other connections, the selectivity of Br-COF ↑ by 112%, the half-wave potential is 0.933 V	[108–116]

**Table 5:** Quantitative performance enhancements of six structural design strategies

Strategy	Representative Material	Reaction	ORR *E*_1/2_(V vs. RHE)	OER η@10 mA cm^−2^ (mV)	Electrolyte	References
Metal anchoring	Fe-COF900 (ultra-close dual Fe sites)	ORR	0.82	–	0.1 M KOH	[71]
	CoO_x_@NC-800 (TRIPTA-Co derived)	ORR	0.89	–	0.1 M KOH	[68]
	Ir@COF-CaN	OER	–	280	1.0 M KOH	[77]
	iCOFs-Co (Co single atoms + Co NPs)	ORR	0.86	–	0.1 M KOH	[75]
	Thiophene-rich 3D COF (BUCT-COF-11)	ORR	0.72	–	0.1 M KOH	[46]
	Precise heteroatom COF	ORR	0.78	–	0.1 M KOH	[79]
Inducing polarization	Highly fluorinated COF	2e^−^ ORR	0.062	–	0.1 M KOH	[80]
	Piperazine-integrated COF (PD-COF-OH)	ORR	0.76	–	0.1 M KOH	[81]
	im-PY-BPY-COF (ionized imidazole)	ORR	0.80	–	0.1 M KOH	[85]
	CoTAPP-PATA-COF	ORR/OER	0.80	420	0.1 M KOH	[63]
	SUZ-101-Co (3D porphyrin)	ORR/OER	0.78	240	0.1 M KOH	[18]
Bifunctional COF	Fe, Co–N–C (derived)	ORR/OER	0.85	330	0.1 M KOH	[92]
	D-Tt-A COF + 3D graphene	ORR	0.88	–	0.1 M KOH	[88]
	Pyrrole-based COF (NUST-38)	OER	–	303	1.0 M KOH	[89]
Supramolecular engineering	DPPS-COF (side-chain engineered)	ORR	0.85	–	1.0 M KOH	[98]
	B18C6-PTTA-COF (pendant aldehyde)	2e^−^ ORR	(selectivity 91%)	–	0.1 M KOH	[97]
	JUC-660 (charged benzyl edge)	2e^−^ ORR	(H_2_O_2_ yield > 1200 mmol/g/h)	–	0.1 M KOH	[104]
Modulating edge sites	Edge-defect CON (suspended carbonyl)	2e^−^ ORR	(selectivity > 99%)	–	0.1 M KOH	[105]
	Oxazole-linked COF	ORR	0.75	–	1.0 M KOH	[108]
	FePc-BBL COF (ladder-type)	ORR	0.933	–	0.1 M KOH	[113]
	Br-substituted linker COF (Br-COF)	2e^−^ ORR	(selectivity 86.2%)	–	0.1 M KOH	[114]
Building catalytic linkage	Thienyl position isomerization (COF-B)	ORR	0.76	–	0.1 M KOH	[115]
	Nonpolar C = C linked COF (pyridine-centered)	ORR	0.74	–	0.1 M KOH	[111]
	COF-C_4_N with phenazine linkageCOF-C_4_N with phenazine linkage	OER	–	349	1.0 M KOH	[53]

### Functional Synthesis

This approach introduces new chemical components and functions to COFs through chemical modification or transformation. It can be further subdivided according to the modification timing into: pre-modification by designing functional monomers in advance before synthesis, in situ modification by achieving functionalization simultaneously during synthesis or pyrolysis, and post-modification by chemical processing on pre-synthesized COFs.

**Fig. 12:**
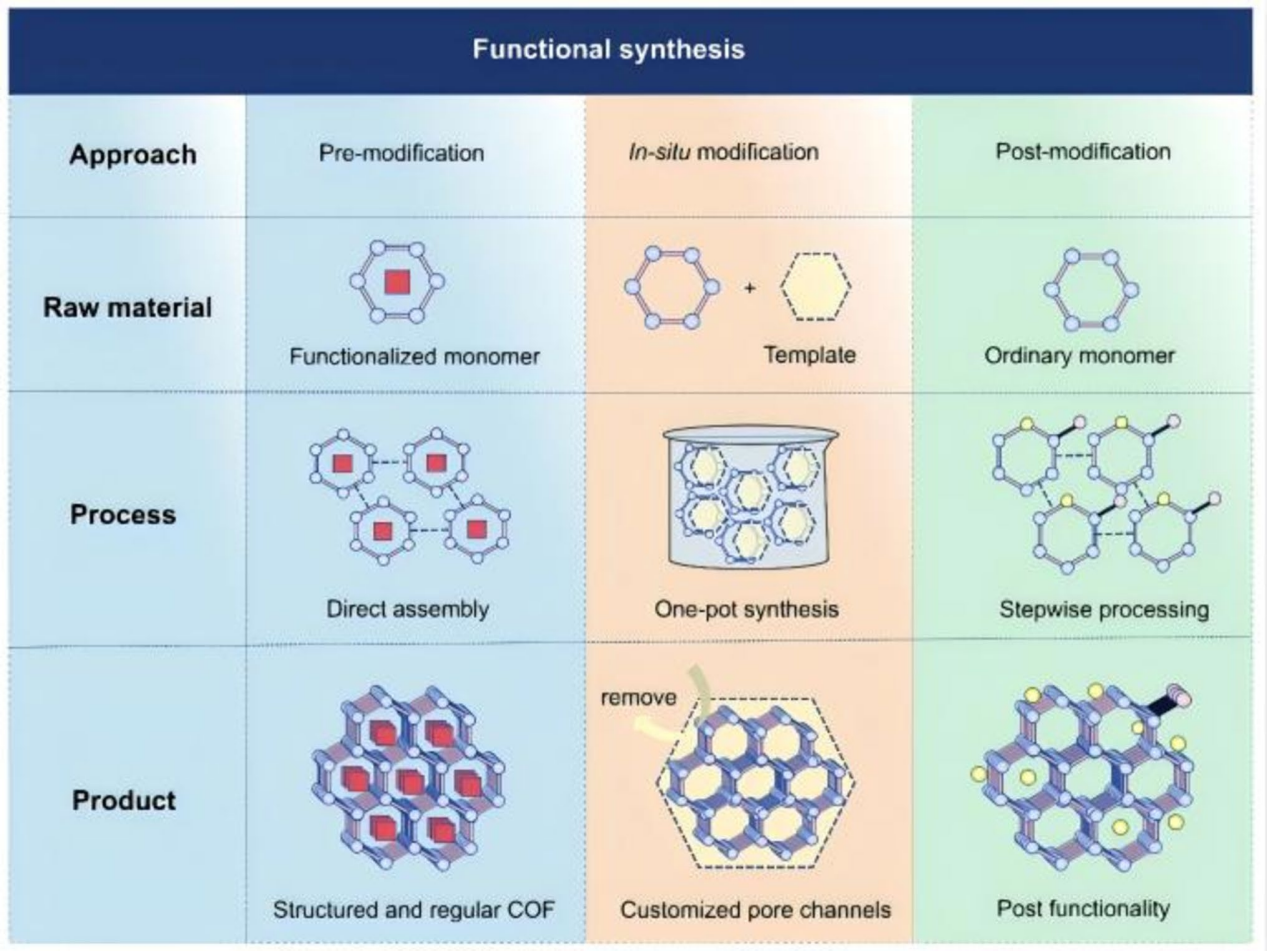
Brief illustration of functional synthesis approaches: pre-modification, in situ modification, and post-modification

In Sect. 3.1, we explored how to optimize the catalytic performance of COF materials through macroscopic structural design strategies such as metal anchoring, induced polarization, and supramolecular engineering. However, most of these strategies involve modifying and regulating the already formed framework. To achieve a breakthrough in performance, a more fundamental approach lies in precise design from the molecular source. This leads to the functionalization synthesis strategies discussed in this section, which involve introducing specific functional groups into the framework during or after the COF synthesis process to endow the material with pre-designed functions from the bottom-up. Although some concepts (such as metal anchoring and D-A effect) are presented in both chapters, their implementation methods are different. The structural design places more emphasis on internal integration, while this section focuses on external functionalization. This section will focus on three functionalization synthesis strategies: pre-modification, in situ modification, and post-modification. Pre-modification refers to the design and synthesis of monomers with target functional groups before COF synthesis, and then integrating the functional groups into the framework through polymerization, which has the advantage of precise control. In situ modification involves adding functional components to the reaction system during COF synthesis to form them simultaneously with the framework. Post-modification, on the other hand, introduces functional groups by secondary reactions using the pre-designed active sites on the COF framework after its synthesis (Fig. 12).

#### Pre-Modification

The pre-functionalization strategy refers to the design and synthesis of organic monomers with target functional groups before the synthesis of COFs, followed by the direct polymerization of these functionalized monomers to form COFs. Pre-modification can also be used to construct D-A structures, its electronic effect is similar to that discussed in Sect. 3.1.2. This design-first, assemble-later strategy allows researchers to precisely control the types of active sites, local electronic environments, and the overall topology of the framework at the molecular level. This strategy is one of the most effective methods for achieving high-performance and stable COF electrocatalysts. The following systematically elaborates on the diversity and powerful functions of the pre-functionalization strategy through several typical cases.

**Fig. 13:**
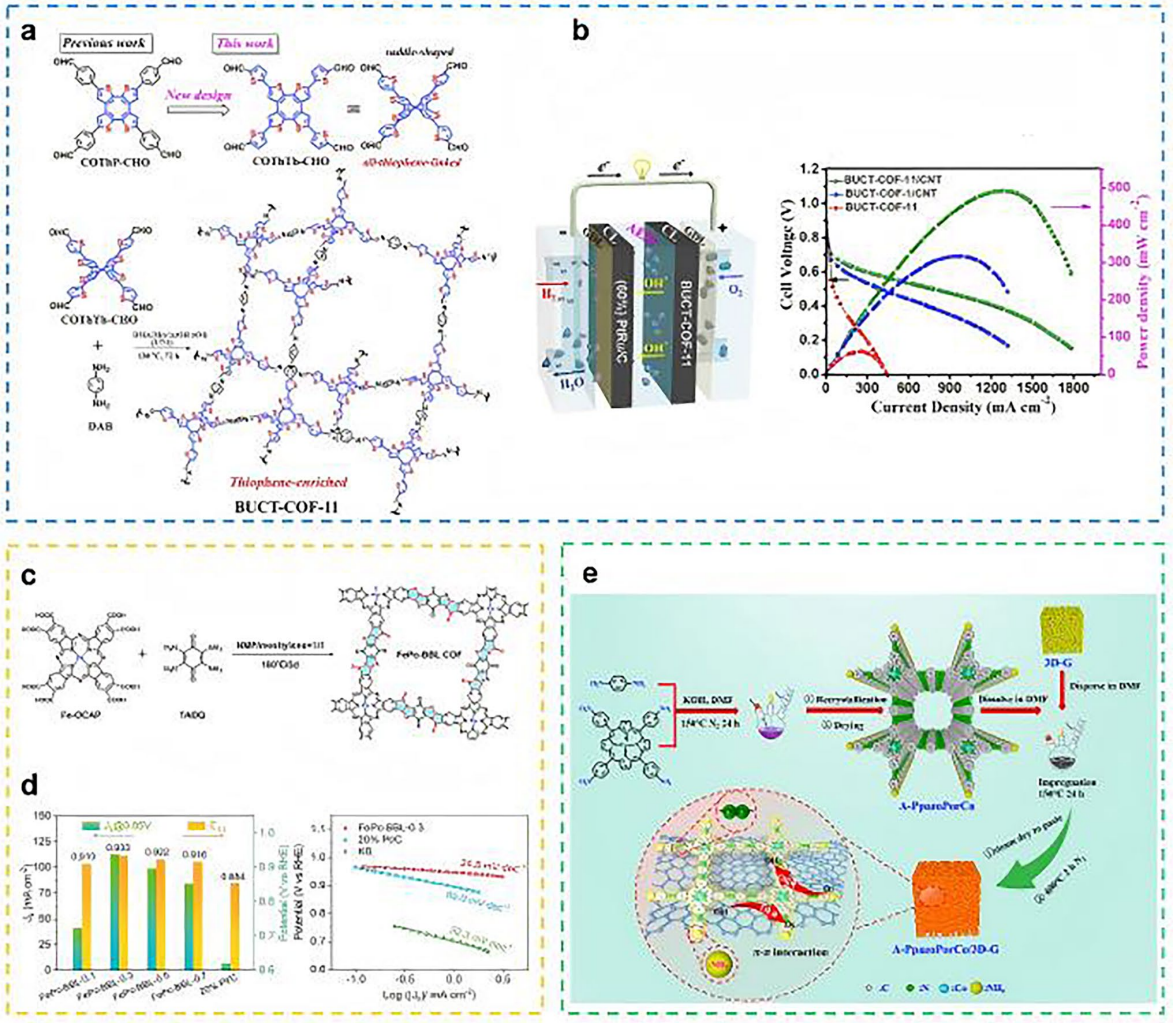
**a** Saddle-shaped structural unit used for constructing rich thiophene COFs, as well as the synthesis route of BUCT-COF-11 based on this unit. **b** Structural diagram of the AEMFC device is presented, and the polarization curves and power density curves of BUCT-COF-11/CNT (green), BUCT-COF-1/CNT (blue), and the unmodified BUCT-COF-11 (red) are compared in the H_2_/O_2_ type AEMFC (80 °C, 100% relative humidity) [46]. Copyright 2022, Wiley. **c** Schematic of the synthesis and structure of FePc-BBL COF through the condensation of Fe-OCAP and TABQ. **d** Dynamic current density and half-wave potential of the FePc-BBL-X series materials and the 20% Pt/C catalyst at 0.85 V, as well as the Tafel curves of FePc-BBL-0.3, 20% Pt/C and KB Tafel curves of FePc-BBL-0.3, 20% Pt/C, and KB. Tafel slope measured in O_2_-saturated 0.1 M KOH, scan rate 5 mV s^−1^ [113]. Copyright 2022, Elsevier. **e** Preparation of A-PpazoPorCo and A-PpazoPorCo/3D-G [88]. Copyright 2025, Elsevier

The pre-functionalization can precisely control the structure, which is an effective way to construct high-stability and high-conductivity catalytic frameworks. This strategy is mainly reflected in the construction of three-dimensional networks and ladder-type structures. Researchers designed a fully thiophene-linked saddle-shaped building unit and successfully synthesized a fully conjugated three-dimensional COF (Fig. 13a). This ingenious pre-functionalization design simultaneously solved the two major problems of poor conductivity and lack of efficient non-metallic active sites in 3D COFs. The electron-rich thiophene units not only formed the conjugated framework of the entire structure, greatly enhancing the intrinsic electrical conductivity of the material, but also served as optimized ORR active centers. Theoretical calculations confirmed that the introduction of thiophene optimized the electronic structure of the material, thereby enhancing the ORR performance. When this COF was used as a cathode catalyst to assemble an anion exchange membrane fuel cell, a peak power density of up to 493 mW cm^−2^ was achieved, which strongly demonstrated the practical potential of COF materials as metal-free catalysts in fuel cells and highlighted the great prospects of fully conjugated 3D COFs in the energy field (Fig. 13b) [46].

In line with this, in another study, researchers carefully designed two monomers, octacarboxy iron phthalocyanine and tetrabenzoquinone, to form a ladder-type two-dimensional COF with benzimidazole–benzophenanthroline linkages during polymerization (Fig. 13c). This key pre-functionalization design resulted in a rigid and stable aromatic framework in the final structure. This structure not only ensured extremely high chemical stability and excellent charge transport capability but also perfectly exposed atomically dispersed Fe–N_4_ active centers in open channels. The resulting COF exhibited extraordinary ORR performance in alkaline media, with a half-wave potential as high as 0.933 V and a Tafel slope as low as 24.8 mV dec^−1^ (Fig. 13d). The turnover frequency and mass activity reached 16.4 and 57.2 t that of Pt/C, respectively. When applied to zinc–air batteries, it also demonstrated superior power density and cycling stability compared to Pt/C, providing a new approach for designing efficient and stable electrocatalysts without the need for pyrolysis [113].

Based on the construction of an ideal framework, the precise electronic structure regulation of active centers through pre-functionalization is the key to enhancing their intrinsic catalytic activity. As discussed in Sect. 3.1.2, the induced polarization strategy can be implemented through donor–acceptor (D-A) motifs. The pre-modification approach offers a more precise way to integrate such motifs directly into the framework during synthesis. For example, researchers pre-designed cobalt porphyrin (donor) and benzothiadiazole (acceptor) as monomers, assembling COFs with rhm and sql topologies [82]. The strongly electron-withdrawing BTD units modulate the electron density of the Co–N_4_ sites via conjugation—a mechanism detailed in Sect. 3.1.2. The key advantage here is that pre-modification avoids post-synthetic inhomogeneity and yields a well-defined, crystalline framework with atomically dispersed active sites. The resulting Co@rhm-PorBTD achieved a mass activity 5.8 t that of Pt/C. Meanwhile, a more advanced study successfully synthesized Fe_2_COF by designing building units with pre-integrated bidentate iron chelating sites. Subsequently, through a one-step carbonization process, this COF precursor was directly transformed into a bonded bimetallic catalyst with a well-defined Fe_2_N_6_C_8_O_2_ configuration [117]. The core of this strategy lies in the integration of the bimetallic active centers into the COF structure through the design of functional monomers before synthesis, with the carbonization process merely converting it into its final active form. Thanks to this precise pre-modification design, the resulting catalyst has an optimized d-band center and enhanced adsorption of the OOH* intermediate, thereby demonstrating outstanding ORR activity. In contrast to the strategy of introducing strong electron-withdrawing units, another approach is to introduce electron-donating units. Researchers successfully synthesized a cobalt porphyrin-based COF with strong electron-donating amino-ph-NH_2_ groups by adjusting the ratio of reaction monomers (Fig. 13e). In this design, the strongly electron-donating amino groups, along with the electron-donating azo bonds and the electron-accepting cobalt porphyrin center, form an efficient donor-*π*-bridge-acceptor structure. This significantly increases the electron cloud density of the cobalt porphyrin center, thereby optimizing its adsorption behavior toward reaction intermediates and ultimately achieving excellent bifunctional oxygen catalytic activity [88].

Furthermore, the deep integration of pre-modification strategies with computational science is driving this field from empirical exploration to rational design. A pioneering study combined density functional theory calculation with machine learning to conduct a systematic study of a series of transition metal single-atom catalysts anchored on dioxin-linked phthalocyanine COFs. This work not only predicted that materials such as CoPc-TFPN are excellent hydrogen evolution and bifunctional oxygen catalysts, with CoPc-TFPN showing OER/ORR overpotentials as low as 0.18/0.36 V [118], but also revealed through machine learning the key indicators determining catalytic activity, namely in addition to the classic d-band center, the number of d-orbital electrons also plays a crucial role.

#### In Situ Modification

Although pre-modification operations have high accuracy, they still require the design of functional monomers, and these monomers are often difficult to synthesize. In situ modification provides an alternative solution, which is to introduce functional components during the growth of COFs. This usually simplifies the process and achieves a tighter interface.

**Fig. 14:**
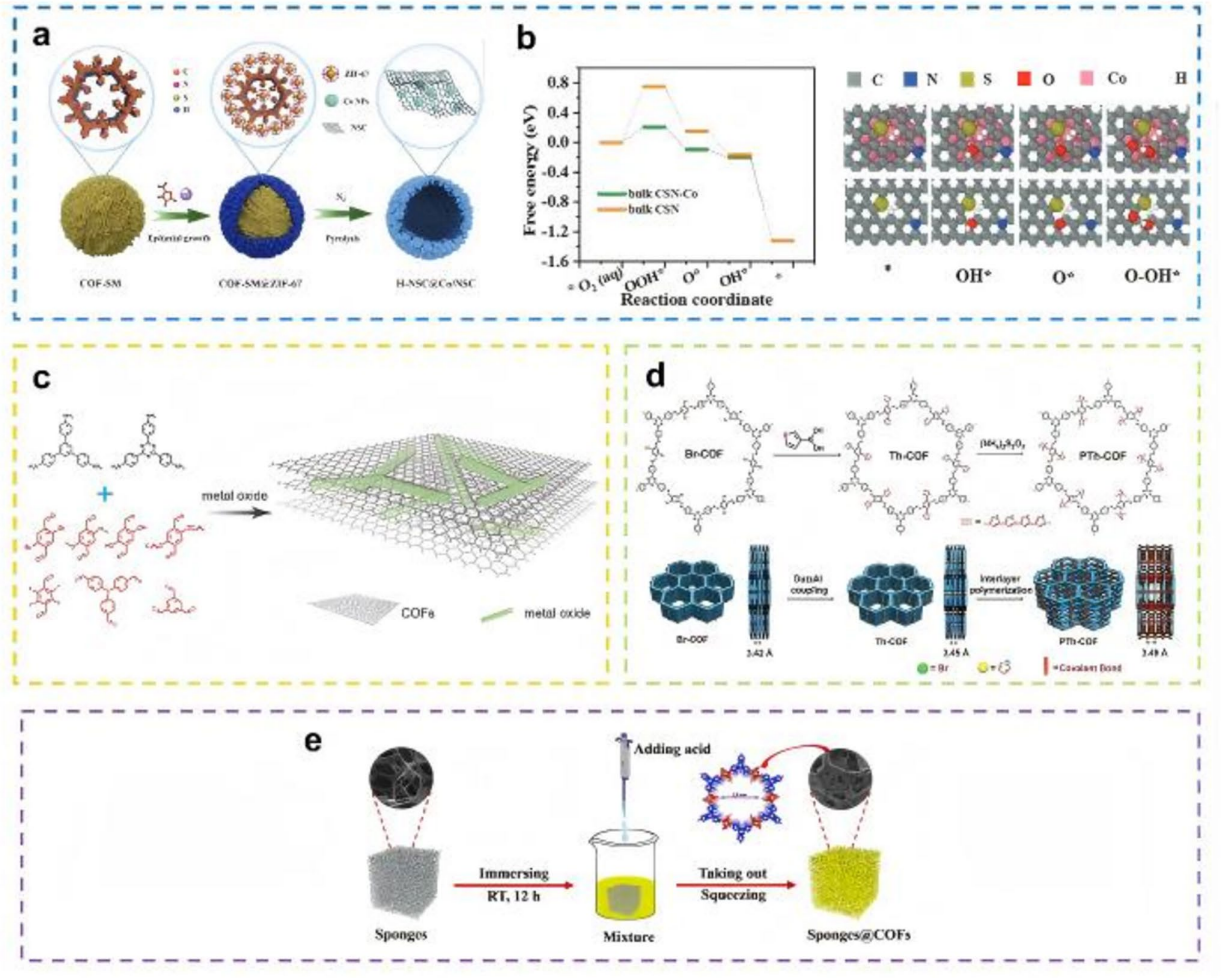
**a** Schematic illustration of the synthesis process of core–shell H-NSC@Co/NSC hybrid. **b** Graph showing the change in free energy of the redox reaction between the bulk CSN-Co and the CSN system at an electrode potential of 0.9 V (relative to the standard hydrogen electrode), as well as the structural model of the bulk CSN-Co Free energy calculations performed at 0.9 V vs. RHE, pH = 14 [119]. Copyright 2022, Wiley. **c** Schematic showing the synthesis of COFs using metal oxides [120]. Copyright 2021, American Chemical Society. **d** Synthetic route and structure of fully conjugated PTh-COF (with the success of interlayer polymerization, the layer spacing becomes larger) [47]. Copyright 2024, Wiley. **e** Synthetic scheme of sponges@COFs [121]. Copyright 2021, Elsevier

In situ modification can realize the in situ composite of COF with other functional materials, thereby constructing hybrid materials with synergistic effects. For example, by using in situ modification to composite COF with metal–organic frameworks, an ideal core–shell precursor can be constructed. Researchers have used a one-pot method to connect COF with ZIF-67 MOF through covalent bonds, forming a clearly structured COF@ZIF-67 core–shell hybrid material (Fig. 14a) [119]. The key of this in situ hybridization strategy lies in its covalent interface that enables the subsequent thermal decomposition process to maintain the highly dispersed active components. Therefore, after the material undergoes thermal decomposition, this precursor gives rise to a carbon-based electrocatalyst with a core–shell structure, and the MOF-derived shell is rich in cobalt nanoparticles, while the COF-derived core is nitrogen–sulfur-doped carbon. Theoretical calculations confirm that the cobalt nanoparticles embedded in the material can effectively regulate the electronic structure of the carbon backbone and optimize the adsorption of oxygen intermediates, thereby endowing the material with excellent OER/ORR bifunctional activity (Fig. 14b). Similarly, metal oxides can also participate in the synthesis of COF as catalysts and functional components (Fig. 14c) [120]. In this strategy, metal oxide nanoparticles not only catalyze the formation of COF but are also encapsulated in the final framework, thus achieving the unification of synthesis and functionalization.

In addition to the aforementioned composite of COF with other functional materials, in situ modification can fundamentally solve the bottleneck of its intrinsic performance by triggering the structural transformation of COF itself. Recent breakthroughs have come from the interlayer polymerization strategy. Researchers have synthesized a full-conjugated material named PTh-COF by in situ polymerization of thiophene in the nanochannels of two-dimensional COF (Fig. 14d) [47], creating a linear polythiophene chain as a conjugated bridge, which creates an efficient cross-layer charge transport channel. Theoretical calculations show that the interlayer conjugated polythiophene optimizes the electron cloud distribution and narrows the band gap, thereby significantly enhancing the ORR activity. This strategy of triggering the structural transformation of COF provides an innovative solution to overcome the problem of poor interlayer conductivity of two-dimensional COF. Moreover, the flexibility of this strategy is also reflected in its ability to construct an ideal macroscopic morphology for catalysts. Through one-pot room-temperature synthesis, COF coatings can be in situ grown on the surface of a three-dimensional sponge framework (Fig. 14e) [121]. Although this study focuses on oil–water separation, its mild and efficient in situ composite strategy has important application value. It demonstrates the feasibility of uniformly loading electrocatalytic COF onto a three-dimensional conductive substrate, which is crucial for constructing self-supported electrodes with high activity site density, rapid mass transfer ability, and stable contact.

Furthermore, particularly notable is that the use of emerging physical fields such as radiation can drive in situ modification, opening up new ways to create special active centers under mild conditions. Gamma-ray radiation-induced one-pot synthesis can directly synthesize mixed valence state copper (Cu(I)/Cu(II)) coordinated COF [122]. Radiation induces the in situ reduction of Cu^2+^ to Cu^+^, precisely constructing unique mixed valence state active sites. This method, unlike the post-synthesis method, which leads to structural degradation, instead improves the crystallinity and porosity of the material. Furthermore, this radiation-induced platform also supports synchronous synthesis and graft polymerization, where functional molecular chains are in situ grafted onto the COF framework at the early stage of its formation [123]. This enables the complete synthesis and functionalization to be accomplished in one step, providing a powerful tool for precisely introducing catalytic active functional groups.

In summary, in situ modification has achieved precise integration of functions and deep optimization of structure during the formation of COFs through various means such as one-pot composite, internal structure engineering, and physical field-driven methods. It is worth noting that, as pointed out in the systematic review on COF/graphene hybrid materials [124], one-pot methods and in situ growth strategies have an irreplaceable advantage in constructing tight interfaces.

#### Post-Modification

Compared with the in situ modification strategy that realizes functionalization simultaneously during the synthesis process, the post-synthetic modification (PSM) strategy embodies a modular design concept of first constructing the framework and then performing the modification. This strategy aims to introduce target functional groups or active centers on the pre-synthesized and structurally stable parent COF framework through further chemical reactions [15]. The core advantage of this method lies in its high flexibility and wide applicability, allowing researchers to independently optimize the stability and pore structure of the base COF matrix and the catalytic function of the introduced groups, thereby avoiding the synthesis difficulties or framework instability problems that may arise from functionalized monomers.

It is particularly important to note in this section that post-synthetic modification is a key means for implementing many structural design strategies described in Sect. 3.1. For example, the metal anchoring in Sect. 3.1.1, the most common and precise implementation method is through coordination interactions in post-synthetic modification. The induced polarization effect in Sect. 3.1.2 is often achieved by introducing groups with strong electron-withdrawing or electron-donating capabilities. For the dual-function construction in Sect. 3.1.3, post-synthetic modification provides the possibility of integrating different active sites step by step on a single framework. Therefore, the content of this section is complementary to the previous structural design concepts, jointly forming a complete means for the functionalization of COF materials.

A review article in 2019 summarized the main methods for post-synthetic modification of COFs [125]. This paper first proposed five main pathways for post-synthetic modification of COF, including the introduction of metal species through coordination chemistry, functionalization modification through covalent bonds, chemical transformation of connecting units, internal functionalization by truncating monomers, and block exchange strategies. This complete classification system provides a solid theoretical basis and methodological guidance for subsequent studies on various post-synthetic modifications.

**Fig. 15:**
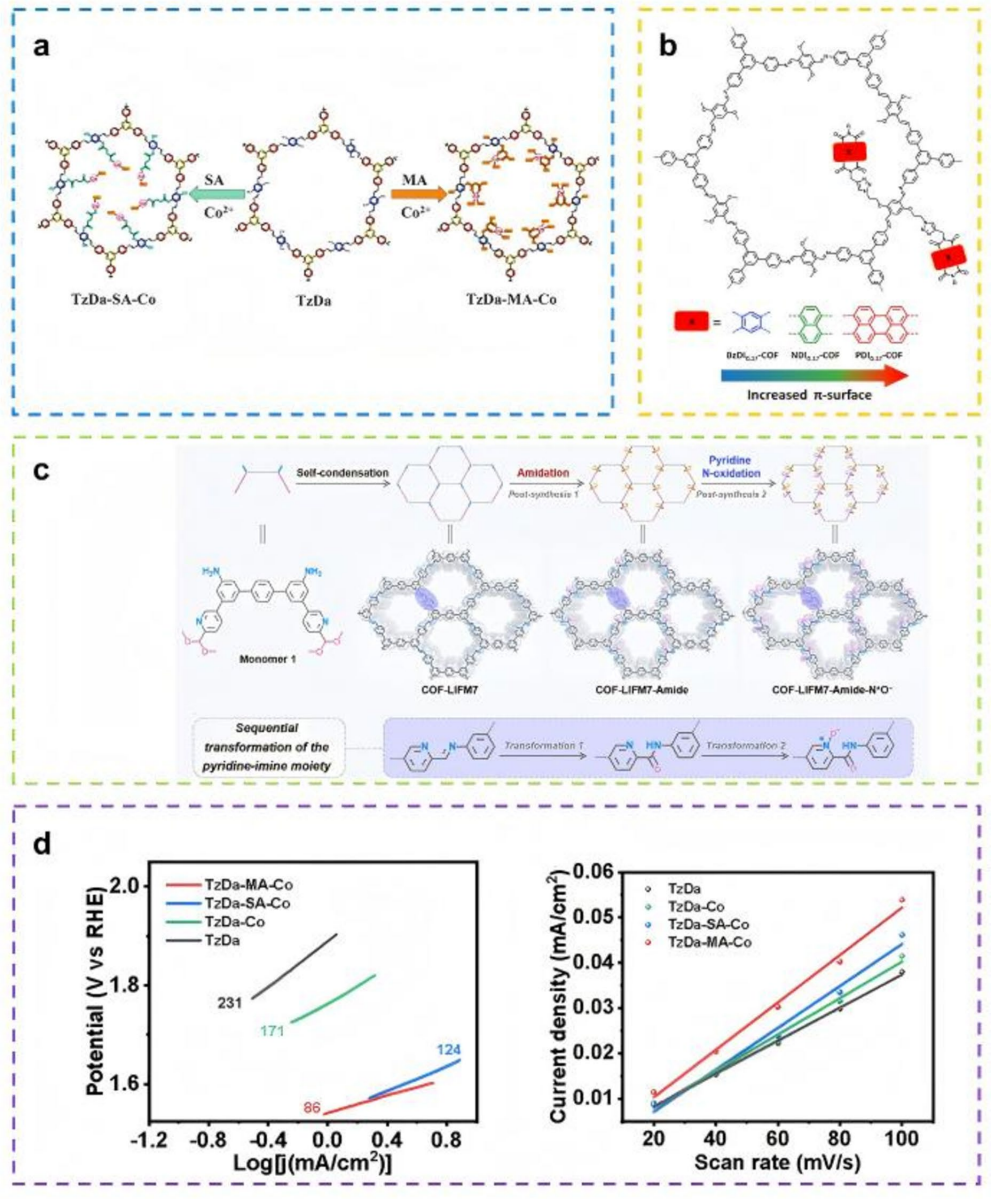
**a** Schematic illustration of the designed strategy for Co^2+^ anchoring in COFs with a simplified repeat unit. Reproduced with permission [96]. Copyright 2025, World Scientific Pub. **b** Schematic representation of XDI_0.17_-COFs where X represents different *π*-surfaces from left to right: benzene, naphthalene, and perylene [127]. Copyright 2023, American Chemical Society. **c** COF-LIFM7 adopts a design strategy of sequentially modifying the imine structure into amide groups and pyridine N-oxide groups to enhance polarity. The generation process of its derivatives, COF-LIFM7-Amide and COF-LIFM7-Amide-N^+^O^−^, is presented, along with the gradual transformation process of the pyridine–imine structural units [128]. Copyright 2025 Wiley. **d** Based on the OER polarization curves, the Tafel diagrams of four COFs were obtained, and the relationship between the capacitance current and the scanning rate was analyzed by the CV curves under different scanning rates, Electrochemical impedance spectroscopy (EIS) performed at overpotential of 300 mV, frequency range 0.1 Hz-100 kHz, amplitude 5 mV [126]. Copyright 2025, World Scientific Pub

In the field of metal post-synthetic modification, the coordination chemistry-based metal anchoring strategy is one of the most direct and effective post-synthetic modification methods. On this basis, a study has developed a more refined covalent anchoring strategy, developing a specific covalent anchoring method based on hydroxyl sites. This method employs Meldrum’s acid bridge ligands to achieve strong chelation of cobalt ions on the walls of TzDa carbon nanotubes (Fig. 15a) [126]. The obtained TzDa-MA-Co material exhibits excellent OER performance at 10 mA cm^−2^ (Fig. 15d), with a Tafel slope as low as 86 mV dec^−1^, and remains stable during a 40-h continuous test.

Dual-metal synergistic modification represents an advanced form of post-synthetic modification, further expanding the application boundaries of post-synthetic modification. In recent years, research has made significant breakthroughs in using the post-synthetic modification strategy to construct dual-metal synergistic sites. Researchers have established a complete dual-metal catalyst screening platform through systematic studies of the coordination behavior of pyridine-based COF with various transition metals. The research found that the COF-Bpy@FeNi combination exhibited the best OER performance, requiring only 399 mV overpotential to achieve a current density of 10 mA cm^−2^ under alkaline conditions [24]. In-depth density functional theory (DFT) calculations revealed the synergistic mechanism of the Fe–Ni bimetallic system, with the Ni sites serving as the main catalytic center, while the Fe sites indirectly optimized the catalytic performance by regulating the electronic structure of the Ni ions. Based on this, another study utilized a post-synthesis strategy in a more complex multi-component environment to construct bimetallic sites and achieved an efficient bifunctional catalyst. The researchers constructed an efficient bifunctional oxygen electrocatalyst through indium doping of iron coordination and studied its mechanism. Additionally, this material demonstrated excellent comprehensive performance in zinc–air battery tests: the half-wave potential and starting potential reached 0.86 and 1.09 V, respectively, and there was almost no degradation after 20,000 cycles. This outstanding stability stems from the synergistic protection effect of doping-induced resistance to leaching and the porous structure.

Beyond the traditional metal coordination strategy, subsequent research demonstrated the diversity and precision of post-synthesis strategies. Researchers precisely introduced various imide-type active centers in the COF framework through click chemistry (Fig. 15b) [127], achieving precise site control of 7.65 × 10^−4^ mol mg^−1^. This highly controllable modification method established an ideal model system for studying the ORR performance of metal-free and non-decomposition materials. In alkaline medium, NDI_0.17_-COF exhibited the best ORR activity, with an initial potential of 0.77 V vs RHE, and the perfect agreement between theoretical calculations and experimental results confirmed the value of this strategy in establishing accurate structure–activity relationships. Another study addressed the instability of imide bond COF in water, and researchers developed an innovative stepwise post-synthesis strategy. Through consecutive amidation reactions and pyridine N-oxidation modification (Fig. 15c) [98], the stability and hydrophilicity of the material were significantly enhanced while maintaining crystallinity and porosity. The final obtained material achieved a proton conductivity of 1.9 × 10^*−*3^ S cm^−1^ at 95% relative humidity and 70 °C, which was three orders of magnitude higher than the original material.

Finally, the heterostructure construction expanded the application scope of post-synthesis. One study combined fluorinated porphyrin COF with graphene oxide through covalent anchoring, ingeniously combining the high catalytic activity of COF and the excellent conductivity of graphene [129]. This composite strategy not only significantly improved the charge transfer efficiency but also provided a new idea for solving the inherent conductivity deficiency of COF-based electrocatalyst, demonstrating the powerful integration ability of post-synthesis in constructing complex functional materials. The development history of post-synthesis strategies clearly shows the evolution path from simple to complex, from single to multiple, from precise click chemistry modification to ingenious stepwise post-synthesis, and to cross-dimensional hetero-composition. Post-synthesis technology continuously breaks through the boundaries of existing technologies. These advancements not only expanded the functional diversity of COF-based electrocatalyst but also laid a solid foundation for their practical applications in energy conversion.

Table 6 provides a concise comparison of these three functional synthesis methods (pre-modification, in situ modification, and post-modification), listing their definitions, advantages, and limitations.

**Table 6:** Comparison of functional synthesis strategies for COF-based electrocatalysts (advantages and limitations)

Functional synthesis	Definition	Advantage	Limitation	Representative performance	References
Pre-modification (3.2.1)	Before synthesis, design monomers containing the target functional groups. After polymerization, the functionalized framework is directly formed	Precisely control the type of active sites, the electronic environment, and the topological structure, this can avoid the unevenness of post-modification	The synthesis of functionalized monomers is difficult, it may interfere with the polymerization reaction or reduce the crystallinity	ORR half-wave potential: 0.933 V, mass activity: 57 t that of Pt/C, AEMFC peak power density: 493 mW cm^−2^	[46, 82, 88, 113, 117, 118]
In situ modification (3.2.2)	During the synthesis process, the second component (MOF, metal oxide, polymer chain) is simultaneously introduced or the self-structure of COF is induced to undergo transformation	One-step process simplification, interface is tight (such as covalent connection), unique structures can be constructed (such as core–shell, interlayer polymerization)	The reaction conditions need to be compatible with all components, it might be difficult to optimize each step independently	The COF@ZIF-67 derived carbon catalyst achieves dual-functionality for ORR/OER, the interlayer conductivity of PTh-COF is significantly enhanced	[47, 119–123]
Post-modification (3.2.3)	After the COF synthesis, functions are introduced through coordination, covalent bonds, click chemistry, etc	High flexibility, wide applicability, does not damage the crystallinity and pore structure of the parent material, can gradually introduce multiple functions	The loading amount of functional groups may be uneven, it may clog the pores, it is necessary to ensure that the reaction conditions do not damage the framework	The synergistic OER overpotential of bimetallic system is 399 mV, the density of the click chemistry introduced imide site is 7.65 × 10^*−*4^ mol/mg	[13, 30, 126–129]

## Mechanism Discussion for Performance Improvement

### Discussion on the Mechanism

After introducing the strategies for obtaining high-performance COF-based electrocatalyst through structural design and functional synthesis, the underlying mechanisms of the performance improvement should be discussed [130, 131]. We will explore the synergy mechanism of active site design, the regulation of electronic structure, mainly focusing on the disruption of symmetry. Finally, we will discuss the control mechanisms of reaction pathways through supramolecular engineering, modulation of edge sites, and the construction of catalytic linkages.

#### Synergistic Effect

**Fig. 16:**
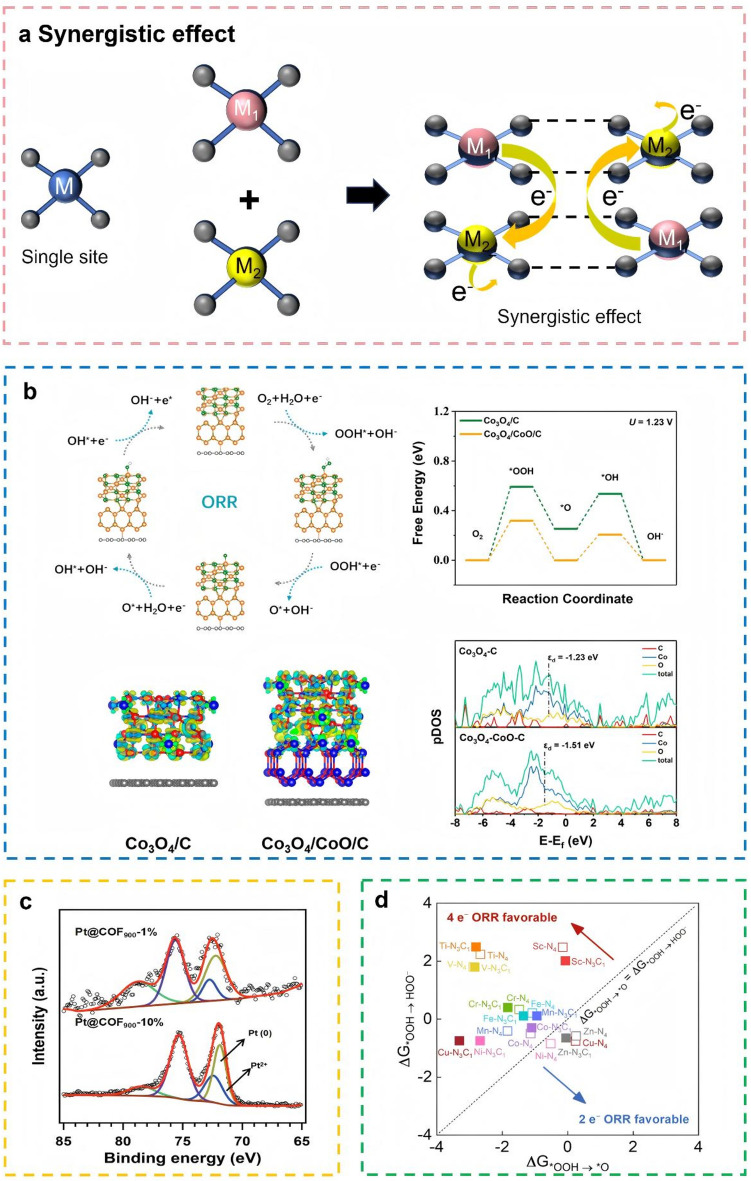
**a** Typically synergistic effects in oxygen catalysis. **b** DFT calculation results, charge density distribution, and state density diagrams of the Co_3_O_4_/CoO/C model [68]. Copyright 2023, American Chemical Society. **c** High-resolution XPS of Pt for 1%-Pt-COF900 (the upper) and 10%-Pt-COF900 (the lower) [76]. Copyright 2022, Wiley. **d** Gibbs free energy changes for OOH → O vs. *OOH → HOO^−^ across different active sites [17]. Copyright 2025, American Chemical Society

The oxygen catalytic action of COF-based electrocatalyst originates from the active sites. Previously, most studies mainly focused on single-metal sites. In recent years, research has shifted from traditional single-metal sites to dual metals or multi-metals, as well as multi-component systems such as Co_3_O_4_-CoO nanoparticle systems or the interaction of different forms of the same substance such as platinum monomers and platinum nanoparticles. It can be summarized that the multi-component in the material does not simply add up the performance but rather exhibits a complex synergy mechanism. This synergy mechanism not only enhances the activity of the central site but also significantly improves the stability of the catalyst (Fig. 16a). From the electronic level, the heteronuclear dual metals undergo charge transfer due to the difference in electronegativity, which effectively regulates the d-band positions of each metal center. From the geometric level, the specific spatial configuration of the dual-metal site may provide the most suitable adsorption configuration for different reaction intermediates. More importantly, the stable interaction between dual-metal sites can solve the problems of metal overflow and agglomeration commonly found in single-atom catalysts. From the component level, the CoOx@NC-800 system demonstrates the synergistic mechanism between metal oxides and carbon matrix. DFT calculations show that this synergy not only optimizes the adsorption energy of the OOH* intermediate, but more importantly, through the redistribution of interface charges, it changes the energy barrier of the reaction path (Fig. 16b) [68]. It is particularly noteworthy that the unique structure of hollow CoOx nanoparticles and layered carbon nanofibers creates abundant interface regions, which may play a dominant role in different reaction stages, thus achieving true bifunctional catalysis. In terms of scale dimensions, the cooperative system of Pt monomers and nanoparticles reveals the complementary effect between different forms of the same metal element [76]. The possible mechanism lies in: the single-atom site optimizes the adsorption of specific intermediates by its unsaturated coordination environment, while the nanoparticles provide efficient electron conduction pathways and additional active sites (Fig. 16c). The advantage of this multi-scale synergy lies in maintaining the high atomic utilization of single-atom catalysts while leveraging the excellent conductivity of nanoparticles, achieving an ideal balance between activity and stability (Fig. 16d) [17, 132].

#### Regulating Electronic Structure

**Fig. 17:**
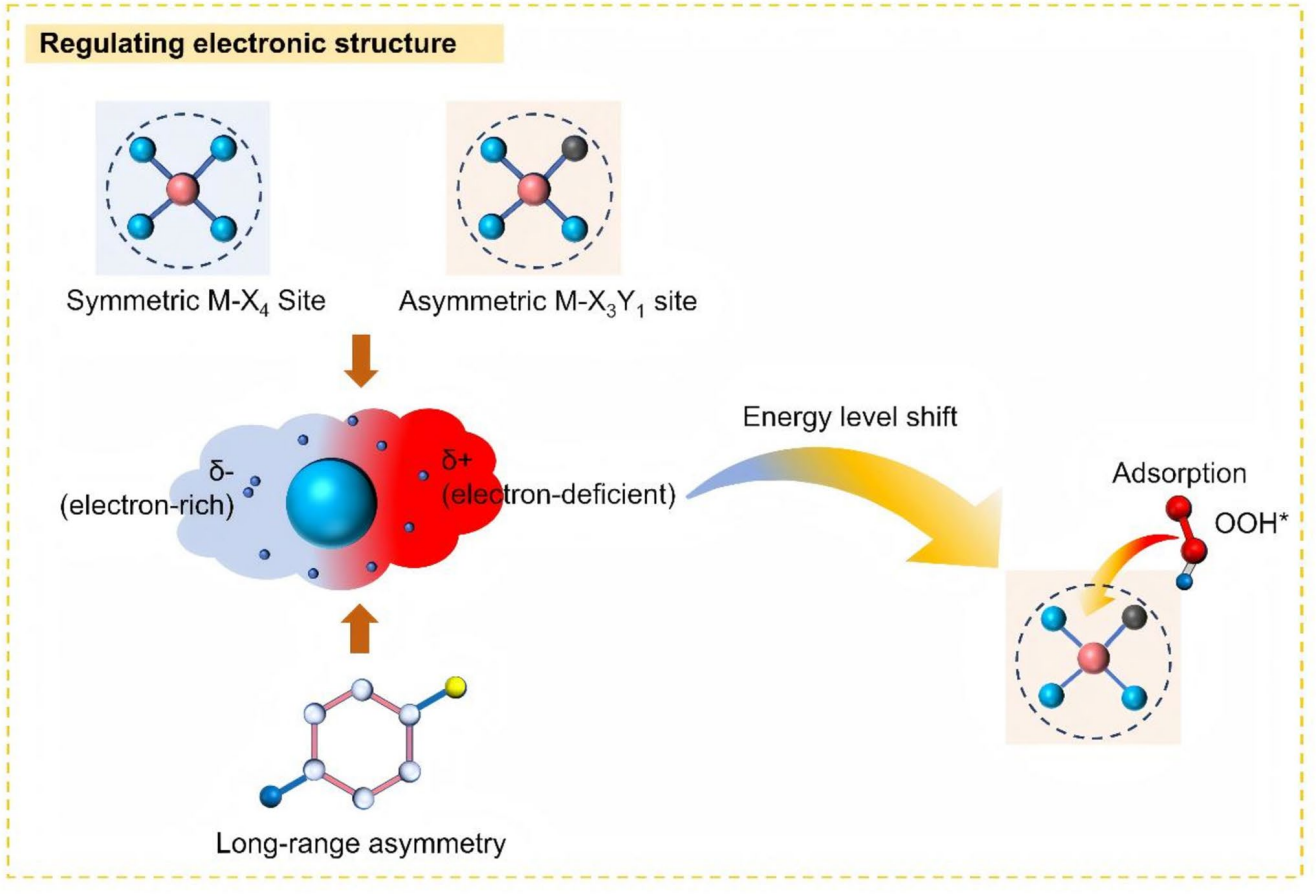
Illustrates the schematic diagram of electronic structure regulation in electrocatalysts based on COF

As the core mechanism for improving catalytic performance, it has been proved that any effective performance improvement strategy must ultimately be achieved by changing the electronic state density, energy level distribution, or charge layout of the active sites. This electronic-level regulation directly determines the adsorption strength of reaction intermediates at the active sites, thereby affecting the activation energy barrier and the selectivity of the reaction pathway. The key lies in breaking symmetry [133]. In local coordination, as demonstrated in the study of N-ambiguous porphyrin COF [17], researchers transformed the Co–N_4_ site into the Co–N_3_C_1_ site, thus breaking the symmetry of the coordination field, and further exploring its mechanism. Essentially, researchers utilized the differences in electronegativity and coordination ability between carbon and nitrogen atoms to achieve fine regulation of the electronic distribution of the cobalt center, this minor structural change significantly increased the electron density of the metal center and also changed the steps determining the reaction potential energy, thereby optimizing the thermodynamic reaction barrier of the entire reaction pathway. Additionally, on a long-term scale, the strategy described in the previous section, namely the induced polarization in Sect. 3.1.2, artificially creates an asymmetry in the electron distribution over a larger spatial range, which can be regarded as breaking the symmetry from a distant perspective. Among them, the doping of heteroatoms utilizes the inherent electronegativity differences of atoms to generate permanent and localized dipole moments on C–X (*X* = N, O, S, etc.) bonds [79]. Moreover, by introducing strong electron-withdrawing groups such as fluorine atoms or main group metal dopants like indium and antimony, an intrinsic electric field from donor to acceptor can be created on the conjugated framework of COF. This built-in electric field acts as a powerful driving force, forcing electrons to undergo directional migration within the *π* conjugated system, thereby inducing local charge enrichment or depletion at the active centers far from the doping sites. The study on the modulation of pyridine nitrogen charge has confirmed that giving it more negative charges makes it a more efficient electron donor [85]. The high-throughput calculation screening of main group metals further reveals that although they do not directly participate in catalysis, they have an electron-regulating effect, and the strong polarization effect introduced by them can significantly regulate the p-band center of adjacent active sites. Whether it is local coordination or long-range induced polarization, the ultimate goal is to break the electronic symmetry of the system, creating active sites with the optimal adsorption strength for specific reaction intermediates, thereby laying the foundation for a high catalytic activity electronic structure [134] (Fig. 17).

#### Controlling Reaction Pathway

**Fig. 18:**
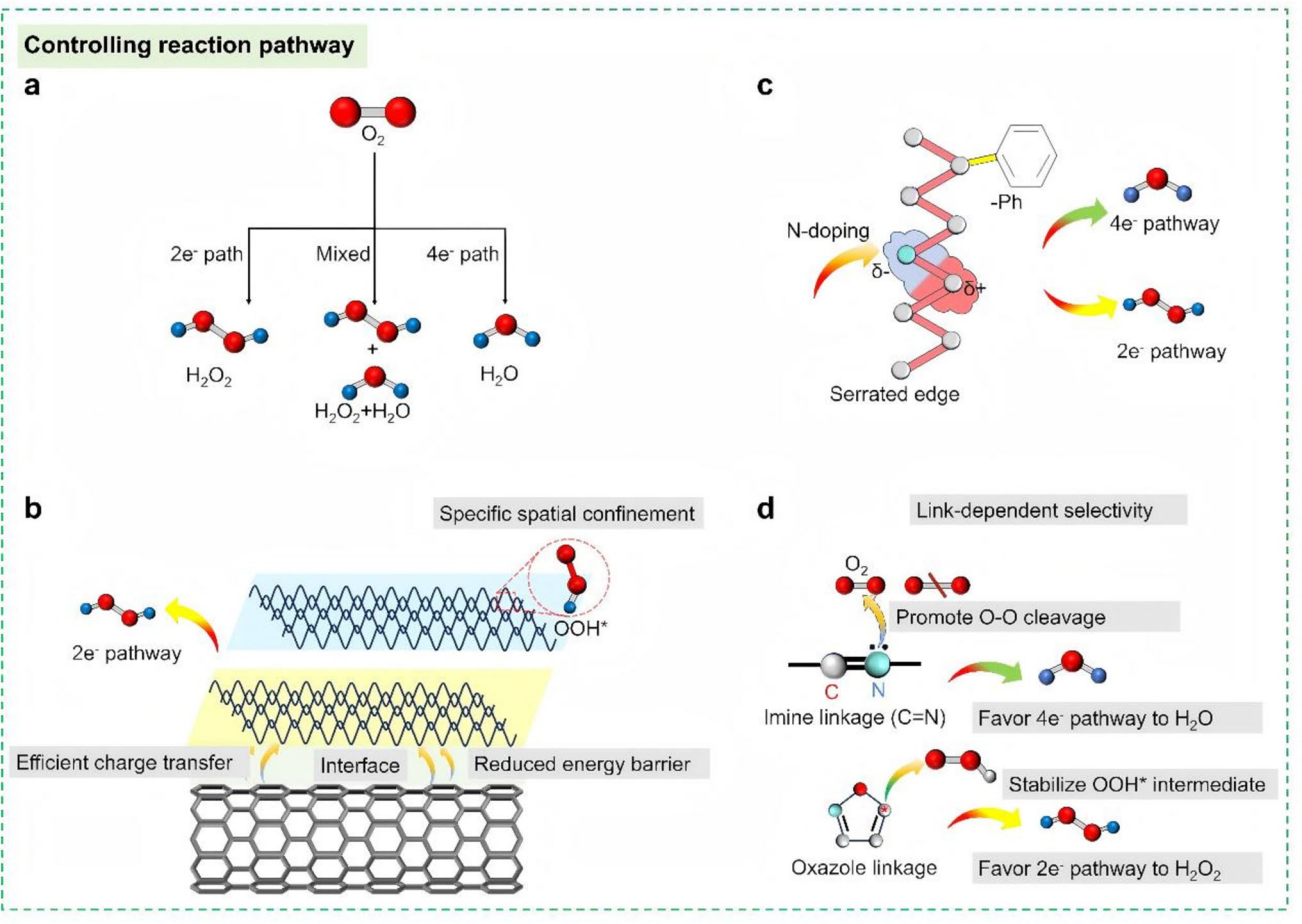
Schematic diagram of reaction path control for ORR/OER. **a** General ORR pathway selection. **b** supramolecular engineering for pathway control. **c** Modulating edge sites for pathway control. **d** Building catalytic linkages for pathway control

In the field of electrocatalysis, the selectivity of reaction pathways directly determines the efficiency and product distribution of the reaction. For example, in the ORR, the competition between the 2e^−^ path for generating hydrogen peroxide and the 4e^−^ path for generating water (Fig. 18a). COF provide an ideal platform for achieving precise control of reaction pathways due to their customizable molecular structure. Research on supramolecular engineering, modulating edge sites, and building catalytic linkage has elevated catalyst design from activity optimization to pathway control. We will discuss the underlying mechanisms of these three strategies next.

Supramolecular engineering, as a means of regulation at the macroscopic level, precisely manipulates non-covalent interactions between molecules to construct an ideal physical framework and interface environment for optimizing reaction pathways. For instance, by designing pendant aldehyde groups and inducing them to form an interlaced stacking pattern, not only is the mass transfer space significantly increased, making the active sites more accessible, but more importantly, this stacking pattern provides a specific spatial orientation for the reaction intermediate OOH*, thereby favoring the 2e^−^ reduction path. Further, expanding the material dimension from two-dimensional to one-dimensional fundamentally solves the bottleneck of poor accessibility of basal active sites, significantly improving site utilization and reaction kinetics. When the supramolecular assembly strategy extends to heterointerfaces, such as constructing a van der Waals heterojunction between COF and carbon nanotubes, the efficient charge transfer at the interface can significantly reduce the reaction energy barrier, thermodynamically promoting the acceleration of specific pathways. Therefore, supramolecular engineering mainly ensures high selectivity of the oxygen reduction reaction from the two aspects of space and interface, by optimizing mass transfer, exposing sites, and regulating charge flow (Fig. 18b) [133].

Modulating edge sites achieves fine control over the activity and position of active sites. Catalytic performance not only depends on the intrinsic activity of the site but is also closely related to its position in space and local electronic structure. It has been found that even in one-dimensional COFs with the same structure, the single-atom Pt site located inside the pore has higher anion transport efficiency and dynamic reconfiguration ability, thus showing a much higher intrinsic activity than the surface sites. The chemical composition at the edge directly determines the strength of its interaction with reaction intermediates. For example, researchers systematically introduce functional groups such as phenyl imidazole, which can optimally regulate the adsorption energy of OOH*, thereby screening out active sites suitable for high-selectivity H_2_O_2_ production. Furthermore, by modifying the charges of the edge units, the electronic cloud density of the active center can be directly changed, successfully achieving the precise switching from the 4e^−^ to the 2e^−^ ORR pathway (Fig. 18c). Additionally, the deliberately constructed edge defects themselves can become new highly active centers. The unique electronic structure of the adjacent carbon atoms opens up new pathways for achieving nearly 100% selectivity in catalytic activity. The characteristic of modulating edge sites lies in precise control, which directly manipulates the microscopic interactions between the reaction center and intermediates. It is the most direct means to achieve path-selective regulation.

The building catalytic linkage, starting from the fundamental elements of the chemical bonds that constitute the COF, through rational design of the types of connection bonds, chemical microenvironment, and topological structure, controls the electronic properties of the material from the source and ensures a specific reaction microenvironment (Fig. 18d). Besides the traditional metal active centers, the connection bonds themselves can also serve as catalytic active centers. Different connection bonds have different tendencies for the reaction pathways of oxygen reduction reactions. For example, compared to imine bonds, the carbon atoms in oxazole connection bonds can more effectively promote the formation of OOH* intermediates, thus exhibiting excellent 2e^−^ ORR activity. While amine connection bonds tend to promote the breaking of O–O bonds and guide the 4e^−^ pathway due to their tendency to form hydrogen bonds with oxygen molecules. Therefore, the precise selection of connection bonds is the basis for regulating reaction pathways. On this basis, fine regulation, such as introducing bromine substituents on the linking units or adjusting the length of the polyether side chains, reduces the capacity of this material and its hydrophilicity/hydrophobicity can be systematically controlled, thereby achieving fine-tuning of catalytic activity and selectivity. Finally, in terms of structural innovation, developing new connection methods such as ladder-like topological structures can significantly enhance the electronic delocalization capability and structural stability of materials, enabling simultaneous efficient catalysis of multiple reactions.

### Structural Reconstruction, Stability, and Emerging Characterization Techniques

Unlike rigid inorganic catalysts, the electrocatalysts based on COFs may undergo dynamic structural reconfiguration under the working potential. This reconfiguration can be detrimental, leading to a decrease in activity, or it can be beneficial, generating true active substances. Therefore, understanding the interaction between reconfiguration and long-term stability is of crucial importance. This section discusses the phenomenon of structural evolution, the resulting stability challenges, strategies for enhancing durability, and the in situ characterization techniques required to monitor these dynamic processes.

Recent studies have begun to reveal the evolution caused by potential energy in COF-related systems, including the rearrangement of metal nodes [135], the change in the coordination environment at M–N_4_ sites, and the hydrolysis or reconfiguration of imine, azine, or other connecting bonds [136, 137]. However, compared to the extensive research on MOFs or inorganic catalysts, systematic studies on the specific reconfigurations of COF systems are still limited. This gap represents both challenges and opportunities for future research.

The long-term stability of COF-based electrocatalysts is a crucial but often under-studied factor in their practical applications. Currently, three main degradation pathways have been identified:

(i) Chemical hydrolysis at the bond connection sites: Reversible covalent bonds, such as imine bonds, boroxine bonds, and borate ester bonds are prone to hydrolysis in acidic or alkaline electrolytes, leading to the collapse of the framework structure. Imine bond-connected covalent organic frameworks are particularly vulnerable to damage in aqueous media, especially under the harsh pH conditions required for ORR/OER.

(ii) Metal leaching and reconstruction: In metalized covalent organic framework materials, coordination bonds formed with metal ions may break under long-term electrode polarization. Hosseini et al. [138] discovered under alkaline OER conditions that the cobalt ions in the cobalt-functionalized COF (TpBpy-Co) were released from the framework and transformed into cobalt oxide-based nanoparticles. These newly formed nanoparticles were the true active species, while the original COF merely served as a precursor catalyst.

(iii) Oxidation of the carbon skeleton: At high anode potentials, the organic framework of COFs is prone to oxidation. Additionally, free radical intermediates generated during catalysis can attack the framework structure, thereby affecting its long-term stability.

In addition to chemical stability, the mechanical stability of the electrode structure is also crucial. Traditional adhesive-based electrode preparation methods often lead to catalyst detachment, poor mass transfer, and increased resistance, thereby accelerating performance degradation.

In addition to chemical stability, the mechanical stability of the electrode structure is also crucial. Traditional adhesive-based electrode preparation methods often lead to catalyst detachment, poor mass transfer, and increased resistance, thereby accelerating performance degradation.

Table 7 presents the long-term stability data of representative oxygen electrocatalysts based on COF, including the test conditions and stability indicators, for the purpose of practical assessment.

**Table 7:** Stability performance of representative COF-based oxygen electrocatalysts

Material	Reaction	Electrolyte	Test Type	Stability Metric	References
CoOx@NC-800	ORR/OER	0.1 M KOH	CV cycling	100% retention after 3000 cycles	[76]
Fe,Co–N–C	ORR/OER	0.1 M KOH	CV cycling	negligible degradation after 4000 cycles	[92]
Co-COFs/CNT-700	Zn-air battery	6.0 M KOH	galvanostatic cycling	5.1% potential loss after 65 h	[9]
JUC-660	2e^−^ORR	flow cell	Chronoamperometry	H_2_O_2_ yield > 1200 mmol g^−1^ h^−1^, > 85 h	[104]
macro-TpBpy-Co	OER	0.1 M PB (pH 7)	CV cycling	1000 cycles stable	[26]
TpBpy-Co	OER	1.0 M KOH	post-electrolysis	Co^2+^ → CoO, nanoparticles (active species)	[138]
CoFc-MOF	OER	1.0 M KOH	in situ / ex situ	two-step restructuring to metal oxyhydroxide	[135]
Co-TpBpy-800 nanocages	ORR	0.1 M KOH	CV cycling	*E*_1/2_ = 0.831 V	[71]
NUST-38	OER/HER	1.0 M KOH	CV cycling	stable after 10,000 cycles	[89]
Imine-linked COF (general)	–	–	chemical stability	linkage engineering improves stability	[137]
TpBpy-CO	HER	–	electrochemical reconstruction	highly crystalline after reconstruction	[136]
DPPS-COF	ORR	0.1 M KOH	CV cycling	*E*_1/2_ = 0.85 V	[98]
B18C6-PTTA-COF	2e^−^ORR	0.1 M KOH	–	91% H_2_O_2_ selectivity	[97]
Edge-defect CON	2e^−^ORR	0.1 M KOH	–	> 99% H_2_O_2_ selectivity	[105]
Oxazole-linked COF	ORR	1.0 M KOH	–	stable C = C linkages	[108]
Nonpolar C = C linked COF	ORR	0.1 M KOH	–	excellent stability	[111]
FePc-BBL COF	ORR	0.1 M KOH	–	robust ladder network	[113]
Br-COF	2e^−^ORR	0.1 M KOH	–	86.2% H_2_O_2_ selectivity	[114]
COF-B	ORR	0.1 M KOH	–	*E*_1/2_ = 0.76 V	[115]
COF-C.N	OER	1.0 M KOH	–	349 mV overpotential	[53]
D-nt-A COF /3D graphene	ORR	0.1 M KOH	–	*E*_1/2_ = 0.880 V	[88]
lonic COF nanosheet	OER	1.0 M KOH	–	320 mV overpotential	[49]
1D COF	2e^−^ORR	0.1 M KOH	–	85.8% selectivity, TOF 0.051 s^−1^	[99]
Co-CNT@COF-Pyr	ORR/OER	1.0 M KOH	–	OER η 438 mV, n = 3.82–3.80	[90]
Fluorinated Co-porphyrin COF/GO	OER	1.0 M KOH	–	261 mV overpotential	[129]
Co@N-doped porous carbon	ORR/OER	–	Chronoamperometry	98.5% retention after 13 h	[74]

The feasibility of large-scale synthesis. The practical application of COF-based electrocatalysts still requires a scalable, cost-effective and environmentally friendly synthesis method. The traditional solvothermal method usually requires sealed containers, high temperature, long reaction time and toxic organic solvents, which limits the yield. Emerging methods such as microwave-assisted synthesis [139, 140], mechanical chemical grinding [141, 142] and flow chemistry [143] can achieve rapid and kilogram-scale COF production while maintaining its crystallinity and porosity. Interface polymerization on moving carriers and continuous flow reactors further allow the manufacture of COF films or membranes through roll-to-roll processes, which is crucial for membrane electrode assembly integration. Green solvents, such as ethanol/water mixtures, deep eutectic solvents and room-temperature synthesis, are also being explored to reduce energy and waste footprints. Although most of these technologies are still at the laboratory scale, pilot-scale production requires optimizing the uniformity of the reaction, heat/mass transfer and purification after synthesis. Future work should focus on developing standardized processes for large-scale COF synthesis and integrating COF processing, ink formulation, spray coating and scraper coating into industrial electrode manufacturing lines.

To capture these dynamic processes, advanced in situ characterization techniques are indispensable. For COF electrocatalysts, the emerging methods that are particularly relevant include:

In situ X-ray absorption spectroscopy (XAS) technology, which can monitor the changes in the oxidation state, coordination number, and bond length of metal centers during electrocatalytic processes. In situ Raman spectroscopy and Fourier-transform infrared spectroscopy (FTIR), which can track the evolution of organic functional groups, connecting bonds, and adsorbed intermediates [144]. In situ electrochemical quartz crystal microbalance (EQCM), which can provide real-time information on mass changes related to ion adsorption, structural expansion or dissolution/re-deposition processes. In situ electron paramagnetic resonance (EPR) technology, which can detect free radical intermediates and changes in the spin state of active sites [145].

These experimental techniques complement each other. DFT calculations have become indispensable for understanding the catalytic mechanism of COF-based electrocatalysts at the atomic level [9]. DFT calculations can elucidate the following key aspects: (i) electronic structure analysis, revealing the density of states (DOS), the center positions of *d* orbitals, and the charge density distribution that controls the adsorption strength of reaction intermediates. (ii) Gibbs free energy paths, which can determine the rate-determining steps and theoretical overpotential of oxygen reduction reaction (ORR)/oxygen evolution reaction (OER). (iii) Mechanistic insights into reaction selectivity, distinguishing between 2-electron and 4-electron pathways based on the adsorption energies of key intermediates (such as OOH* and OH*). (iv) screening and prediction of active sites—high-throughput DFT calculations can rapidly evaluate thousands of potential COF structures (such as different metal centers, coordination environments, and doping patterns), so as to identify the most promising candidates before experimental synthesis. For example, recent density functional theory studies on single-atom catalysts embedded in TQBQ-COF have shown that boundary oxygen atoms can interfere with the adsorption energy levels of reaction intermediates, thus breaking through the traditional proportional relationship that limits the efficiency of oxygen evolution reaction/oxygen reduction reaction.

The synergistic integration of in situ characterization and DFT calculations is highly effective. Specifically, the in situ XAS results can be directly correlated with the predicted coordination geometry and oxidation state of the DFT, thereby experimentally verifying the computational model. In situ Raman and Fourier-transform infrared spectroscopy can identify catalytic active species (such as metal oxides or hydrogen peroxide intermediates), while DFT calculations can explain their formation energy and reaction barrier. In situ EPR can detect free radical intermediates, and DFT calculations can provide insights into their spin density distribution and stability mechanism [146]. This method of combining experiments with calculations has been systematically reviewed in recent literature. In catalysts based on COF, the combination of advanced spectroscopy (XPS, XAS, Raman) and theoretical calculations (DFT, molecular dynamics) has been proved to be crucial for elucidating the structure–activity relationship [147].

If these techniques are combined with DFT calculations, they have great potential for analyzing the reconfiguration mechanism of COF-based electrocatalysts. For example, in situ XAS can reveal the evolution of coordination geometries related to potential, while in situ Raman spectroscopy can identify catalytic active species, such as metal–oxygen or peroxide hydrogen intermediates. This integrated approach not only deepens the understanding of the mechanism but also guides the design of more robust COF-based electrocatalysts that can resist unfavorable reconfigurations while taking advantage of beneficial dynamic changes [148].

### AI-assisted Mechanistic Understanding and Rational Design

In addition to traditional experimental methods and density functional theory-based approaches, artificial intelligence and machine learning (ML) are gradually emerging as powerful tools for accelerating mechanism discovery and guiding the rational design of COF-based electrocatalysts. The strategies based on artificial intelligence can be applied in two complementary directions:

1. Revealing the structure–activity relationship. Using training datasets from density functional theory calculations or experimental data, machine learning models such as random forests, support vector machines, and graph neural networks (GNNs) can be trained to predict key catalytic indicators, such as the adsorption energies and overpotential of *O, *OH, and *OOH. Feature importance analysis reveals which structural features (such as coordination number, electronegativity of substituents, or pore size) have the greatest impact on catalytic activity, thereby providing interpretable mechanistic insights, complementing traditional electronic structure analysis [149].
2. Accelerating the rational design of active sites. High-throughput virtual screening using machine learning can rapidly evaluate thousands of hypothetical COF structures, thereby screening out the best ORR/OER candidates before experimental synthesis. Generative models including variational autoencoders and reinforcement learning can achieve reverse design: proposing new building units or connection patterns to achieve the expected adsorption performance [150]. These methods have successfully predicted previously unexplored COF combinations with higher activity.

Although the application of artificial intelligence in the field of COF-based electrocatalysis has broad prospects, practical applications still face many challenges: limited high-quality datasets, poor transferability between different COFs, and the need for experimental validation. Future research should focus on establishing specialized COF-based electrocatalysis databases, combining active learning with automated synthesis, and combining machine learning with in situ characterization to complete the entire process of prediction and validation [151].

## Summary and Prospects

### Summary

This review provides a detailed introduction of four categories of COF based on their nature of the catalytic active sites when catalyzing OER and HER. The active sites of porphyrin COF are clearly defined and adjustable. Different central metals result in different catalytic performances. The porphyrin structure has good electrical conductivity. Cobalt porphyrin is the most efficient oxygen electrocatalyst among them. Metal-based COFs do not contain porphyrins. They form active centers by adding metal ions after synthesis. Commonly used coordination units include Bipyridine and pyrimidine. These materials have three development trends: first, not only should the conductivity be improved through thermal decomposition, but also the structural damage should be avoided; second, from single-metal active centers to dual-metal cooperative centers; third, from studying chemical composition to studying physical structure. For metal-free COFs, the catalytic activity stems from the framework itself or heteroatoms. Performance arises from the regulation of electronic structure and local environment. Current research focuses on introducing heteroatoms, unsaturated bonds, and constructing D-A structures, with the core emphasis on modulating electronic structure. Electronic regulation is shifting from passive control to active design, primarily centered on D-A structures. Additionally, spatial configurations, linkage modes, cation-*π* interactions, and alkoxy side chains are also key research areas. Additionally, radical COFs contains stable free radicals, has a unique electronic structure, is easy to synthesize, has good stability, and can promote the four-electron ORR process. Currently, there is relatively little research on this topic. The early work mainly focused on clarifying its definition, verifying its catalytic activity and electron transfer characteristics, and using free radical sites to regulate the ORR pathway.

In the design of COF structures, the strategies for enhancing the catalytic performance of oxygen are mainly divided into three categories, focusing on the regulation of active sites, electronic structure, and microenvironment. The first category focuses on the design and optimization of active sites, including metal anchoring, modulating edge sites, and construction of bifunctional COFs. Metal anchoring introduces metal active centers through coordination, and is evolving from a single metal to a dual-metal and composite system, regulation of edge sites enhances site activity or creates new sites by changing edge components, creating defects, or regulating spatial positions, construction of bifunctional materials integrates different active sites to form a synergistic catalytic system. The second category focuses on the regulation of electronic structure and microenvironment, including polarization strategies and methods of building catalytic linkage. Polarization strategies introduce electron donor or acceptor groups to induce asymmetric charge distribution, and constructing D-A structures has become the main means of shifting from passive to active control of charge transfer, methods of building catalytic linkage define the electronic properties of COF from the root by selecting connection bond types, substituents, and topological structures, and regulate the hydrophobicity and energy band structure of the microenvironment. The third category is supramolecular engineering, which is a current frontier strategy. By regulating non-covalent interactions, it designs interlayer stacking, size, and heterointerfaces on a larger scale, thereby optimizing the material and charge transfer pathways and increasing the spatial exposure of active sites.

In the COF functional synthesis, three main strategies are employed. The pre-modification strategy involves designing organic monomers containing the target functional groups before synthesis and then directly polymerizing them into COF. This strategy enables precise structural control, electronic regulation, bimetallic catalysis, and D-A structure construction. The in situ modification strategy involves simultaneously introducing or generating a second component during synthesis, which is used to composite other materials or induce structural transformation. Recent studies have begun to utilize physical fields such as radiation to drive the modification process. Post-synthesis modification involves introducing functional groups or active centers through chemical reactions onto the synthesized COF, which is a primary means for achieving metal anchoring, structural regulation, and dual-mode materials. In recent years, more refined strategies such as stepwise post-modification have also been developed.

More importantly, this review summarizes the underlying mechanisms of three strategies for enhancing catalytic performance. The synergistic effect mechanism of active site design lies in the heteronuclear bimetallic system generates charge transfer due to the difference in electronegativity, adjusts the *d* orbital positions of the metal centers, and provides the optimal configuration for different reaction intermediates. The core mechanism of electronic structure regulation is to break symmetry, including the coordination field and electron distribution, thereby forming the active site with the optimal adsorption strength for the intermediates and regulating the *p* orbital center. The control mechanism of the reaction pathway is a multi-level dynamic process, with the core being to achieve it by regulating the adsorption energy of key intermediates. Weakening the O–O bond promotes the 4-electron pathway, while stabilizing OOH* promotes the 2-electron pathway.

### Prospects

Although significant progress has been made in the structural design and performance control of COFs based electrocatalysts, there are still a series of challenges when applying them to practical oxygen electrocatalysis applications.

1. There is a contradiction between high activity and high stability. Strategies to enhance activity, such as polarization and high-activity metal centers, often come at the expense of chemical stability and are difficult to adapt to harsh environments like strong acids, strong bases, or high oxidation potentials. How to balance high activity and high stability is one of the current core challenges. To address this contradiction, we propose using connection key engineering methods [116] such as amide bonds or *sp*^*2*^ carbon bonds, employing protective layer encapsulation techniques, such as carbon coatings or MOF coatings, and identifying the pathways leading to material degradation through real-time monitoring of the reaction process, thereby achieving targeted stabilization treatment.
2. There is a contradiction between conductivity and porous structure. High catalytic activity requires a high specific surface area and complete multi-level pores, but the commonly used pyrolysis method to enhance conductivity will damage crystallinity and pore structure. Therefore, fundamentally improving the conductivity of COF without sacrificing the ordered pores is another key challenge. Promising solutions include in situ growth on conductive carbon scaffolds, designing COFs with intrinsic conductivity through extended *π* conjugation structures or D-A units, and using post-modification doping methods that do not disrupt the crystal structure.
3. The atomic-level precise construction and characterization of the active sites remain challenging. Although COF can be designed, there is still randomness in edge defects, doping atom positions, and the microenvironment of connection bonds. At the same time, in situ characterization techniques are not yet mature, and the catalytic mechanism mostly relies on speculation. The active sites of non-metallic COF [152] and the performance evaluation system also need to be improved. To overcome these limitations, we recommend using precisely controlled defect engineering methods, combining in situ spectroscopic analysis techniques [153] such as Raman spectroscopy, XAS, FTIR with density functional theory calculations, and applying machine learning to predict microscopic environmental parameters and formulating standardized performance evaluation protocols.
